# A modular UDP‐glycosyltransferase and cytochrome P450 system for soyasaponin C‐22 modification

**DOI:** 10.1111/tpj.71149

**Published:** 2026-10-16

**Authors:** Susumu Hiraga, Yukiko Fujisawa, Panneerselvam Krishnamurthy, Hae‐Reon Son, Soo Yeon Chung, Hayato Suzuki, Ryu Nakata, Yoshikazu Shimoda, Ryoichi Yano, Akito Kaga, Chigen Tsukamoto, Hikaru Seki, Toshiya Muranaka, Masao Ishimoto

**Affiliations:** ^1^ Institute of Crop Science National Agriculture and Food Research Organization (NARO) Tsukuba Ibaraki 305‐8518 Japan; ^2^ Institute of Agrobiological Sciences NARO Tsukuba Ibaraki 305‐8634 Japan; ^3^ Faculty of Agriculture Iwate University Morioka Iwate 020‐8550 Japan; ^4^ Graduate School of Engineering The University of Osaka Suita Osaka 565‐0871 Japan; ^5^ Research Centre for Advanced Analysis NARO Tsukuba Ibaraki 305‐8602 Japan; ^6^ Institute for Open and Transdisciplinary Research Initiatives The University of Osaka Suita Osaka 565‐0871 Japan; ^7^ Present address: BRIC–National Agri‐Food and Biomanufacturing Institute (NABI) Mohali Punjab 140306 India; ^8^ Present address: The Biomanufacturing Process Research Center The National Institute of Advanced Science and Technology Tsukuba Ibaraki 305‐8566 Japan; ^9^ Present address: Institute of Crop Science National Agriculture and Food Research Organization (NARO) Tsukuba Ibaraki 305‐8518 Japan

**Keywords:** soybean, saponin, co‐expression, gene cluster, cytochrome P450, UDP‐glycosyltransferase, DDMP, microsynteny, *Lotus japonicus*

## Abstract

Soybean (*Glycine max* (L.) Merr.) produces triterpenoid saponins, called soyasaponins, which are categorised as 2,3‐dihydro‐2,5‐dihydroxy‐6‐methyl‐4*H*‐pyran‐4‐one (DDMP) saponins and group A saponins based on the chemical modifications at the C‐22 position of their triterpene skeleton. These C‐22 modifications confer different biological functions. While most genes involved in soyasaponin biosynthesis have been identified, the enzymes responsible for conjugation of DDMP or arabinose to the C‐22 hydroxyl group remain unclear. This study aimed to identify the enzymes involved in these modifications. Co‐expression analysis using known saponin biosynthetic genes identified three candidate *UGT* genes. Among them, *UGT73K5* was essential for DDMP saponin biosynthesis in soybean, as *ugt73k5* mutants lacked these compounds. Similarly, the orthologous gene in *Lotus japonicus*, *UGT73K6*, was required for DDMP saponin biosynthesis, and the *ugt73k6* defect was complemented by soybean *UGT73K5*. In addition, *UGT73BU1*, a homologous gene not co‐expressed with known saponin biosynthetic genes, was identified as a C‐22 arabinosyltransferase involved in group A saponin biosynthesis. In *L. japonicus*, *UGT73K6* is genomically co‐localised and co‐expressed with a cytochrome P450 gene responsible for C‐22 hydroxylation, forming a conserved biosynthetic gene cluster. This organisation suggests coordinated biosynthetic modules underlying DDMP saponin production in legumes.

## Significance Statement

C‐22 modifications determine soyasaponin functions, but the responsible enzymes have remained elusive. We identified soybean UGT73K5 and *Lotus japonicus* UGT73K6 as enzymes involved in DDMP conjugation and soybean UGT73BU1 as the C‐22 arabinosyltransferase; together with the co‐expressed, genomically clustered CYP72A61‐UGT73K6 module, these findings reveal a modular cytochrome P450‐UDP‐glycosyltransferase system underlying soyasaponin C‐22 modification.

## INTRODUCTION

Soybean (*Glycine max* (L.) Merr.) is an important source of nutrition for humans and livestock. Its seeds contain abundant macronutrients—including proteins, oils, carbohydrates, dietary fibres and minerals—as well as specialised metabolites such as isoflavones, α‐tocopherol, anthocyanins and saponins. Saponins are triterpenoid compounds comprising 30‐carbon skeletons derived from six isoprene units and typically undergo extensive glycosylation (Thimmappa et al., 2014). They accumulate not only in seeds but also in leaves and roots; however, their physiological functions in plants are not yet fully understood. Some saponins contribute to defence against root‐infecting fungi (Lacchini et al., 2023; Osbourn, 1996; Papadopoulou et al., 1999) and herbivores (Kuzina et al., 2009), whereas others participate in root nodulation (Confalonieri et al., 2009). Tsuno et al. (2018) reported that saponins are exuded from the roots of several legume species, including soybean, *Lotus japonicus*, alfalfa (*Medicago sativa*), and pea (*Pisum sativum*). In soybean, soyasaponin Bb, one of the saponins exuded from roots, was later shown to diffuse into the rhizosphere and modulate the soil microbiome (Fujimatsu et al., 2020), suggesting its potential role as a mediator in plant–microbe interactions.

Saponin biosynthesis begins with the cyclisation of 2,3‐oxidosqualene, a precursor derived from the mevalonate pathway, by specific oxidosqualene cyclases, yielding triterpene skeletons with diverse chemical structures (Vincken et al., 2007). These cyclic skeletons are subsequently modified by cytochrome P450 monooxygenases (CYPs) through site‐specific oxygenation, and UDP‐glycosyltransferases (UGTs) through sugar conjugation. The attached sugar moieties may undergo additional modifications such as acetylation (Shiraiwa et al., 1991) or malonylation (Kochkin et al., 2013). Together, these sequential and combinatorial modifications generate extensive structural diversity among saponins.

Soyasaponins are oleanane‐type triterpenoid saponins produced by soybean and other legume species. They are broadly classified into 2,3‐dihydro‐2,5‐dihydroxy‐6‐methyl‐4*H*‐pyran‐4‐one (DDMP) saponins and group A saponins based on modifications at the C‐22 position. DDMP saponins possess a sugar chain at the C‐3 hydroxyl group and form a hemiacetal linkage with the DDMP moiety at the C‐22 position of soyasapogenol B. In contrast, group A saponins are derived from soyasapogenol A, possess a hydroxyl group at the C‐21 position and two sugar chains at the C‐3 and C‐22 positions. These structural differences confer distinct biological properties (Yoshiki et al., 1998). For instance, DDMP saponins and their derivatives (groups B and E saponins) have been associated with health‐promoting effects, including cholesterol‐lowering activity, lipid peroxidation inhibitory effects, and hepatoprotective functions (Guang et al., 2014). By contrast, group A saponins are responsible for the bitter aftertaste of soybean‐derived products (Okubo et al., 1992). Tissue‐specific distribution patterns further underscore their functional divergence: DDMP saponins accumulate in roots, leaves, seed hypocotyls and cotyledons, whereas group A saponins are mainly detected in roots and hypocotyls (Krishnamurthy et al., 2019; Tsuno et al., 2018), suggesting that biosynthesis or transport of these saponins is spatially regulated.

The soyasaponin biosynthetic pathway has been extensively investigated in soybean. The first molecular cloning of the soyasaponin biosynthetic enzyme gene *CYP93E1* was achieved through expressed sequence tag mining and functional characterisation *via* heterologous expression in yeast (Shibuya et al., 2006). Subsequent cDNA cloning and enzymatic analyses identified two UGTs, GmSGT2 (UGT73P2) and GmSGT3 (UGT91H9), which catalyse the sequential transfer of galactose to soyasapogenol B monoglucuronide (SBMG) and rhamnose to the galactosylated SBMG (Shibuya et al., 2010).

Molecular genetic studies revealed additional *CYPs* and *UGTs* involved in soyasaponin biosynthesis. Four natural mutations, *sg‐1*, *sg‐3*, *Sg‐4* and *sg‐5*, have been identified in soybean germplasm, and their causal genes were determined by positional cloning. The *Sg‐1* locus, which regulates the addition of a second sugar at the C‐22 position of group A saponins, comprises three alleles: *Sg‐1* ^*a*^ (*UGT73F4*, xylosyltransferase), *Sg‐1* ^*b*^ (*UGT73F2*, glucosyltransferase), and a loss‐of‐function allele *sg‐1* ^0^ (Sayama et al., 2012). *Sg‐3* encodes UGT91H4, which adds glucose as the third sugar at the C‐3 position (Yano et al., 2018), whereas *Sg‐4* encodes UGT73P10, which is responsible for arabinose addition as the second sugar at the same position (Takagi et al., 2018). *Sg‐5* encodes CYP72A69, which catalyses C‐21 hydroxylation (Yano et al., 2017). In *sg‐5* mutants, group A saponins are absent, and DDMP saponins are increased, indicating that CYP72A69 mediates a key branching step in the pathway.

Many soybean mutants with altered seed saponin composition have been identified by screening ethyl methanesulphonate (EMS)‐induced populations. Krishnamurthy et al. (2019) screened 909 mutant lines using TLC and identified 35 mutants, which were categorised into seven classes. Soyasaponin biosynthetic genes, including *β‐amyrin synthase1* (*bAS1*, Takagi et al., 2011), *CYP93E1* (Shibuya et al., 2006), and *CYP72A61* (Ebizuka et al., 2011), were confirmed as causal genes for several mutants through liquid chromatography–photodiode array/tandem mass spectrometry (LC‐PDA‐MS/MS) and genetic analyses. Additional mutant alleles of *UGT73F2* (*Sg‐1*) were also identified. Moreover, three novel mutants with unknown causative genes have been reported, including those (1) lacking DDMP saponins, (2) producing predicted group‐A‐saponin‐like compounds with free C‐22 hydroxyl groups, and (3) producing saponins with missing second and third sugars at the C‐3 position (suggesting defects in specific conjugation steps). Sundaramoorthy et al. (2019) identified a DDMP‐saponin‐deficient mutant and proposed a novel UGT as the DDMP transferase; however, its function was not validated because complementation analysis was not performed.

Studies in *Arabidopsis*, *L*. *japonicus*, *M. truncatula*, and cucurbits such as cucumber, melon and watermelon have shown that genes involved in the biosynthesis of saponins and other triterpenoids are often co‐expressed (Field & Osbourn, 2008; Fukushima et al., 2013; Huang et al., 2019; Krokida et al., 2013; Ribeiro et al., 2020; Zhou et al., 2016). These findings suggest that triterpenoid biosynthetic genes are regulated within coordinated gene networks, enabling the identification of previously uncharacterised pathway genes through co‐expression analysis. In soybean, CSyGT1, an enzyme that transfers glucuronic acid as the first sugar at the C‐3 position, was identified using this approach (Chung et al., 2020). More recently, Yuan et al. (2023) applied the same strategy to identify candidate acetyltransferases involved in the biosynthesis of group A soyasaponin in soybean. These studies demonstrated that co‐expression analysis is a powerful approach for discovering previously uncharacterised genes involved in soyasaponin biosynthesis.

Although most steps of the soyasaponin biosynthetic pathway have been elucidated, the enzymes responsible for C‐22 modifications, including DDMP conjugation and arabinosylation, remain poorly characterised. Because these modifications determine the structural diversity and biological properties of soyasaponins, identifying responsible enzymes is essential for understanding soyasaponin biosynthesis and its impact on soybean quality and functionality. In this study, we identified and functionally characterised the enzyme responsible for C‐22 DDMP conjugation by combining co‐expression analysis in soybean with complementation assays in *L. japonicus*, which produces DDMP saponins through a conserved biosynthetic pathway. We further investigated the genomic organisation of genes encoding the enzymes involved in the C‐22 hydroxylation and DDMP conjugation. Our findings provide new insight into the molecular basis and evolution of C‐22 modification in legume soyasaponin biosynthesis.

## RESULTS

### Identification of candidate C‐22 UGTs by co‐expression analysis

To identify previously uncharacterised UGTs involved in soyasaponin biosynthesis, we searched for genes co‐expressing with known soyasaponin biosynthetic genes. We analysed SoyNet (https://www.inetbio.org/soynet/), a functional gene network constructed by integrating genomic and evolutionary information with gene expression data from 734 microarray and 290 RNA‐seq samples (Kim et al., 2017). Based on a set of 10 known genes encoding bAS1, CYPs, CSyGT and UGTs (Figure 1a,b; Table S1), we searched co‐expressed candidate genes potentially involved in soyasaponin biosynthesis. At a Z‐score threshold of 250 (Berger et al., 2007), the analysis identified a network consisting of eight known biosynthetic genes and three genes representing previously uncharacterised components of the soyasaponin biosynthetic pathway (Figure 1b). All three uncharacterised genes (*Glyma.15G221300, Glyma.16G033700*, and *Glyma.18G208300*) were predicted to encode UGTs. Like known biosynthetic genes, these predicted *UGTs* were preferentially expressed in seeds, roots and root hairs than in the pods, flowers or leaves (Figure S1; Table S1). Their strong co‐expression with established soyasaponin biosynthetic genes suggested that they participate in soyasaponin biosynthesis. To test this possibility, we examined the effects of mutations in each *UGT* gene on saponin composition in soybean.

**Figure 1:**
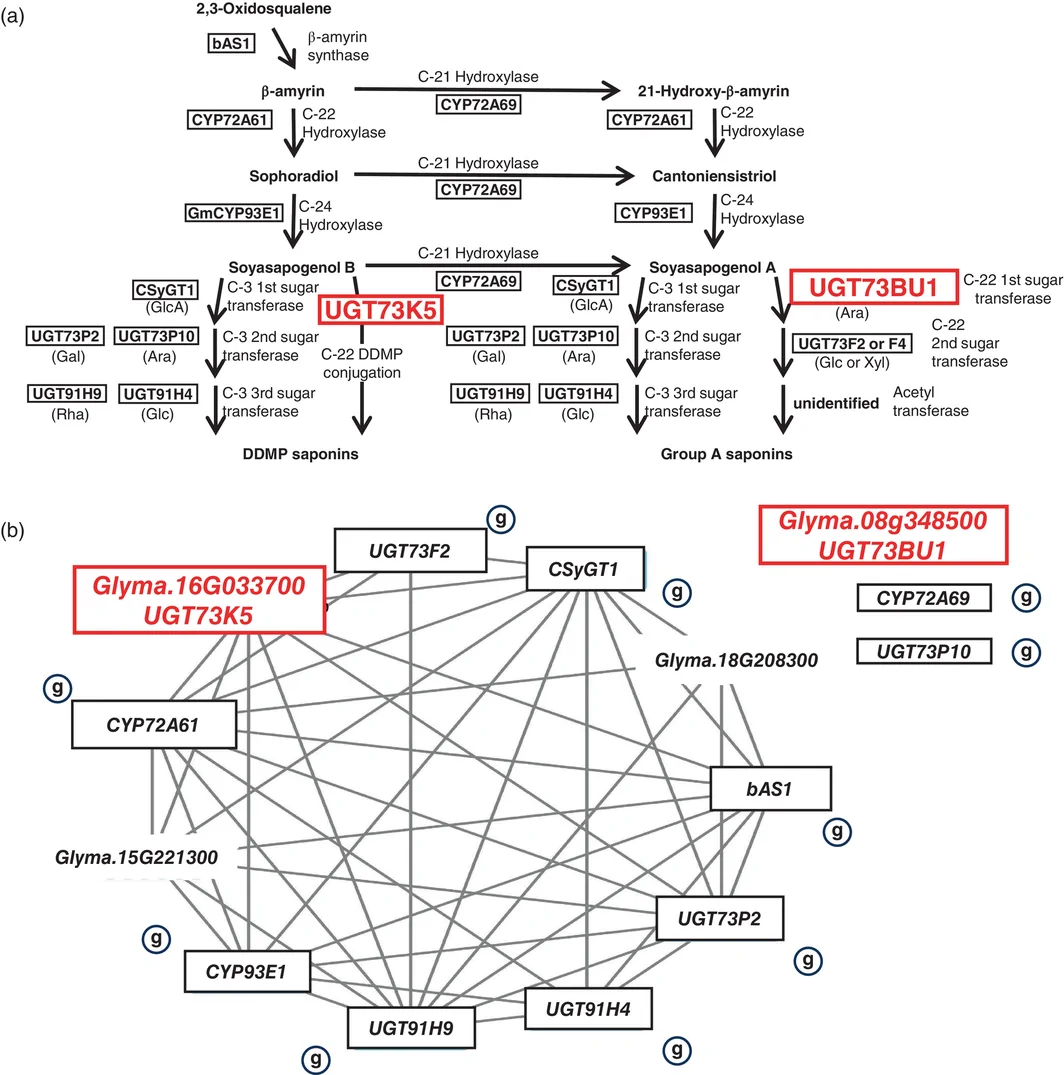
Co‐expression analysis to identify uncharacterised *UGT* genes involved in the biosynthesis of soyasaponins. (a) The proposed soyasaponin biosynthesis pathway in soybean seeds. Both common enzyme names and systematic enzyme names (shown in boxes) are indicated alongside the arrows representing each step in the pathway. bAS, β‐amyrin synthase; CSyGT, cellulose synthase superfamily‐derived glycosyltransferase; CYP, cytochrome P450 monooxygenase; UGT, uridine 5′‐diphospho‐glycosyltransferase. The two UGTs that were characterised in this study, UGT73K5 and UGT73BU1, are highlighted in red. The sugar moieties transferred by each UGT are indicated in parentheses below the corresponding enzyme names: glucuronic acid (GlcA), galactose (Gal), arabinose (Ara), rhamnose (Rha), glucose (Glc) and xylose (Xyl). (b) Soyasaponin biosynthetic gene network identified by co‐expression analysis in soybean. Genes encoding biosynthetic enzymes are enclosed in boxes with the same border styles as in panel (a); gene IDs without boxes represent *UGT* genes with uncharacterised functions. Guide genes used for the SoyNet search (Option III: Find Functional Modules) are marked with a circled ‘g’. Genes not included in the co‐expression network are shown in the top right‐hand corner.

### Saponin composition of loss‐of‐function mutants of candidate *UGTs*

To investigate the functions of the three candidate *UGT* genes identified by co‐expression analysis (Figure 1b), we performed reverse genetic screening using a high‐density soybean mutant library (Tsuda et al., 2015). Screening of DNA pools from 909 mutant lines identified 51 lines carrying 45 unique missense mutations in *Glyma.16G033700* (Table S2). Although no nonsense mutations were recovered, 10 lines carrying nine distinct amino acid substitutions lacked DDMP saponins. Seven of these corresponded to previously reported category 3 mutants identified by forward genetic screening (Krishnamurthy et al., 2019), while three lines (EnT‐0051, 2016 and 3528) were newly identified in this study. In wild‐type hypocotyls and/or cotyledons, UV chromatograms showed peaks corresponding to soyasaponin Ab (28.3 min) and DDMP saponins αg (36.3 min), βg (37.4 min), and βa (38.7 min) (Figure 2a,b; Figure S3). In contrast, these DDMP saponin peaks were absent or markedly reduced in *Glyma.16g033700* mutants (Figure 2A; Figure S4). Acid hydrolysis of mutant extracts yielded soyasapogenol B in these mutants (Figure S4), indicating that the mutants retained the ability to synthesise soyasapogenol aglycones but were defective in DDMP conjugation.

**Figure 2:**
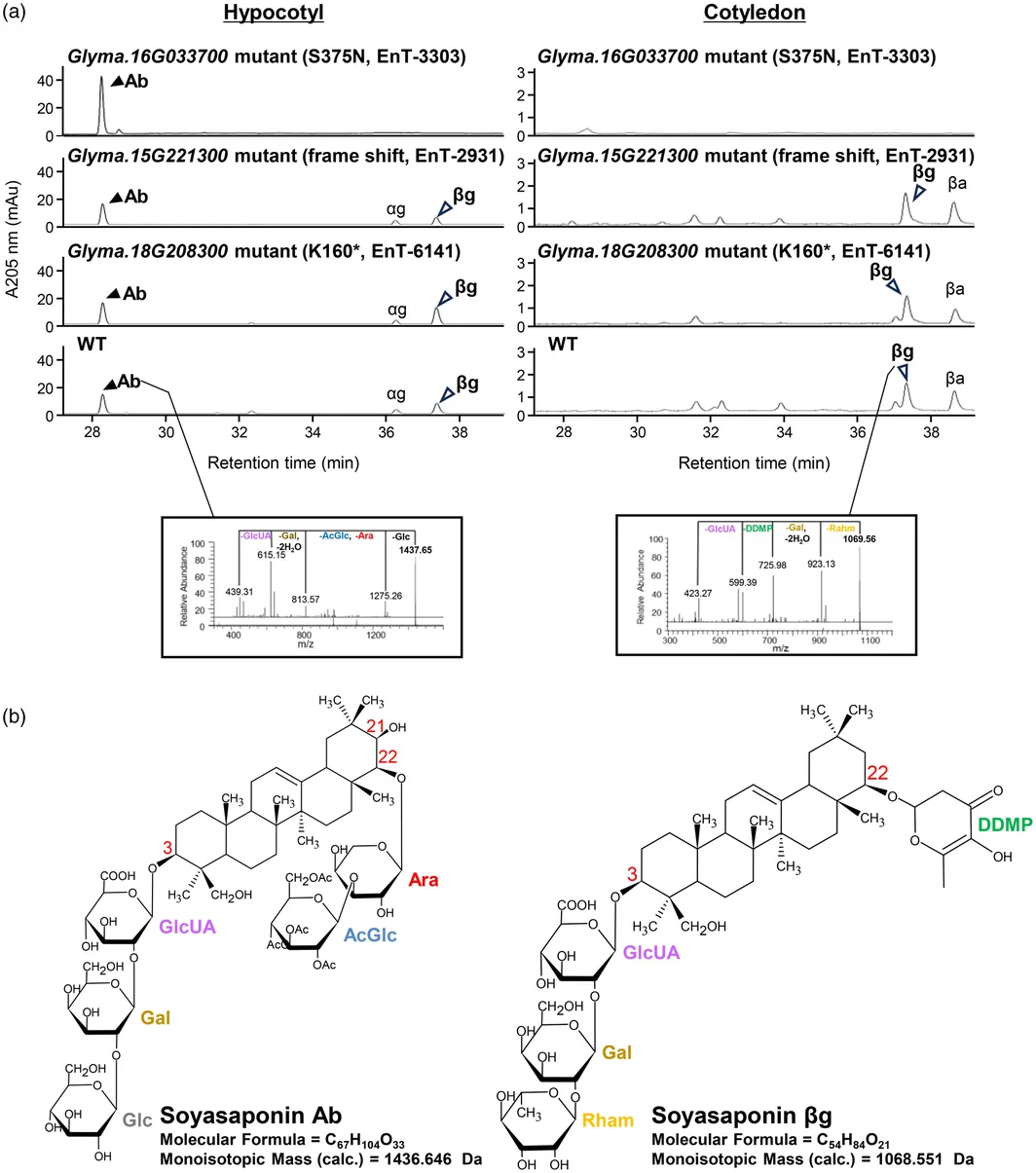
LC‐PDA‐MS/MS analysis of extracts from the seeds of soybean lines that carry loss‐of‐function mutations in *UGT* genes, which are co‐expressed with characterised soyasaponin biosynthetic enzyme genes. (a) UV chromatograms (205 nm) of hypocotyl and cotyledon extracts from soybean seeds carrying mutations in candidate *UGT* genes are shown. These include a missense mutation in *Glyma.16G033700* and a nonsense mutation in the other candidate genes. The major soyasaponin peaks (Ab, αg, βg and βa) are marked. Representative MS and MS/MS spectra of soyasaponins Ab (retention time, 28.3 min) and βg (37.4 min) from wild‐type seeds (WT, cultivar Enrei) are shown below the chromatograms. (b) Chemical structures of soyasaponins Ab and βg. The sugar moieties and the DDMP moiety are colour‐coded to correspond to the MS/MS fragment ions shown in panel (a), and the functionally important hydroxylation sites (C‐21 and C‐22) are marked with red numbers.

For the two other candidate genes, *Glyma.15g221300* and *Glyma.18g208300*, 25 and 12 mutant lines were identified, respectively. Among them, EnT‐2931 (*Glyma.15g221300*) and EnT‐6141 (*Glyma.18g208300*) carried frameshift and nonsense mutations, respectively, both of which are likely to generate loss‐of‐function alleles (Tables S3 and S4; Figure S2). However, neither mutant showed detectable changes in the accumulation of group A or DDMP saponins (Figure 2a). These results identified *Glyma.16G033700* as the only candidate gene associated with DDMP saponin biosynthesis, and subsequent analyses therefore focused on this gene.

### Genetic complementation of a DDMP saponin‐deficient *L. japonicus* mutant by *Glyma.16G033700*

Although *Glyma.16g033700* encodes a predicted UGT involved in the conjugation of DDMP at the C‐22 hydroxyl group, its enzymatic activity could not be verified *in vitro* due to the unavailability of UDP‐DDMP and absence of activity when the sugar donors (UDP‐glucose, UDP‐galactose, UDP‐glucuronic acid, and UDP‐arabinose) were tested under our assay conditions. We therefore employed the model legume *L. japonicus*, which also produces DDMP saponins (Tsuno et al., 2018), to validate its function *in vivo*. A BLASTP search of the *L. japonicus* genome database in Lotus Base identified *Lj3g3v3776600* as the closest homologue of *Glyma.16g033700* (Table S5). Its expression profile closely resembled those of known *L. japonicus* saponin biosynthetic genes *OSC1* (*Lj3g3v2027430*; β‐amyrin synthase; Sawai et al., 2006), *CYP93E1* (*Lj1g3v3555800*; C‐24 hydroxylase; Suzuki et al., 2019), *CSyGT1* (*Lj3g3v1981230*; C‐3 glucuronosyltransferase, Chung et al., 2020), and a *CYP72A61* homologue (*Lj3g3v3776580*, see below) (Figure S11; Tables S8 and S10). Therefore, we analysed the saponin composition of *L. japonicus* lines with retrotransposon (*LORE1*) insertions in *Lj3g3v3776600* (Figure 3a). The βg peak (37.4 min), confirmed by MS/MS analysis (Figure 3b), was detected in the wild‐type but absent from two independent *LORE1* mutants (lines 1 and 2; Table S6), indicating that *Lj3g3v3776600* is required for DDMP saponin biosynthesis.

**Figure 3:**
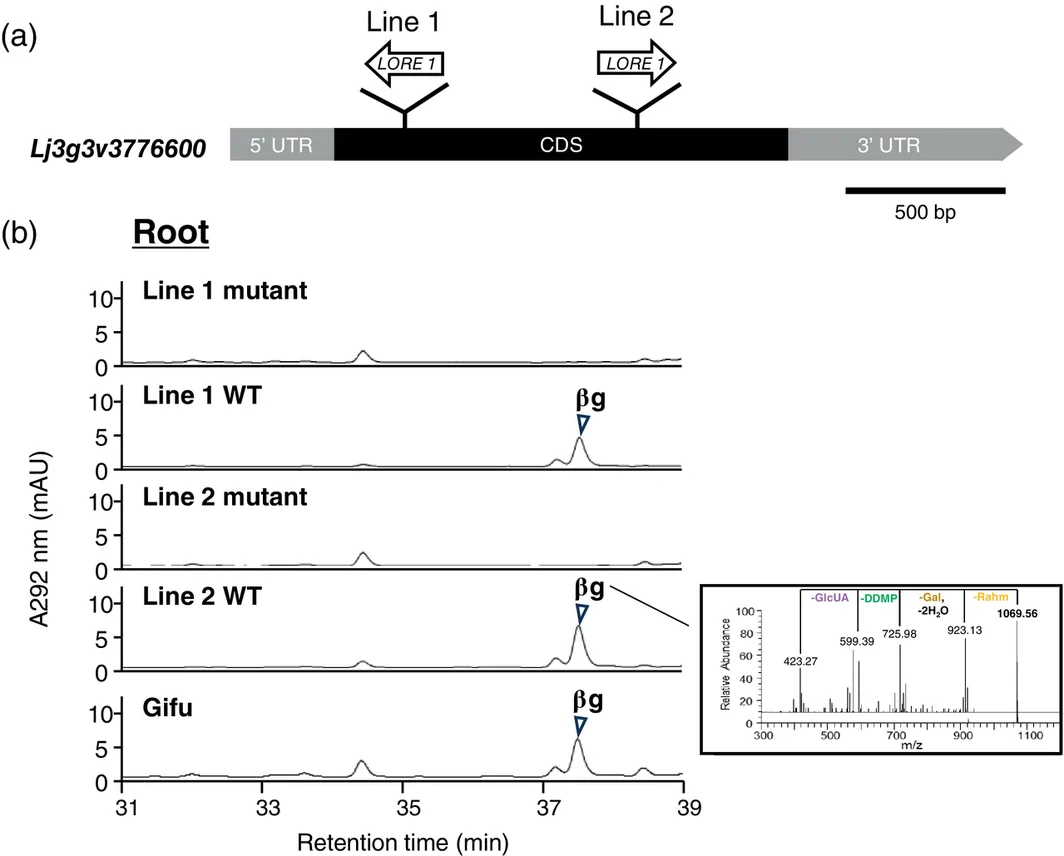
The loss of DDMP saponins in *Lotus japonicus* mutants carrying retrotransposon insertions in a *UGT* gene orthologous to *Glyma.16G033700*. (a) A schematic representation of the *Lj3g3v3776600* gene. The insertion sites of *Lotus retrotransposon 1* (*LORE1* indicated by an arrow) in two independent mutant lines (Line 1: Lotus Base plant ID 30058798; Line 2: ID A01254) are shown as Y‐shaped symbols above the protein‐coding sequence (CDS, shown in a black box). (b) LC‐PDA‐MS/MS analysis of root extracts from *Lj3g3v3776600* mutants. UV chromatograms (292 nm) of root extracts from homozygous *LORE1* insertion mutants (mutant) and their homozygous wild‐type siblings (WT) from segregating populations are shown. Representative MS and MS/MS spectra of soyasaponin βg which were detected in the root extracts of a wild‐type plant are shown to the right of the chromatograms.

To determine whether the soybean gene could complement this defect, we generated transgenic hairy roots expressing *Glyma.16g033700*, *Lj3g3v3776600* or an empty vector under the control of the CaMV 35S promoter, each carrying a GFP marker (Figure 4a). As shown in Figure 4(c), βg accumulated in wild‐type roots and was restored in the mutant lines transformed with either *Glyma.16g033700* or *Lj3g3v3776600*, whereas no complementation was observed in the empty vector control. These results demonstrate that *Glyma.16g033700* and *Lj3g3v3776600* encode functionally equivalent UGTs responsible for DDMP conjugation at the C‐22 position of soyasaponins *in vivo*. Although Glyma.16G033700 was previously proposed as a candidate DDMP transferase (Sundaramoorthy et al., 2019), our complementation analysis provides direct genetic evidence confirming its role in DDMP saponin biosynthesis. Accordingly, we designated *Glyma.16G033700* and *Lj3g3v3776600* as *UGT73K5* and *UGT73K6*, respectively, in accordance with the guidelines of the UGT nomenclature committee (Mackenzie et al., 1997).

**Figure 4:**
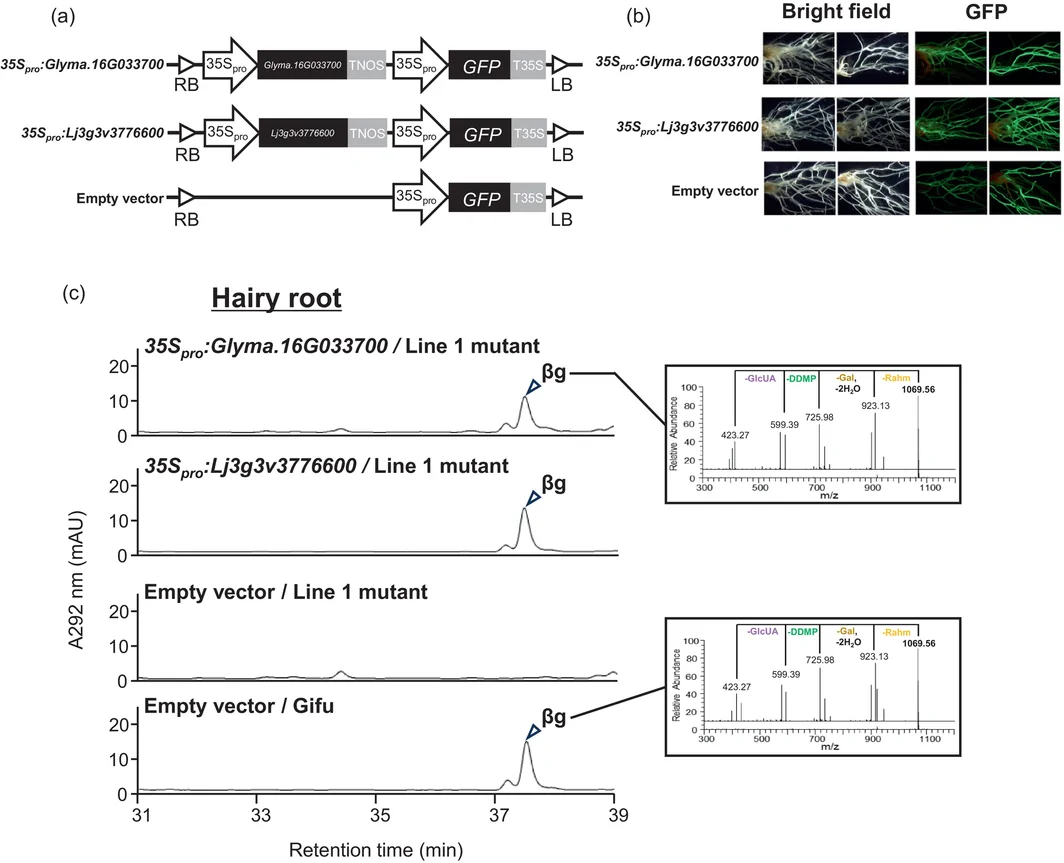
Complementation of the *Lj3g3v3776600* mutation in *Lotus japonicus* hairy roots by *Glyma.16G033700* or *Lj3g3v3776600*. (a) Schematic representation of the T‐DNA regions that were introduced into *L. japonicus* hairy roots. The expression cassettes for *Lj3g3v3776600* and *Glyma.16G033700* were arranged in tandem with a GFP expression cassette. An empty vector containing only the GFP cassette was used as a negative control. 35S_pro_, CaMV 35S promoter; LB, left border; RB, right border; TNOS, *nopaline synthase* terminator; T35S, CaMV 35S terminator. (b) Representative images of the transgenic hairy roots that were used in the LC‐PDA‐MS/MS analysis. The hairy roots were induced from the hypocotyls of *L. japonicus* plants that were inoculated with *Rhizobium rhizogenes* strains carrying the T‐DNA constructs shown in (a). Bright‐field and GFP fluorescence images of hairy roots transformed with each T‐DNA are shown to confirm successful transformation. (c) LC‐PDA‐MS/MS analysis of hairy root extracts. Hairy roots derived from the homozygous *Lj3g3v3776600* mutant (line 1 in Figure 3) were transformed using either the *35S*_*pro*_ *:Glyma.16G033700* construct, the *35S*_*pro*_ *:Lj3g3v3776600* construct, or an empty vector. Hairy roots from wild‐type plants (ecotype Gifu B‐129) that had been transformed with the empty vector were used as the control. Representative UV chromatograms (292 nm) together with the corresponding MS and MS/MS spectra of soyasaponin βg are shown for *35S*_*pro*_ *:Glyma*.*16G033700*‐transformed mutant roots (mutant line 1) and empty vector‐transformed wild‐type roots.

### Assignment of *Glyma.08G348500* as a C‐22 arabinosyltransferase *in vivo*

As described above, *UGT73K5* is responsible for DDMP conjugation at the C‐22 position. Phylogenetic analysis showed that *Glyma.08G348500* is more closely related to *UGT73K5* than to the previously characterised *UGTs* responsible for the addition of the second or third sugar at the C‐3 position or the second sugar at the C‐22 position of soyasaponins (Figure 5a). In our previous study (Krishnamurthy et al., 2019), we identified three soybean mutants (category 6) that lacked soyasaponins Ab and Ac and accumulated two novel compounds, Y3 and Y4, which are predicted to be Ab‐ and Ac‐like molecules lacking the C‐22 arabinose and acetyl glucose moieties (Figure 5b,c; Figure S5; Table S7). To determine whether these phenotypes were caused by mutations in *Glyma.08G348500*, we sequenced its cDNA in the three mutants. Two lines, EnT‐2743 and EnT‐3537, carried Gly209Ser and Thr380Ile substitutions, respectively (Figure S6a). Co‐segregation analysis confirmed that both mutations were tightly associated with the Y3/Y4 accumulation phenotype (Figure S6b), suggesting that *Glyma.08G348500* is required for the biosynthesis of soyasaponins Ab and Ac.

**Figure 5:**
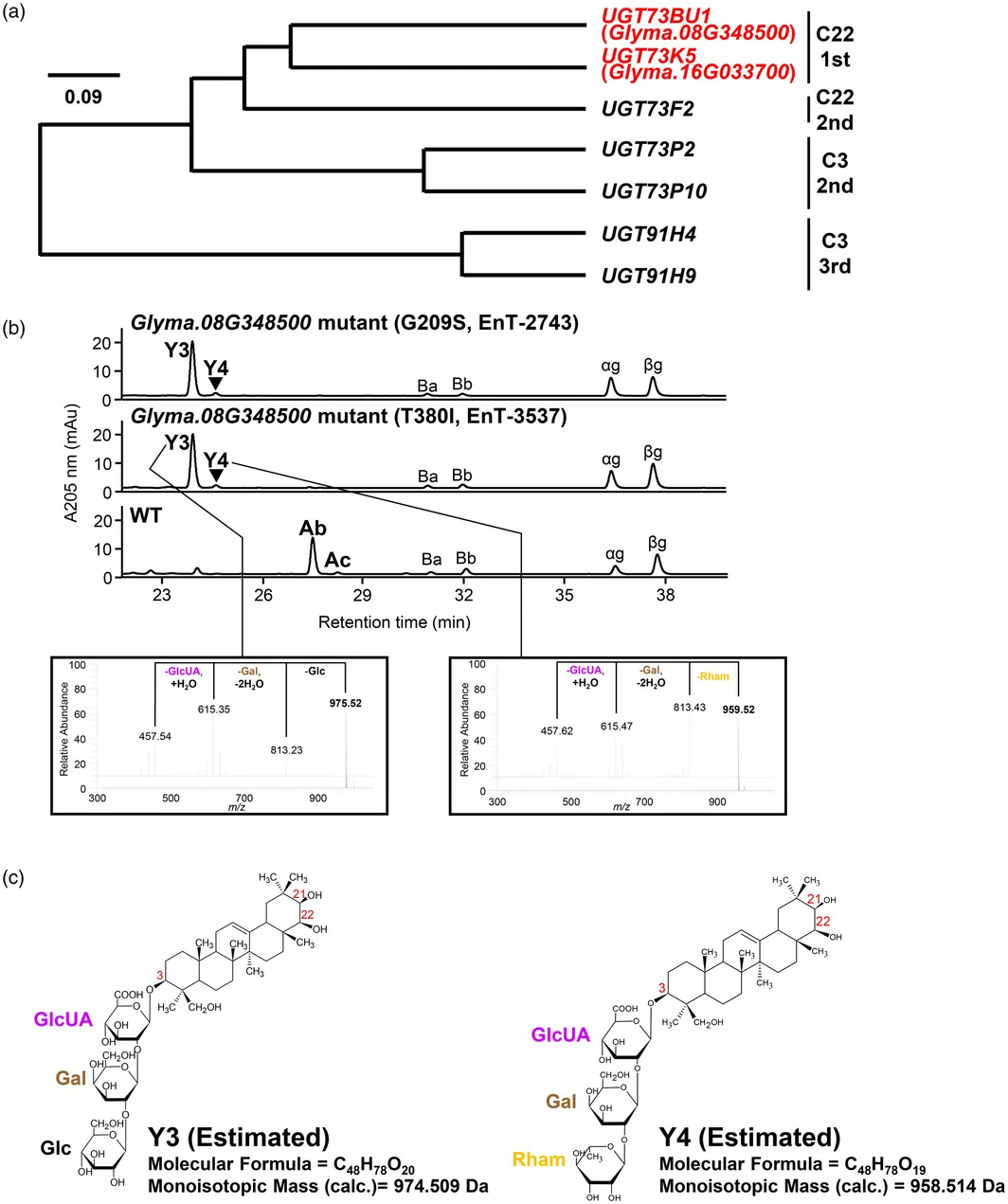
Loss of C‐22 arabinosyltransferase activity in soybean seeds with mutations to *Glyma.08g348500*. (a) Phylogenetic tree of *UDP‐glycosyltransferases* (*UGTs*) involved in soyasaponin biosynthesis. The tree was constructed using predicted full‐length amino acid sequences of *UGTs* that have been characterised for their role in soyasaponin biosynthesis (Figure 1a), alongside *UGT73K5 (Glyma.16G033700*) and *Glyma.08G348500*. The scale bar represents the number of amino acid substitutions per site. The positions of glycosylation on the triterpene skeleton are indicated next to the *UGT* names. (b) LC‐PDA‐MS/MS analysis of seed hypocotyl extracts from two independent *Glyma.08G348500* mutant lines. Peaks corresponding to the known soyasaponins (Ab, Ac, Ba, Bb, αg and βg), as well as the novel compounds Y3 and Y4, are labelled on the UV chromatograms (205 nm). Representative MS and MS/MS spectra for compounds Y3 and Y4 are shown below their respective chromatograms. (c) The predicted structures of compounds Y3 and Y4 based on MS and MS/MS spectra. The colours of the sugar moieties and hydroxylation sites follow the scheme described in Figure 2(b).

We subsequently examined the enzyme activity of Glyma.08G348500 *in vitro* using Y3, purified from EnT‐3537 (Figure S7), as the acceptor and UDP‐arabinose as the donor. Recombinant Glyma.08G348500 protein (Figure S8) expressed in *Escherichia coli* produced a product with an *m/z* value of 1105.6 (corresponding to Y3‐Ara), whereas such a product was not detected with *UGT73K5* or the vector control (Figure 6a). Furthermore, no product was formed when UDP‐glucose, UDP‐galactose, or UDP‐glucuronic acid was used as the sugar donor (Figure S9), demonstrating the strict donor specificity of Glyma.08G348500 for UDP‐arabinose. Together, these genetic and biochemical analyses demonstrate that Glyma.08G348500, hereafter designated UGT73BU1, catalyses arabinose transfer to the C‐22 hydroxyl group of soyasapogenol A‐derived saponins.

**Figure 6:**
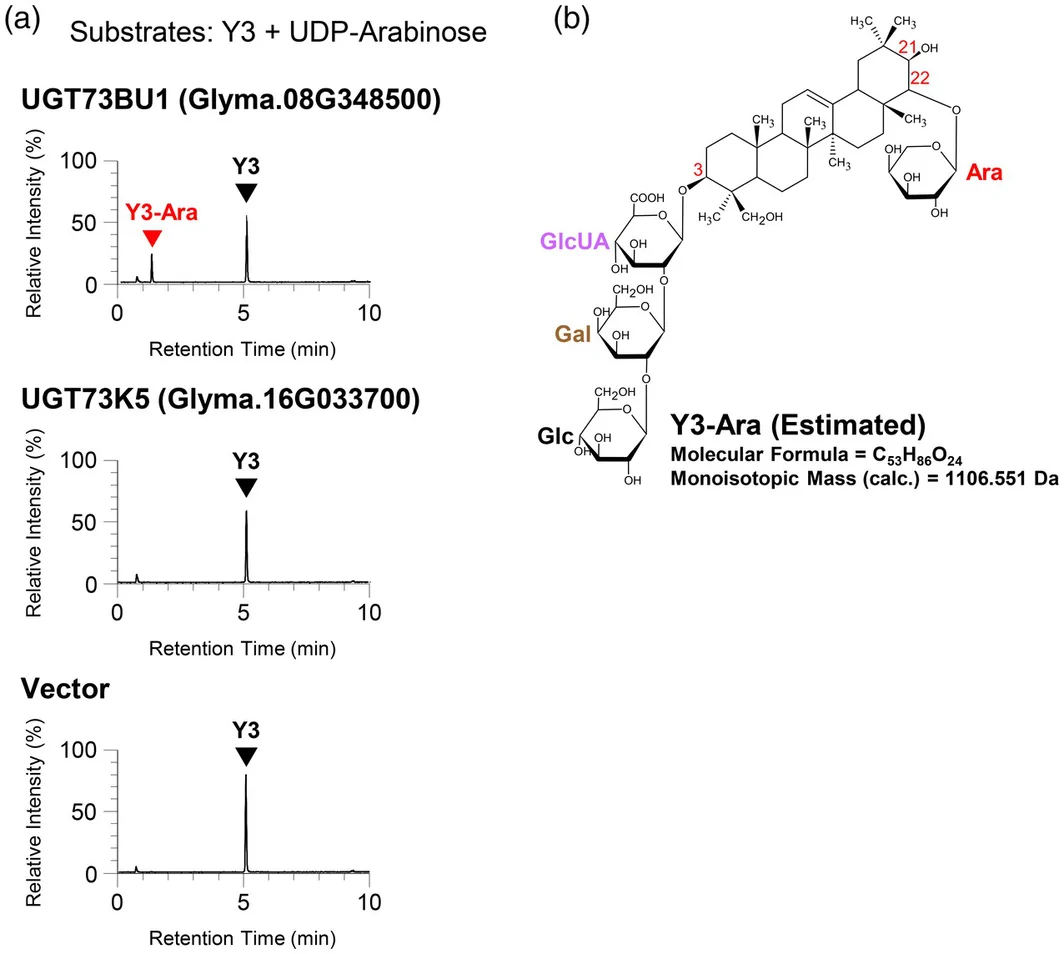
*In vitro* enzymatic activity of UGT73BU1 (Glyma.08G348500). (a) Total ion chromatograms of the reaction products from the *in vitro* enzyme assay, in which compound Y3 (Figure 5c) was used as the sugar acceptor and UDP‐Ara as the sugar donor. The reaction mixtures, which contained either purified UGT73BU1 or UGT73K5 proteins, or the corresponding protein fraction from *Escherichia coli* cells carrying the empty vector (vector), were analysed by LC‐MS. The peaks corresponding to the substrate Y3 (black triangle) and the arabinosylated product Y3‐Ara (red triangle, *m/z* = 1105.6) are indicated. A single protein preparation was used for each UGT, and consistent results were obtained in at least two independent enzyme assays using each preparation. (b) Predicted structure of Y3‐Ara.

### The C‐22 hydroxylase gene is located adjacent to *UGT73K6* in the *L. japonicus* genome

While examining the genomic region surrounding the *UGT73K6* (*Lj3g3v3776600*) in *L. japonicus*, we found a nearby *CYP* gene, *Lj3g3v3776580*, which is located approximately 3.1 kb downstream and separated from *UGT73K6* by a single intervening gene (*Lj3g3v3776590*) (Figure 7a). TBLASTN and BLASTP searches identified *Lj3g3v3776580* as the closest *L. japonicus* homologue of *GmCYP72A61*, the soybean C‐22 hydroxylase. Hereafter, abbreviated species prefixes (e.g., *Gm* and *Lj*) are used for *CYP* gene names, except where the species identity is unambiguous from the context. Phylogenetic analysis placed *Lj3g3v3776580* in the same clade as *GmCYP72A61* (Figure S10), and its expression pattern closely resembled that of the known *L. japonicus* soyasaponin biosynthetic genes *UGT73K6* and *LjCYP93E1* (*Lj1g3v3555800*; Suzuki et al., 2019), and *OSC1* (*Lj3g3v2027430*; Sawai et al., 2006) (Figure S11; Table S8). Given that *GmCYP72A61* catalyses C‐22 hydroxylation of triterpenes (Ebizuka et al., 2011) and that mutations in this gene result in the accumulation of non‐hydroxylated triterpenes (Krishnamurthy et al., 2019), we investigated the function of *Lj3g3v3776580*.

**Figure 7:**
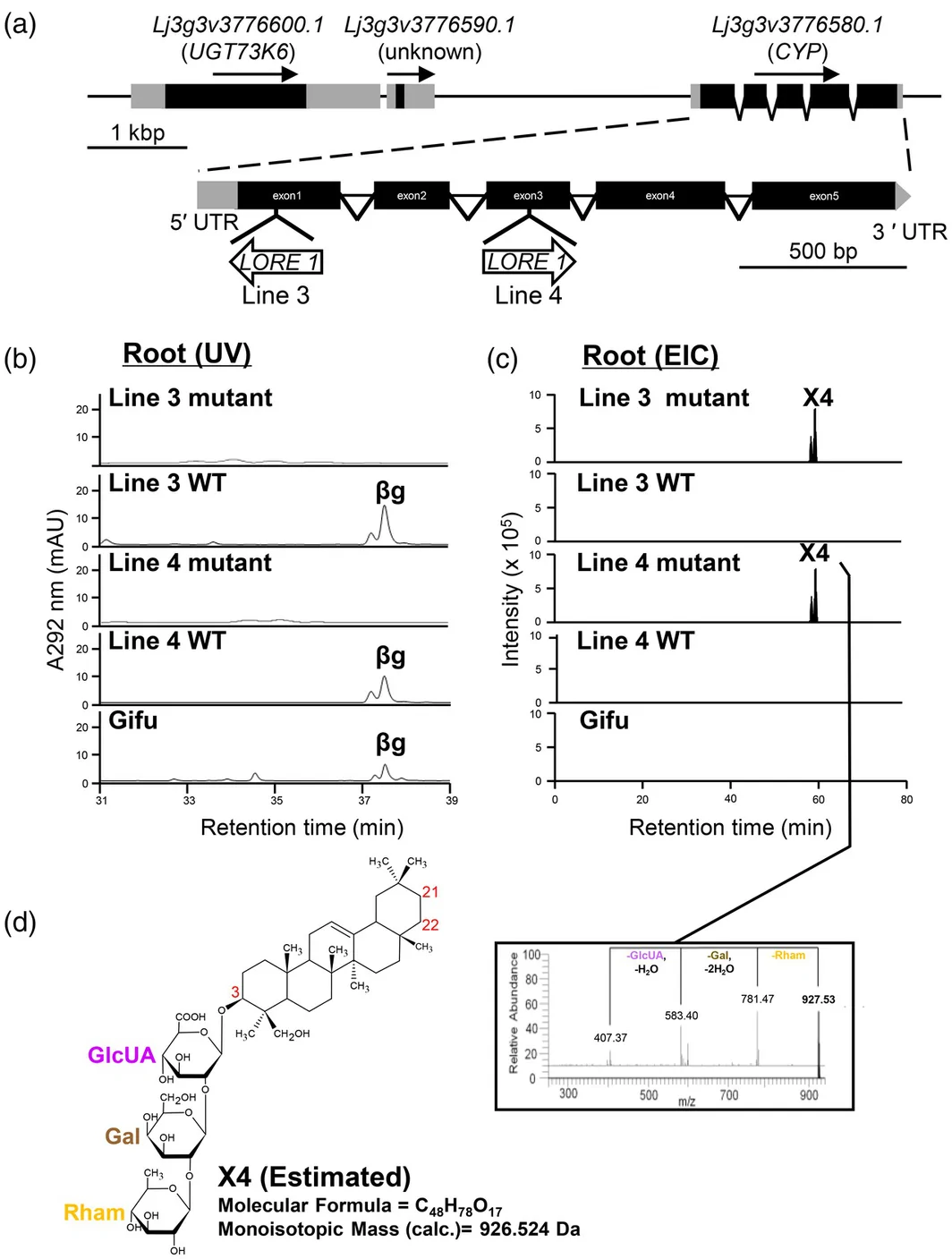
Loss of C‐22 hydroxylase activity in *Lotus japonicus* mutants carrying knockout mutations in a *CYP* gene located adjacent to a *UGT* involved in DDMP conjugation at the C‐22 position. (a) Schematic representation of the genomic region containing the *CYP* gene (*Lj3g3v3776580*). Coding sequences (CDSs), untranslated regions (UTRs), introns and intergenic regions are depicted as black boxes, grey boxes, angled lines and horizontal lines, respectively. Arrows indicate the direction of transcription. An enlarged view of *Lj3g3v3776580* shows the insertion sites of *LORE1* (white arrows) in two independent mutant lines (lines 3 and 4), which correspond to Lotus Base plant IDs 30094065 and 30054528, respectively. These are indicated by inverted Y‐shaped symbols below the exons. (b) LC‐PDA‐MS/MS analysis of root extracts from *Lj3g3v3776580* mutants. UV chromatograms (292 nm) of root extracts from homozygous *LORE1* insertion mutants (mutants) and their homozygous wild‐type siblings (WT) from segregating populations are shown. Peaks corresponding to soyasaponin βg are indicated. (c) Extracted ion chromatograms (EICs; *m/z* = 927.53) of the same samples as in (b). Compound X4, which accumulated in the *Lj3g3v3776580* mutant but was absent in the wild‐type Gifu B‐129 roots, is indicated. Representative MS and MS/MS spectra of X4 from mutant line 4 are shown below the chromatograms. (d) Predicted structure of compound X4 based on MS and MS/MS spectra.

Two independent *LORE1* insertion mutants of *Lj3g3v3776580* were obtained. Soyasaponin βg was absent from the roots of homozygous mutants (lines 3 and 4; Figure 7a; Table S6) but was detected in the wild‐type siblings (Figure 7b). Instead, the mutants accumulated a novel compound designated X4 (*m/z* = 927.53) which eluted later than soyasaponin Bb (*m/z* = 943.53). Soyasaponin Bb is a compound derived from βg by removal of the DDMP moiety, and X4 was 16 mass smaller than Bb (Figure 7c,d). Taken together, X4 is predicted to be a soyasaponin Bb‐like compound lacking the C‐22 hydroxyl group, indicating the loss of C‐22 hydroxylase activity in the *Lj3g3v3776580* mutants. To verify its enzymatic function, *Lj3g3v3776580* or *GmCYP72A61* was co‐expressed with cytochrome P450 reductase from *L. japonicus* (*LjCPR1*), which serves as an electron donor, in a triterpene‐producing *Saccharomyces cerevisiae* strain. Expression of either *CYP* resulted in the production of the C‐22 hydroxylated triterpenes sophoradiol and soyasapogenol B, demonstrating that Lj3g3v3776580 possesses the same C‐22 hydroxylase activity as GmCYP72A61 (Figure S12). We next performed genetic complementation using transgenic hairy roots. Expression of either *Lj3g3v3776580* or *GmCYP72A61* restored βg accumulation and eliminated X4 in the mutant background (Figure S13b,c). MS/MS analysis confirmed the identity of the recovered βg peak. These genetic and biochemical analyses demonstrate that *Lj3g3v3776580* encodes a C‐22 hydroxylase that is functionally equivalent to GmCYP72A61. Accordingly, *Lj3g3v3776580* was designated *LjCYP72A61*.

### Microsynteny of legume genomic regions containing *UGT* and *CYP* genes involved in DDMP saponin biosynthesis

As described above, the two genes responsible for C‐22 modification, *CYP72A61* and *UGT73K6*, are located within an approximately 8 kb region in the *L. japonicus* genome, forming a gene cluster (Figure 7a). *Medicago truncatula* also possesses genes homologous to the soybean *CYP72A61* and *UGT73K5*, which are designated as *CYP72A61v2* and *UGT73K1*, respectively. These genes also form a similar gene cluster (see Figure 7a and Figure S14a). To investigate the conservation of genomic regions containing C‐22 modification genes, we compared five legume species known to produce DDMP saponins: soybean (Kudou et al., 1993), *L. japonicus* (Tsuno et al., 2018), *P. vulgaris* (Bianco et al., 2018), adzuki bean (*Vigna angularis*) (Iida et al., 1997, 1999) and *M. truncatula*, which also accumulates a predicted DDMP saponin (Figure S15).

A gene‐based synteny analysis which was conducted using reciprocal BLASTN and BLASTP searches revealed conservation between the *CYP72A61–UGT73K6* region on *L. japonicus* chromosome 3 and the *CYP72A61v2–UGT73K1* region on *M. truncatula* chromosome 4 (Figure 8a). Additional syntenic regions spanning 135–215 kb and containing 13–20 homologous gene pairs were identified on chromosome 8 of soybean, chromosome 11 of *P. vulgaris*, and chromosome 6 of *V. angularis*. However, unlike those in *L. japonicus* and *M. truncatula*, these syntenic regions contained only *CYP72A61* homologues and lacked adjacent *UGT73K*‐family genes. Although no *CYP72A61* homologue was identified within the syntenic region of chromosome 6 in the *V. angularis* genome, a closely related homologue (91% amino acid sequence identity to GmCYP72A61) was identified on chromosome 9. This region on chromosome 9 exhibited no detectable microsynteny with the soybean genome. In soybean, *UGT73K5* is located on chromosome 16, and its homologues are found on chromosome 10 of *P. vulgaris* and chromosome 9 of *V. angularis*. These regions also exhibited conserved microsynteny (Figure 8b), indicating that the *CYP72A61* and *UGT73K* loci have followed distinct evolutionary trajectories in different legume lineages.

**Figure 8:**
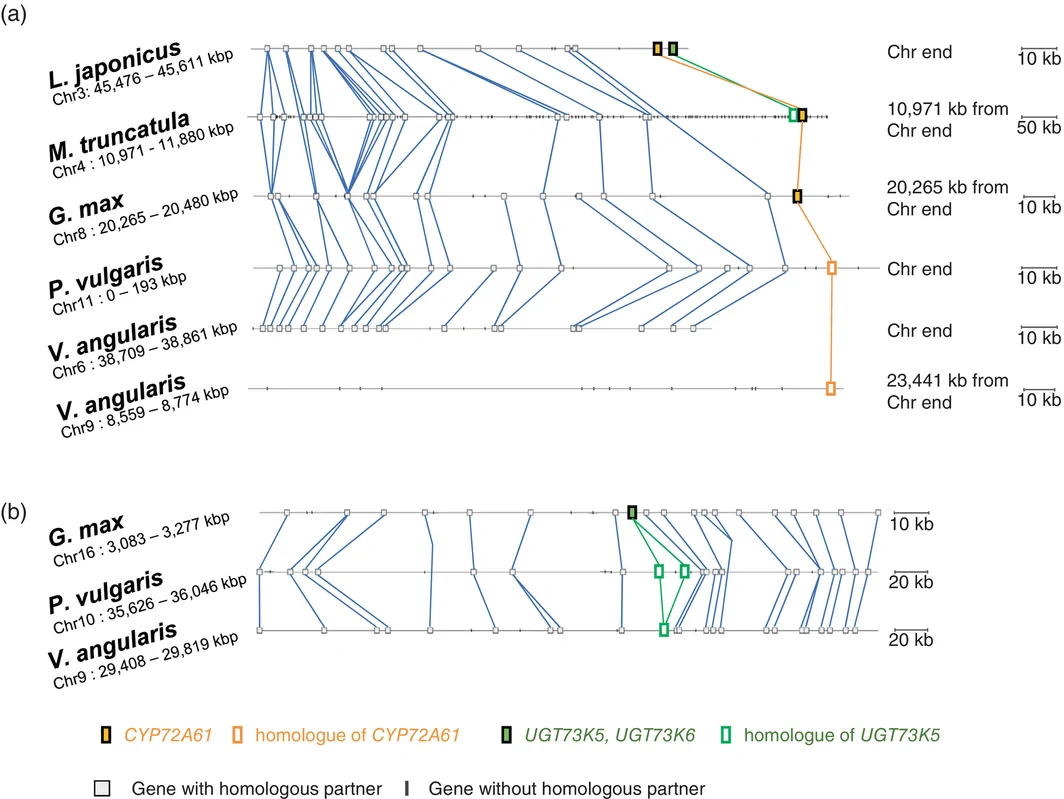
Microsynteny of genomic regions containing genes involved in the biosynthesis of DDMP saponins in legume species. (a) Microsynteny in the vicinity of *LjCYP72A61* and its homologues. In both *Lotus japonicus* and *Medicago truncatula*, *LjCYP72A61* and its homologue *MtCYP72A61v2* are located adjacent to *UGT73K6* and *UGT73K1*, respectively. Genes with homologous counterparts are shown as boxes, while genes without detectable homologues are shown as short vertical lines. Genes involved in DDMP saponin biosynthesis and their homologues are highlighted in orange (*CYP72A61* family) or green (*UGT73K5* family). Homologous genes are connected by coloured lines. Genomic regions are drawn at different scales to display the complete syntenic regions, and the scale bar for each region is shown on the right. Chromosome numbers and approximate chromosomal positions are indicated below the abbreviated Latin species names. A legend for the gene symbols is provided at the bottom of the figure. (b) Microsynteny in genomic regions surrounding the *UGT73K5* gene, which is involved in DDMP conjugation at the C‐22 position, and its homologues.

## DISCUSSION

The biosynthesis of saponins and other specialised metabolites is tightly regulated both temporally and spatially, and genes involved in these pathways often exhibit coordinated expression patterns (Field & Osbourn, 2008; Fukushima et al., 2013; Huang et al., 2019; Krokida et al., 2013; Polturak et al., 2021, 2022; Zhou et al., 2016). Therefore, co‐expression analysis is a reliable approach for identifying previously uncharacterised genes involved in these pathways, and numerous biosynthetic enzymes have been successfully discovered using this approach (Chung et al., 2020; Huang et al., 2019; Jozwiak et al., 2020; Polturak et al., 2022). Soybean produces two major classes of soyasaponins that are distinguished by chemical modifications at the C‐21 and C‐22 positions of the triterpene skeleton. In particular, the C‐22 substituent distinguishes DDMP saponins from group A saponins. In this study, we focused on identifying the enzymes responsible for C‐22 modifications. Co‐expression analysis using known soyasaponin biosynthetic genes identified *UGT73K5* as a candidate enzyme gene for DDMP conjugation at the C‐22 position in soybean (Figure 1b). Functional validation using loss‐of‐function soybean mutants demonstrated that *UGT73K5* is essential for DDMP saponin accumulation in seeds (Figure 2a). Functional conservation of this activity was further demonstrated in *L. japonicus*, in which the orthologue *UGT73K6* was required for DDMP saponin biosynthesis and was functionally complemented by soybean *UGT73K5* (Figures 3 and 4). One limitation of this study is that the enzymatic activity of UGT73K5 and UGT73K6 could not be demonstrated directly *in vitro* because neither UDP‐DDMP nor an appropriate UDP sugar precursor was available as a sugar donor. Future studies using chemically synthesised UDP‐DDMP or suitable precursors should enable direct biochemical characterisation of these enzymes.

DDMP saponins accumulate in multiple organs in both soybean and *L. japonicus* (Tsuno et al., 2018). In both species, *CYP72A61* and either *UGT73K5* or *UGT73K6* are co‐expressed with other core soyasaponin biosynthetic genes, including *bAS* (β‐amyrin synthase), *CSyGT* (C‐3 glucuronosyltransferase) and *CYP93E1* (C‐24 oxidase) (Figure 1B; Figures S1 and S11; Table S10). These observations suggest that these genes constitute a conserved transcriptional module underlying soyasaponin biosynthesis in legumes. In group A saponins, the C‐22 position of the triterpene skeleton is modified with an arabinose moiety. Although two additional putative *UGT* genes were identified through co‐expression analysis in this study, soyasaponin Ab, the major group A saponin, still normally accumulated in the seed hypocotyls of soybean lines carrying frameshift or nonsense mutations in these genes (Figure 2a). This result suggested that C‐22 arabinosylation is catalysed by another UGT. Plant *UGTs* constitute a large gene family; for example, the soybean genome contains over 200 *UGT* genes (Yin et al., 2017), making it challenging to identify the enzyme responsible for a specific biosynthetic reaction. Previous studies have shown that phylogenetically related UGTs tend to accept structurally similar substrates (Louveau et al., 2018; Ross et al., 2001) and often catalyse glycosylation at the same hydroxyl positions on the aglycon (Lim et al., 2003). We therefore searched for UGTs closely related to UGT73K5, which is the enzyme responsible for DDMP conjugation at the C‐22 position. Phylogenetic analysis identified *UGT73BU1* as the closest homologue of *UGT73K5*, with higher sequence similarity than *UGTs* involved in the addition of the second or third sugar at the C‐3 position or of the second sugar at the C‐22 position (Figure 5a). Functional analysis of *ugt73bu1* mutant seeds (Figure 5b) together with an *in vitro* enzyme assay using recombinant UGT73BU1 (Figure 6) demonstrated that UGT73BU1 catalyses the transfer of arabinose to the C‐22 hydroxyl group of soyasapogenol A‐derived saponins.

In the model legume *M*. *truncatula*, two classes of saponins are produced: haemolytic and non‐haemolytic saponins, which are distinguished by C‐18 carboxylation and C‐22 hydroxylation, respectively (Fukushima et al., 2013; Mertens et al., 2016). Their biosynthetic pathways diverge after β‐amyrin formation. In the haemolytic branch, oxidation of the triterpene skeleton is catalysed by MtCYP716A12, MtCYP72A68v2 and MtCYP72A67, whereas MtCYP93E2 and MtCYP72A61v2 are involved in the non‐haemolytic saponin biosynthetic pathway. These two pathways are controlled by distinct basic helix–loop–helix transcription factors, with TSAR2 regulating the haemolytic pathway and TSAR1 controlling the non‐haemolytic pathway (Mertens et al., 2016). In soybean, genes specific to group A saponin biosynthesis, including *UGT73BU1* (C‐22 arabinosyltransferase) and *CYP72A69* (C‐21 hydroxylase), were not included in the co‐expression network (Figure 1b). Moreover, *CYP72A69* is expressed predominantly in hypocotyls, whereas *CYP72A61* and *GmSGT2* (*UGT73P2*), which participate in the biosynthesis of both DDMP and group A soyasaponins, are expressed at comparable levels in hypocotyls and cotyledons (Yano et al., 2017). Consistent with these expression patterns, DDMP saponins accumulate in both hypocotyls and cotyledons, whereas group A saponins are confined to the hypocotyls (Krishnamurthy et al., 2019; Tsuno et al., 2018). These observations suggest that, as in *M. truncatula*, the DDMP and group A saponin pathways are likely to be regulated by distinct transcriptional mechanisms after branching from the common biosynthetic pathway. Future identification and functional characterisation of the transcription factors governing soyasaponin biosynthesis will be essential for testing this hypothesis.

In various plant species, genes encoding enzymes for the biosynthesis of saponins and other specialised metabolites are frequently organised into gene clusters (Field & Osbourn, 2008; Huang et al., 2019; Itkin et al., 2013; Krokida et al., 2013; Polturak et al., 2021, 2022; Zhou et al., 2016). Some of these clusters also include genes encoding transcription factors and metabolite transporters (Ma et al., 2023; Zhou et al., 2016). Although the biological and evolutionary significance of such clustering is not fully understood, these gene clusters are thought to be maintained under strong selective pressure and to facilitate adaptation to changing environments (Polturak et al., 2021). In the *L. japonicus* genome, we identified *LjCYP72A61*, a *CYP* gene that is located adjacent to *UGT73K6* and catalyses C‐22 hydroxylation preceding DDMP conjugation (Figure S14). Functional analyses using *LORE1* mutants (Figure 7), genetic complementation (Figure S13) and heterologous expression in yeast (Figure S12) demonstrated that *LjCYP72A61* functions as a C‐22 hydroxylase. Thus, *LjCYP72A61* and *UGT73K6* form a gene cluster that catalyses consecutive steps in DDMP saponin biosynthesis. A similar genomic organisation is found in *M. truncatula*, where the homologous genes *MtCYP72A61v2* and *UGT73K1* are located adjacent to one another (Figure S14) and are co‐expressed (Fukushima et al., 2013; Figure S11; Table S10). In *L. japonicus*, both LjCYP72A61 and UGT73K6 localise to the endoplasmic reticulum (Figure S17), supporting their coordinated function. Although the function of *UGT73K1* has not been validated *in vivo*, recombinant UGT73K1 transfers glucose to multiple hydroxyl positions, including C‐22, *in vitro* (Achnine et al., 2005). In the soybean genome, *UGT73K5* and *GmCYP72A61* are located on different chromosomes (Figure 8), which may reflect genomic rearrangements that occurred during the divergence of different legume lineages. Nevertheless, the two genes are co‐expressed (see Figure 1b), suggesting that the coordinated transcriptional regulation of C‐22 hydroxylation and DDMP conjugation has been conserved across soybean, *L. japonicus*, and *M. truncatula*, despite their different genomic organisations.

Comparative analysis of genomic regions containing genes involved in DDMP saponin biosynthesis across five legume species revealed extensive microsynteny. Conserved synteny was observed among the genomic regions adjacent to the *CYP72A61–UGT73K6* cluster in *L. japonicus* (chromosome 3), the *CYP72A61v2–UGT73K1* cluster in *M. truncatula* (chromosome 4), the *CYP72A61* gene in soybean (chromosome 8) and a *CYP72A61* homologue in *P. vulgaris* (chromosome 11) (Figure 8a), indicating that these genomic regions are derived from a common ancestral locus. By contrast, the *CYP72A61* homologue in *V. angularis* was located on chromosome 9 in a region lacking detectable synteny with the corresponding regions in the other legumes, suggesting that the genomic organisation of this locus has undergone a distinct evolutionary history in adzuki bean. In soybean, *P. vulgaris*, and *V. angularis*, *CYP72A61* and *UGT73K5* homologues are located on different chromosomes rather than forming a gene cluster. *UGT73K5* and its homologues in *P. vulgaris* and *V. angularis* are located within syntenic genomic regions on chromosomes 16 of soybean, chromosome 10 of *P. vulgaris*, and chromosome 9 of *V. angularis* (Figure 8b), supporting a common evolutionary origin for these loci. Most agriculturally important legumes belong to the subfamily Papilionoideae, which is divided into several clades. Soybean, *V. angularis* and *P. vulgaris* are classified into the Millettioid clade, whereas *L. japonicus* and *M. truncatula* belong to the Hologalegina clade (Figure S18; Cannon, 2013; Choi et al., 2022; Koenen et al., 2021; Lavin et al., 2005). Although it is thought that these lineages diverged more than 50 million years ago (Lavin et al., 2005), DDMP saponins are widely distributed throughout the Papilionoideae (see Figure S18; Bianco et al., 2018; Iida et al., 1999; Karolkowski et al., 2023; Kudou et al., 1993; Ruiz et al., 1995, 1996; Tsuno et al., 2018; Vernoud et al., 2021), which suggests that the core biosynthetic machinery required for the production of DDMP saponins had already evolved in their common ancestor. Consistent with this hypothesis, *CYP72A61*, *UGT73K5* and their homologues are located on the same chromosome in *L. japonicus*, *Glycyrrhiza uralensis*, *Cicer arietinum*, *M. truncatula*, *Trifolium alexandrinum*, *Lens culinaris*, *Vicia faba*, *Pisum sativum* and *V. angularis*. In contrast, these genes are located on different chromosomes in *Arachis hypogaea*, *Lupinus albus*, *Cajanus cajan*, *P. vulgaris* and soybean (Figure S18; Table S11). Interestingly, species in which *CYP72A61* and *UGT73K5* remain linked are confined to the Hologalegina clade. In contrast, species in which the two genes are separated belong to the Millettioid and other clades (see Figure S18 and Cardoso et al., 2013). These observations suggest that the genomic separation of *CYP72A61* and *UGT73K5* homologues occurred during the evolution of the Millettioid lineage, following the divergence of the major papilionoid clades.

In various plant species, saponins have been implicated in diverse biological processes, including resistance to pathogenic fungi (Leveau et al., 2019; Louveau et al., 2018; Papadopoulou et al., 1999) and pests (Kuzina et al., 2009; Tava & Odoardi, 1996), allelopathy (Leshem & Levin, 1978; Miller, 1983; Oleszek & Jurzysta, 1987; Waller et al., 1999) and nodulation (Confalonieri et al., 2009). However, these functions have largely been inferred without the use of mutants carrying defined alterations in endogenous saponin composition or abundance. Through our continuing efforts to elucidate the soyasaponin biosynthetic pathway in soybean (Krishnamurthy et al., 2019; Takagi et al., 2018; Yano et al., 2017, 2018), we have generated a large collection of mutant lines with diverse soyasaponin profiles. Together with the biosynthetic genes identified in this study, these genetic resources provide a valuable platform for elucidating the physiological functions of soyasaponins in plants, determining their contributions to the quality of soybean‐derived foods, and evaluating their potential health benefits for humans.

## MATERIALS AND METHODS

### Plant materials

All the soybean mutants used in this study were derived from high‐density mutant libraries induced by EMS and generated in the cultivar Enrei, as previously described by Tsuda et al. (2015). In brief, EMS treatment was first applied to Enrei seeds to produce the M1 generation. Following self‐pollination, the M2 seeds were grouped together and subjected to a second round of EMS mutagenesis to produce the M1′ generation. Self‐pollinating the resulting M1′ plants produced M2′ populations in which an average of 691 missense and 35 nonsense mutations were introduced per line. Three independent M2′ populations cultivated in 2012, 2013 and 2014 were used in this study. For reverse genetic screening, high‐resolution melting analysis was performed using pooled genomic DNA samples from leaves of M2′ plants (1536 lines per library), as described by Tsuda et al. (2015). Saponin composition was analysed in seeds from the M3′ generation or later. Primer sequences used for mutant screening are listed in Table S12. The *Glyma.18G208300* mutant was identified by amplicon sequencing using a next‐generation sequencing platform, following the method described by Tsuda et al. (2015). All soybean plants were grown in either the experimental field or greenhouse at the Institute of Crop Science, National Agriculture and Food Research Organisation (36°01′ N, 140°06′ E).

*Lotus japonicus* mutants were obtained from the *Lotus retrotransposon 1* (*LORE1*) insertion mutant resource (Małolepszy et al., 2016) *via* Lotus Base (https://lotus.au.dk/; Mun et al., 2016). Of the three independent *Lj3g3v3776600* (*UGT73K6*) mutant lines available on Lotus Base, two carry either four or five *LORE1* insertions (lines 1 and 2 in Figure 3a, corresponding to LOTUS BASE plant IDs 30058798 and A01254, respectively, see Table S6). Three independent mutant lines were identified for *Lj3g3v3776580* (*CYP72A61*). Two of these lines were selected for further analysis: line 3 (Lotus Base plant ID 30094065), which carries four *LORE1* insertions; and line 4 (Lotus Base plant ID 30054528), which carries one *LORE1* insertion (Figure 7A; Table S6). *L. japonicus* plants were grown in growth chambers at 23°C under a 16‐h light/8‐h dark photocycle or in a greenhouse. Genomic DNA was extracted from cotyledons after seed germination and the presence of *LORE1* insertion was confirmed by PCR using the primers listed in Table S12.

### Co‐expression analysis

Co‐expression analysis was conducted using the ‘Option III: Find functional modules’ in SoyNet (Kim et al., 2017). The guide genes used for the analysis were previously characterised genes encoding enzymes involved in soyasaponin biosynthesis (Figure 1a). The Phytozome gene identifiers (ID) for these guide genes are listed in Table S1. The resulting soybean co‐expression network was visualised using Cytoscape software (version 3.7.2, Shannon et al., 2003). The expression profiles of the guide genes and the intermediate genes, representing candidate genes for the previously uncharacterised components of the co‐expressed module, were visualised as heatmaps based on transcripts per million (TPM) values retrieved from the Phytozome v14 database (Sreedasyam et al., 2023). For *L. japonicus*, gene expression data were visualised as heatmaps based on Affymetrix GeneChip data obtained from the *L. japonicus* Gene Expression Atlas (Verdier et al., 2013) available in Lotus Base.

### Analysis of saponin composition

Dry soybean seeds were separated into hypocotyls and cotyledons. The cotyledons were ground into a fine powder using a Multibeads Shocker (Yasui Kikai, Osaka, Japan). Saponins were extracted from intact hypocotyls and powdered cotyledons using 80% (v.v) aqueous methanol for 24 h (hypocotyls) or 1 h (cotyledons) with gentle agitation at room temperature (approximately 25°C). Roots of *L. japonicus* were harvested 1 month after germination, freeze‐dried and ground into a fine powder using a Multibeads Shocker (Yasui Kikai). Saponins were extracted with 80% (v.v) methanol by gentle agitation for 1 h at room temperature. After centrifugation of the homogenates at 20 000*×**g*** for 5 min, the supernatant was filtered through a GL‐Chromatodisc 4P filter (0.45 μm pore size, 4 mm diameter; GL Science, Tokyo, Japan). Unfiltered hypocotyl extract and the filtrates of soybean cotyledon or *L. japonicus* root extracts were analysed by LC‐PDA‐MS/MS using an Ultimate 3000 SD HPLC system coupled with a LTQ Orbitrap Discovery mass spectrometer (Thermo Fisher Scientific, MA, USA) according to previously published procedures (Krishnamurthy et al., 2019) with minor modifications. The LTQ Orbitrap MS was operated in electrospray ionisation (ESI) positive ion mode with the following parameters: a capillary temperature of 275°C, a sheath gas flow rate of 5 (arbitrary unit), an auxiliary gas flow rate of 6 (arbitrary unit), a sweep gas flow rate of 0 (arbitrary unit), a source voltage of 4.5 kV, a capillary voltage of 49 V, and a tube lens voltage of 80 V. Full‐scan mass spectra (*m/z* 100–1500) was acquired in Fourier transform (FT) detector mode at a resolution of 30 000. The MS/MS spectra were acquired by data‐dependent acquisition in FT detector mode at a resolution of 30 000. The instrument was calibrated daily using Pierce LTQ ESI Positive Ion Calibration Solution (Thermo Fisher Scientific), covering the *m/z* range of 150–2000. Saponins were detected as protonated ions ([M + H]^+^) with a mass tolerance of 5 ppm. Xcalibur 2.1 software (Thermo Fisher Scientific) was used for instrument control, data acquisition and data processing.

For acid hydrolysis, a 44 μl aliquot of crude extract was mixed with 4 μl of 12 N HCl and incubated at 85°C for 3 h before LC‐PDA‐MS analysis. TLC was performed according to the method described by Krishnamurthy et al. (2019).

Authentic reference standards were available only for soyasaponin Bb (Figure S5). Therefore, the structural assignments of other soyasaponins should be regarded as tentative. These assignments were based on retention times and MS/MS fragmentation patterns and were consistent with those reported previously (Krishnamurthy et al., 2019; Sayama et al., 2012; Takagi et al., 2011, 2018; Yano et al., 2017, 2018). Although authentic standards were not available for these compounds, the agreement of their chromatographic and MS/MS characteristics with previous reports supports the reliability of the structural assignments.

All analyses were performed using at least three independent soybean seeds or three independent *L. japonicus* plants for each genotype, and reproducible results were obtained.

### Functional complementation of *L. japonicus* mutants

Total RNA was extracted from developing soybean (cultivar Enrei) seeds and *L. japonicus* (ecotype Gifu B‐129) roots, both grown in greenhouses, using a RNeasy Plant Mini Kit (QIAGEN, Hilden, Germany). First‐strand cDNA was synthesised using a QuantiTect Reverse Transcription Kit (QIAGEN). The cDNAs of *UGT73K5*, *GmCYP72A61*, *UGT73K6* and *LjCYP72A61* were then amplified from the first‐strand cDNAs by PCR using PrimeSTAR GXL DNA Polymerase (Takara Bio Inc., Shiga, Japan). These cDNAs were cloned into the pDONR 221 vector (Thermo Fisher Scientific) and sequenced to confirm the absence of PCR‐induced errors. *Rhizobium rhizogenes*‐mediated hairy root transformation was performed as described by Chung et al. (2020). The primers used for cDNA amplification are listed in Table S12. We used hairy roots derived from at least two independent transformation events for each construct. For each construct, plants exhibiting the strongest GFP fluorescence, which served as a marker for transgene expression, were selected for analysis. Consistent results were obtained from the independent transformation events.

### Phylogenetic analysis

The amino acid sequences of the UGTs and CYPs from the soybean cultivar Williams 82 (Gmax_508_Wm82.a4.v1) were obtained from the Phytozome database (https://phytozome‐next.jgi.doe.gov/). The Phytozome gene IDs are provided in Table S1. The predicted amino acid sequences of the UGTs and CYPs from *L. japonicus* (ecotype Miyakojima MG‐20) were obtained from Lotus Base (https://lotus.au.dk/). The Lotus Base gene IDs are listed in Table S5 (*UGTs*) and Figure S10 (*CYPs*). The predicted amino acid sequences of CYPs from *V. angularis* (cultivar Longxiaodo No. 4, assembly ASM1680809v1) (Li et al., 2024) were retrieved from GenBank (https://www.ncbi.nlm.nih.gov/datasets/genome/GCF_016808095.1/). The accession numbers of the *V. angularis* sequences are listed in Tables S9 and S11. All phylogenetic trees were constructed using the unweighted pair group method with arithmetic mean (UPGMA) implemented in Geneious Prime (version 2020.0.5).

### *In vitro* enzyme assay

UDP‐Ara was purchased from CarboSource Services (Athens, GA, USA) and used as the sugar donor. The sugar acceptor (compound Y3) was purified from the seeds of the EnT‐3537 mutant (see Figure 5B; Figure S6). Seed hypocotyls (10 g) were immersed in 100 ml of 80% (v.v) methanol with gentle agitation at room temperature for 24 h. HPLC separations were performed using a reverse‐phase C18 column (Unison C18‐US‐3; 20 mm inner diameter × 250 mm; Imtakt, Kyoto, Japan) at 40°C. The extract was injected into the column at a flow rate of 1.0 ml min^−1^ and then separation was performed under isocratic conditions of 36% (v.v) aqueous acetonitrile supplemented with 0.1% (v.v) acetic acid at a flow rate of 0.5 ml min^−1^. Fractions (3 ml each) were collected and concentrated under vacuum using a rotary evaporator at 65°C. Fractions containing compound Y3 (eluting at 32 min) were collected, freeze‐dried and resuspended in 80% methanol. The purity of Y3 was confirmed by LC‐PDA‐MS/MS (see Figure S7).

The *UGT73K5* and *UGT73BU1* cDNAs were amplified by PCR using PrimeSTAR GXL DNA Polymerase (TaKaRa Bio Inc.) from the first‐strand cDNA described above and were then cloned into pDONR221 and pENTR/D‐TOPO vectors (Thermo Fisher Scientific), respectively. Sequencing was performed to confirm the absence of PCR‐induced errors. The recombinant proteins were expressed as fusion proteins with trigger factor in *E. coli*, and their activities were assayed as described by Nomura et al. (2019). The primers used for cDNA amplification are listed in Table S12.

### *In vivo* enzyme assay

*LjCYP72A61* cDNA was transferred to the pYES‐DEST52 vector (Thermo Fisher Scientific) to generate the galactose‐inducible expression construct pDEST52‐*LjCYP72A61* construct. This construct was introduced into an *S. cerevisiae* strain previously transformed with the pYES3‐*ADH‐OSC1* plasmid, which constitutively expresses the *L. japonicus* β‐amyrin synthase (*LjbAS*) gene under the control of the *ADH1* promoter (Suzuki et al., 2018), and with the pELC‐*LjCYP93E1* plasmid, which enables galactose‐inducible co‐expression of *LjCYP93E1* and the *L. japonicus cytochrome P450 reductase* (*LjCPR1*) genes (Suzuki et al., 2019). Following galactose induction of *CYPs* and *CPR*, the reaction products were extracted and analysed using GC–MS. Details of the yeast strains and experimental procedures are described in Suzuki et al. (2019).

### Subcellular localisation of UGT73K6 and CYP72A61 in *L. Japonicus* hairy roots

The stop‐codon–less cDNAs of *UGT73K6* and *LjCYP72A61* were amplified by PCR using the primers listed in Table S12. The cDNAs were then cloned into pDONR221 and sequenced to confirm the absence of PCR‐induced errors. The stop‐codon‐less cDNAs of *UGT73K6* and *LjCYP72A61* were subsequently transferred into pK7RWG2 (Karimi et al., 2002) to generate in‐frame fusions with the RFP coding sequence. Hairy root transformation and confocal microscopy were performed as described by Chung et al. (2020).

### Microsynteny analysis

The coding sequences of *UGT73K5*, *GmCYP72A61* and their homologues (encoding proteins of more than 400 amino acids) were identified in the genomes of soybean (*G. max*; Glyma2.0) (Schmutz et al., 2010), common bean (*P. vulgaris*; v1.0 release) (Schmutz et al., 2014) and barrel medic (*M*. *truncatula*) (Young et al., 2011). These sequences are all available in the Phytozome database (http://www.phytozome.net/). *UGT73K6* and *CYP72A61* were identified in the *L. japonicus* genome (Sato et al., 2008) using Lotus Base. A *UGT73K5* homologue was identified in the *V. angularis* (cultivar Shumari) genome (Sakai et al., 2015) using the VIGNA GENOME SERVER (https://viggs.dna.affrc.go.jp/; Sakai et al., 2016). A *CYP72A61* homologue of *V. angularis* (cultivar Longxiaodo No. 4) was retrieved as described in the phylogenetic analysis section. To detect interspecies orthologous relationships, genome‐wide reciprocal BLASTN and BLASTP analyses were performed using BLAST version 2.2.29+ (Camacho et al., 2009). Gene‐level microsynteny was defined as the presence of at least three best‐matching homologous genes within a window of 10 consecutive genes (Yano et al., 2017).

## ACCESSION NUMBERS

cDNA sequences were submitted to GenBank with accession numbers: LC433765 (UGT73K5 from soybean cultivar Enrei), LC489234 (UGT73BU1 from soybean cultivar Enrei) and LC433638 (UGT73K6 from Lotus japonicus Gifu B‐129).

## AUTHOR CONTRIBUTIONS

MI and TM designed the research; SH, YF, PK, H‐RS, SYC, HaS, RN, YS, RY, AK, CT and HiS performed the research; SH performed the co‐expression and phylogenetic analyses; YF, PK and AK screened the soybean mutants; HRS, RN and CT analysed the saponin profiles of soybean and *L. japonicus* and purified Y3; YF and YS conducted *L. japonicus* hairy root transformation and confocal microscopy; SYC, HaS and HiS performed the *in vitro* and *in vivo* enzyme assays; RY conducted the microsynteny analysis; SH and MI wrote the manuscript.

## CONFLICT OF INTEREST

The authors claim no conflicts of interest.

## Supporting information


**Table S1.** List of guide and intermediate genes in co‐expression analysis.
**Table S2.** List of *Glyma.16G033700* mutants obtained in this study.
**Table S3.** List of *Glyma.15G221300* mutants obtained in this study.
**Table S4.** Result of *Glyma.18G208300* mutant screening by amplicon sequencing.
**Table S5.** Amino acid sequence identities among soybean and *Lotus japonicus* UGTs.
**Table S6.** List of *LORE1* insertions in *Lotus japonicus* mutant lines used.
**Table S7.** List of *Glyma.08G348500* mutants used in this study.
**Table S8.** Relative expression levels of *Lotus japonicus* genes orthologous to soybean genes involved in soyasaponin biosynthesis.
**Table S9.** *GmCYP72A61* homologues in *Vigna angularis* (cultivar Longxiaodou No. 4) and *Lotus japonicus* (ecotype MG‐20).
**Table S10.** *Lotus japonicus* genes co‐expressed with *LjUGT73K6* (*Lj3g3v3776600*).
**Table S11.** Locations of *CYP72A61* and *UGT73K5* homologues in the chromosomes of species belonging to Fabaceae and other families.
**Table S12.** PCR primers used in this study.

**Figure S1.** Gene expression profiles of characterised soyasaponin biosynthesis enzyme genes and uncharacterised genes constituting the co‐expression network in soybean.
**Figure S2.** Mutation positions in *UDP‐glycosyltransferase* (*UGT*) genes identified by co‐expression analysis.
**Figure S3.** MS and MS/MS analyses of soyasaponins αg and βa.
**Figure S4.** LC‐PDA‐MS analysis of seed hypocotyl extracts and their hydrolysates from mutant lines deficient in *Glyma.16G033700*, the gene encoding an enzyme candidate that conjugates DDMP to the C‐22 position of the triterpene skeleton.
**Figure S5.** MS and MS/MS analyses of soyasaponins Ac, Ba and Bb.
**Figure S6.** Co‐segregation of mutations in *Glyma.08g348500*, a candidate gene for the UGT that catalyses the conjugation of arabinose to C‐22 position in group A saponins, with the absence of the first sugar moiety at the C‐22 position of group A saponins.
**Figure S7.** LC‐PDA‐MS/MS analysis of compound Y3 purified from EnT‐3537 mutant seeds.
**Figure S8.** Preparation of recombinant soybean UDP‐glycosyltransferases (UGTs).
**Figure S9.** *In vitro* enzyme assay of UGT73BU1 (Glyma.08G348500) using UDP‐glucose, UDP‐galactose or UDP‐glucuronic acid as sugar donors.
**Figure S10.** Phylogenetic tree of soybean *CYPs* involved in soyasaponin biosynthesis and their homologues in *Lotus japonicus*.
**Figure S11.** Expression profiles of *Lotus japonicus* genes involved in soyasaponin biosynthesis.
**Figure S12.** *In vivo* yeast assay to evaluate LjCYP72A61 enzyme activity.
**Figure S13.** Genetic complementation of the *Lj3g3v3776580* mutation by *Lj3g3v3776580* and *GmCYP72A61* in *Lotus japonicus* hairy roots.
**Figure S14.** Genomic organisation of *UGT73K5*, *GmCYP72A61* and their homologues in legume species.
**Figure S15.** LC‐MS/MS analysis of crude extracts from roots and shoots of *Medicago truncatula*, *Lotus japonicus* and soybean.
**Figure S16.** Phylogenetic tree of *CYP72A61* homologues from soybean and *Vigna angularis* (cultivar Longxiaodo No. 4).
**Figure S17.** Subcellular localisation of UGT73K6 and CYP72A61 in *Lotus japonicus* hairy roots.
**Figure S18.** Distribution of saponins and genomic organisation of C‐22 modification enzyme genes in leguminous plants.

## Data Availability

The data that support the findings of this study are found within the article and the associated Supporting Information.
