# Supplementary material for: A modular UDP‐glycosyltransferase and cytochrome P450 system for soyasaponin C‐22 modification
Source: Plant J. 2026 Oct 16;128(1):e71149. doi: 10.1111/tpj.71149 (PMC13673397; doi:10.1111/tpj.71149)
Supplement: Supplementary file 1 [file TPJ-128-0-s001.pdf]

Table S1. List of guide and intermediate genes in co-expression analysis

Category[1]	Z score[2]	Gene ID (Glyma 2.0)	Enzyme activity	Annotation	Gene symbol	Expression (TPM)[3,4]								
						Seed	Root hair	Shoot apical meristem	Root	Root nodule	Stem	Pod	Flower	Leaf
Guide	N/A[5]	*Glyma.07G001300*	2,3-Oxidosqualene cyclase	GmbAS1	*GmbAS1*	19.99	122.04	66.84	88.90	55.21	52.80	24.29	12.66	13.85
Guide	N/A	*Glyma.08G350800*	24-Hydroxylase	GmCYP93E1	*GmCYP93E1*	805.41	386.31	143.51	123.55	88.76	230.69	77.99	19.77	34.82
Guide	N/A	*Glyma.08G238100*	22-Hydroxylase	GmCYP72A61	*GmCYP72A61*	441.57	301.74	155.66	207.82	147.69	131.51	81.45	23.99	23.28
Guide	N/A	*Glyma.15G243300*	21-Hydroxylase	GmCYP72A69	*Sg-5*	33.66	46.57	5.64	70.52	18.48	2.35	1.31	0.60	1.32
Guide	N/A	*Glyma.06G324300*	UDP-glucuronosyl transferase	GmCSyGT1	*GmCSyGT1*	89.66	213.10	68.99	122.59	98.41	129.56	27.69	5.38	7.69
Guide	N/A	*Glyma.11G053400*	UDP-galactosyl transferase	GmUGT73P2	*GmSGT2*	146.78	107.81	44.70	48.80	65.23	46.44	25.75	8.27	20.04
Guide	N/A	*Glyma.01G046300*	UDP-arabinosyl transferase	GmUGT73P10	*Sg-4*	56.36	53.25	0.09	109.31	7.97	0.03	0.09	0.39	0.10
Guide	N/A	*Glyma.08G181000*	UDP-rhamnosyl transferase	GmUGT91H9	*GmGmSGT3*	102.01	154.41	111.38	83.45	123.27	112.08	21.56	10.69	9.86
Guide	N/A	*Glyma.10G104700*	UDP-glucosyl transferase	GmUGT91H4	*Sg-3*	31.99	75.50	37.63	46.34	24.51	26.51	10.11	1.46	3.61
Guide	N/A	*Glyma.07G254600*	UDP-glucosyl transferase	GmUGT73F2	*Sg-1b*	73.67	40.86	13.31	14.41	9.26	11.66	19.85	2.26	2.43
Intermediate	314.8	*Glyma.15G221300*	N/A	UGT	N/A	47.75	18.92	74.09	12.41	27.22	13.21	43.73	17.06	16.39
Intermediate	276.4	*Glyma.16G033700*	N/A	UGT	N/A	26.62	67.90	36.46	28.53	34.30	26.04	17.33	8.70	16.00
Intermediate	270.3	*Glyma.18G208300*	N/A	UGT	N/A	10.33	14.62	27.94	8.60	5.45	11.87	6.38	2.29	2.02

[1]Guide functional gene (guide) or significant intermediate connencting guide genes (Intermidiate) in the module presented by SoyNet (https://www.inetbio.org/soynet/, option III. find functional modules).

[2]Z-score output from SoyNet as a measure of the significance of intermediates in the output subnetwork.

[3]Retrieved from data sets of individual soybean genes in Phytozome v14.

[4]TPM = transcripts per million.

[5]Not applicable.

Table S2. List of *Glyma.16G033700* mutants obtained in this study

Line[1]	Nucleotide substitution[2]	Amino acid change	Saponin
EnT-0051	T > A	V34E	- DDMP[3]
EnT-2016	C > T	T197I	- DDMP
EnT-2211	G > A	G209D	- DDMP
EnT-2354	G > A	A369T	- DDMP
EnT-2358	G > A	G203R	- DDMP
EnT-2865	C > T	P384S	- DDMP
EnT-3528	C > T	P384S	
EnT-3129	A > C	T45P	- DDMP
EnT-3234	C > T	L185F	- DDMP
EnT-3303	G > A	S375N	- DDMP
EnT-0237	C > T	A357V	No change
EnT-1023	C > T	A357V	No change
EnT-4082	C > T	A357V	No change
EnT-0330	C > T	T42I	No change
EnT-0331	C > T	T42I	No change
EnT-0445	A > G	D58G	No change
EnT-0456	G > A	E314K	No change
EnT-0631	C > T	T397M	No change
EnT-0633	C > T	A36V	No change
EnT-3440	C > T	A36V	No change
EnT-0715	G > A	E198K	No change
EnT-0791	G > A	G340D	No change
EnT-0812	G > A	V306M	No change
EnT-0898	G > A	S256N	No change
EnT-1377	G > A	V399M	No change
EnT-1437	G > A	G377E	No change
EnT-1750	G > A	G313R	No change
EnT-2036	C > T	S190F	No change
EnT-2082	A > G	Q39R	No change
EnT-2228	G > A	E410K	No change
EnT-2329	C > A	H400Q	No change
EnT-2462	C > T	P75L	No change
EnT-2592	C > T	S284F	No change
EnT-2596	C > T	A50V	No change
EnT-2709	C > T	A62V	No change
EnT-2764	C > T	R308S	No change
EnT-2804	C > T	S320F	No change
EnT-2827	C > T	P47L	No change
EnT-3050	C > T	S320F	No change
EnT-3101	G > A	A31T	No change
EnT-3118	C > T	S322F	No change
EnT-3182	C > T	S266L	No change
EnT-3285	G > A	A347T	No change
EnT-3485	C > T	Q290M	No change
EnT-3630	C > T	P348S	No change
NEnT-010	C > T	T45I	No change
EnT-0060	C > T	A369V	N/D[4]
EnT-0062	C > T	L261F	N/D
EnT-2006	C > T	A48V	N/D
EnT-2550	G > A	V406M	N/D
EnT-2751	G > A	G80V	N/D

[1]Screened by HRM and validated by Sanger sequencing. Underlined lines were also identified by forward genetic screening (Krishnamurthy et al., 2019).

[2]Change in the coding strand.

[3]Absence of or reduction in seed DDMP saponins.

[4]Not determined.

Table S3. List of *Glyma.15G221300* mutants obtained in this study

Line[1]	Nucleotide substitution[2]	Amino acid change	Saponin
EnT-4023	G > A	A34T	N/D[3]
EnT-4092	C > T	A38V	N/D
EnT-0980	C > T	S59F	N/D
EnT-3713	G > A	G67D	N/D
EnT-3601	G > C	A69P	N/D
EnT-0574	G > A	G143E	N/D
EnT-2696	C > T	S158F	N/D
NEnT-126	C > T	P196L	N/D
EnT-2527	C > T	T206I	N/D
EnT-0481	G > A	G267R	N/D
EnT-1361	C > T	A269V	N/D
EnT-1152	G > A	E272L	N/D
EnT-0689	A > G	E278G	N/D
EnT-1459	G > A	G279R	N/D
EnT-0466	C > T	S286F	N/D
EnT-0103	G > A	S332N	N/D
EnT-1189	A > T	D339V	N/D
EnT-0026	G > A	A361T	N/D
EnT-0238	C > T	P362S	N/D
EnT-1392	C > T	L365F	N/D
EnT-0657	C > T	T377I	N/D
EnT-0757	T > A	W397R	N/D
EnT-0614	C > T	P398S	N/D
EnT-0432	C > T	S478F	N/D
EnT-2931	G > A	frame shift	no change

[1]Screened by HRM and validated by Sanger sequencing.

[2]Change in the coding strand.

[3]Not determined.

Table S4. Result of *Glyma.18G208300* mutant screening by amplicon sequencing

DNA Pool[1]	Nucleotide substitution[2]	Amino acid change	Line	Saponin
Pool35	A > T	K160*	EnT-6141[3]	no change
Pool4	G > A	D357N	N/D[4]	N/D
Pool8	A > T	R486W	N/D	N/D
Pool9	G > T	R162I	N/D	N/D
Pool13	G > A	A434T	N/D	N/D
Pool16	G > A	V247I	N/D	N/D
Pool31	G > A	W370*	N/D	N/D
Pool43	G > A	A268T	N/D	N/D
Pool65	G > A	E2K	N/D	N/D
Pool71	G > A	R159K	N/D	N/D
Pool85	G > A	G78D	N/D	N/D
Pool95	C > T	P45L	N/D	N/D

[1]Mixture of genomic DNAs from 16 mutants (Tsuda et al. 2015).

[2]Change in the coding strand.

[3]Isolated by second screening with HRM, and validation with Sanger sequencing.

[4]Not determined.

Table S5. Amino acid sequence identities among soybean and *Lotus japonicus* UGTs

	GmUGT73K5	Lj3g3v3776600	Lj4g3v2376260	Lj4g3v2376190	Lj4g3v2376170	Lj4g3v2376180	GmUGT73F2	Lj2g3v1903270	GmUGT73P10	GmUGT73P2	GmUGT91H4	GmUGT91H9	Lj4g3v0338060	Lj6g3v2218820
GmUGT73K5	100.0													
**Lj3g3v3776600**	**69.8**	100.0												
Lj4g3v2376260	45.5	48.8	100.0											
Lj4g3v2376190	45.2	47.5	86.2	100.0										
Lj4g3v2376170	45.1	47.9	71.4	73.1	100.0									
Lj4g3v2376180	44.8	48.4	84.2	91.1	82.2	100.0								
GmUGT73F2	44.6	44.5	64.0	63.4	60.9	64.4	100.0							
Lj2g3v1903270	38.0	37.2	38.7	40.7	39.0	40.0	38.5	100.0						
GmUGT73P10	36.8	36.7	37.9	40.7	38.0	39.3	37.0	63.9	100.0					
GmUGT73P2	36.0	36.8	37.4	39.6	37.9	38.4	37.8	78.3	66.5	100.0				
GmUGT91H4	23.9	24.3	24.7	23.2	24.4	24.6	23.0	20.3	21.5	19.8	100.0			
GmUGT91H9	22.7	23.3	24.4	23.4	25.1	24.1	23.0	21.0	21.2	20.0	72.5	100.0		
Lj4g3v0338060	21.6	22.8	24.3	23.1	24.5	24.6	23.1	20.7	20.5	19.7	70.8	79.1	100.0	100.0
Lj6g3v2218820	21.6	22.8	24.3	23.1	24.5	24.6	23.1	20.7	20.5	19.7	70.8	79.1	100.0	

Table S6. List of *LORE1* insertions in *Lotus japonicus* mutant lines used

Line	Plant ID	Chromosome[1]	Position	Orientation[2]	Gene[3]	Insertion type	Annotation
1	30058798	0	76,458,415	F	*Lj0g3v0162169*	Intronic	NA[4]
		3	45,606,985	F	*Lj3g3v3776600*<br>*Lj3g3v3776610*	Exonic<br>Exonic	Glycosyltransferase GT18P15 [*Pueraria montana* var. lobata] gi|319759272|gb|ADV71372.1| Non-coding[5]
		0	54,657,087	F		Intergenic	NA
		2	30,930,984	F	*Lj2g3v2017620*	Exonic	Heavy metal P-type ATPase [*Medicago truncatula*] gi|357518503|ref|XP_003629540.1|
		1	37,045,642	F	*Lj1g3v3315220*	Intronic	NA
		0	91,040,646	F	*Lj0g3v0186689*	Exonic	Non-coding
		0	51,134,703	F	*Lj0g3v0116289*	Exonic	PREDICTED: Glutamic acid-rich protein-like [*Cicer arietinum*] gi|502099577|ref|XP_004491513.1|
		2	7,949,554	F		Intergenic	NA
2	A01254	4	6,305,352	R	*Lj4g3v0450430*<br>*Lj4g3v0450440*	Exonic<br>Exonic	PREDICTED: Receptor-like protein kinase 5-like [*Vitis vinifera*] gi|225470187|ref|XP_002268274.1| PREDICTED: Receptor-like protein kinase 5-like [*Vitis vinifera*] gi|225470187|ref|XP_002268274.1|
		3	45,606,234	R	*Lj3g3v3776600*<br>*Lj3g3v3776610*	Exonic<br>Exonic	Glycosyltransferase GT18P15 [*Pueraria montana* var. lobata] gi|319759272|gb|ADV71372.1| Non-coding
		4	34,836,936	R	*Lj4g3v2603610*	Intronic	NA
		4	6,359,295	F	*Lj4g3v0450560*	Exonic	PREDICTED: Epoxide hydrolase 2-like [*Glycine max*] gi|356548757|ref|XP_003542766.1|
		0	175,277,522	F	*Lj0g3v0334779*	Intronic	NA
		3	45,089,104	R		Intergenic	NA
		5	27,689,412	F	*Lj5g3v1958060*	Intronic	NA
		1	42,944,004	F	*Lj1g3v3729370*	Exonic	PREDICTED: Epoxide hydrolase 2-like [*Glycine max*] gi|356548757|ref|XP_003542766.1|
		0	91,313,014	F	*Lj0g3v0187179*	Exonic	PREDICTED: Serine/threonine protein phosphatase 2A 59 kDa regulatory subunit B' gamma isoform-like [*Glycine max*] gi|356571427|ref|XP_003553878.1|
3	30094065	0	166,349,048	F	*Lj0g3v0318519*	Intronic	NA
		5	30,131,204	F		Intergenic	NA
		0	74,754,135	R	*Lj0g3v0159479*<br>*Lj0g3v0159489*	Exonic<br>Exonic	RNA-directed DNA polymerase [*Arachis hypogaea*] gi|342365825|gb|AEL30359.1| Non-Coding
		0	159,787,730	F	*Lj0g3v0306299*	Exonic	PREDICTED: Puromycin-sensitive aminopeptidase-like [*Cicer arietinum*] gi|502156330|ref|XP_004510420.1|
		3	45,601,698	F	*Lj3g3v3776580*	Exonic	PREDICTED: Secologanin synthase-like isoform 1 [*Glycine max*] gi|356526435|ref|XP_003531823.1|
		4	30,937,644	F	*Lj4g3v2238980*	Exonic	Cytosolic purine 5'-nucleotidase [*Medicago truncatula*] gi|357479189|ref|XP_003609880.1|
4	30054528	0	162,530,889	F	*Lj0g3v0311279*	Intronic	NA
		3	45,600,950	R	*Lj3g3v3776580*	Exonic	PREDICTED: Secologanin synthase-like isoform 1 [*Glycine max*] gi|356526435|ref|XP_003531823.1|
		0	64,620,097	R		Intrergenic	NA
		5	3,559,763	F	*Lj5g3v0380100*	Intronic	NA

[1]Chromosome 0 corresponds to scaffolds not assigned to any pseudomolecule.

[2]Orientation of *LORE 1* insertion relative to nucleotide sequence of pseudomolecule.

[3]Genes of interests were underlined.

[4]Not applicable.

[5]An exon corresponds to predicted non-coding transcript.

Table S7. List of G*lyma.08G348500* mutants used in this study

Line[1]	Nucleotide substitution[2]	Amino acid change	Saponin[3]
EnT-2743	G > A	G209S	- Ab, -Ac, + Y3 and + Y4
EnT-3124	N/F[4]	N/A[5]	- Ab, -Ac, + Y3 and + Y4
EnT-3537	C > T	T380I	- Ab, -Ac, + Y3 and + Y4

[1]Identified by forward genetic screening (Krishnamurthy et al., 2019).

[2]Change in the coding strand.

[3]Absence of saponin Ab and Ac, and accumulation of Y3 and Y4 (Krishnamurthy et al., 2019).

[4]Not found in coding sequence.

[5]Not applicable.

Table S8. Relative expression levels of *Lotus japonicus* genes orthologous to soybean genes involved in soyasaponin biosynthesis

Gene ID	Annotation	Probe(s) mapped to*	WT_control1	WT_Drought1	Ljgln2_2_Control1	Ljgln2_2_Drought1	root_4dpicontrol1B	root_4dpimycorrhized1D	root_28dpicontrol1A	root_28dpimycorrhized1C	WT_root_tip_3w_uninocul_1	WT_root_3w_uninocul_1	WT_root_3w_5mM_nitrate_1	WT_root_6w_5mM_nitrate_1	WT_shoot_3w_5mM_nitrate_1	WT_shoot_3w_uninocul_1	WT_shoot_3w_inocul3_1	WT_leaf_6w_5mM_nitrate_1	WT_stem_6w_5mM_nitrate_1	WT_flower_13w_5mM_nitrate_1	har1_root_3w_uninocul_2	har1_root_3w_inocul3_2	har1_shoot_3w_uninocul_1	har1_shoot_3w_inocul3_1	WT_root_3w_nodC_inocul1_1	WT_root_3w_inocul1_1	WT_root_3w_inocul3_1	WT_nodule_3w_inocul14_1	WT_nodule_3w_inocul21_1	WT_root_nodule_3w_inocul7_1	WT_root_nodule_3w_inocul21_1
Lj3g3v3776600	LjUGT73K6	chr3.TM0797.5.2_at	0.06	0.07	0.11	0.06	0.26	0.24	0.36	0.24	0.42	0.58	0.66	0.20	0.13	0.14	0.20	0.09	0.11	0.11	0.41	0.37	0.07	0.18	0.52	0.31	0.81	0.10	0.11	0.21	0.51
Lj3g3v3776580	LjCYP72A61	chr3.TM0797.5_at<br>chr3.TM0797.5.1_at	0.16	0.18	0.22	0.13	0.46	0.57	0.78	0.64	0.50	0.81	0.73	0.45	0.26	0.30	0.41	0.20	0.29	0.34	0.88	0.98	0.33	0.37	0.68	0.50	0.78	0.20	0.25	0.47	0.65
Lj1g3v3555800	LjCYP93E1	Ljwgs_008809.1_at	0.08	0.10	0.14	0.05	0.36	0.42	0.62	0.45	0.57	0.81	0.71	0.43	0.20	0.27	0.31	0.12	0.16	0.16	0.77	0.72	0.27	0.31	0.76	0.50	0.87	0.16	0.21	0.43	0.65
Lj3g3v2027430	LjOSC1 (bAS)	chr3.CM0292.16_s_at<br>Ljwgs_141303.1_s_at<br>chr3.CM0292.35_s_at	0.17	0.16	0.31	0.12	0.03	0.02	0.01	0.00	0.33	0.48	0.47	0.15	0.40	0.40	0.58	0.26	0.29	0.10	0.17	0.23	0.04	0.66	0.52	0.29	0.73	0.09	0.10	0.20	0.58

Gene ID	WT_rootSZ_3w_uninocul_1	WT_rootSZ_3w_Nod_inocul1_1	WT_rootSZ_3w_inocul1_1	nfr5_rootSZ_3w_uninocul_1	nfr5_rootSZ_3w_inocul1_1	nfr1_rootSZ_3w_uninocul_1	nfr1_rootSZ_3w_inocul1_1	nup133_rootSZ_3w_uninocul_1	nup133_rootSZ_3w_inocul1_1	cyclops_root_3w_uninocul	cyclops_root_nodule_3w_inocul21	nin_rootSZ_3w_uninocul_1	nin_rootSZ_3w_inocul1_1	sen1_root_3w_uninocul_1	sen1_nodule_3w_inocul21_1	sst1_root_3w_uninocul_1	sst1_nodule_3w_inocul21_1	cyclops_root_3w_inocul	Shoot_0mM_sodiumChloride_1	Shoot_25mM_sodiumChloride_Initial_1	Shoot_50mM_sodiumChloride_Initial_1	Shoot_75mM_sodiumChloride_Initial_1	Shoot_50mM_sodiumChloride_Gradual_1	Shoot_100mM_sodiumChloride_Gradual_1	Shoot_150mM_sodiumChloride_Gradual_1
Lj3g3v3776600	0.34	0.26	0.34	0.44	0.37	0.55	0.26	0.34	0.29	0.24	0.61	0.56	0.44	0.49	0.27	0.51	0.18	0.44	0.06	0.09	0.11	0.06	0.06	0.09	0.10
Lj3g3v3776580	0.57	0.46	0.47	0.61	0.58	0.55	0.48	0.54	0.48	1.00	0.80	0.72	0.60	0.78	0.52	0.76	0.44	0.72	0.17	0.22	0.25	0.20	0.20	0.20	0.24
Lj1g3v3555800	0.63	0.51	0.50	0.74	0.70	0.62	0.49	0.58	0.51	0.65	0.80	0.73	0.59	0.73	0.43	0.76	0.36	0.66	0.08	0.12	0.13	0.09	0.09	0.11	0.12
Lj3g3v2027430	0.31	0.26	0.35	0.61	0.59	0.31	0.23	0.37	0.32	0.11	0.59	0.53	0.67	0.49	0.19	0.44	0.14	0.58	0.12	0.14	0.23	0.14	0.13	0.15	0.19

Gene ID	Lburttii_Ctrol_A	Lburttii_Salt_A	Lcorniculatus_Ctrol_A	Lcorniculatus_Salt_A	Lfilicaulis_Ctrol_A	Lfilicaulis_Salt_A	Lglaber_Ctrol_A	Lglaber_Salt_A	Ljaponicus_Gifu_Ctrol_A	Ljaponicus_Gifu_Salt_A	Ljaponicus_MG20_Ctrol_A	Ljaponicus_MG20_Salt_A	Luliginosus_Ctrol_A	Luliginosus_Salt_A	Fl_1	Pod20_1	Seed10d_1	Seed12d_1	Seed14d_1	Seed16d_1	Seed20d_1	Leaf_1	Pt_1	Stem_1	Root_1	Root0h_1	Nod21_1
Lj3g3v3776600	0.24	0.18	0.43	0.29	0.09	0.08	0.28	0.31	0.04	0.04	0.06	0.05	0.06	0.08	0.17	0.03	0.04	0.03	0.00	0.00	0.01	0.12	0.12	0.14	0.53	1.00	0.23
Lj3g3v3776580	0.14	0.12	0.29	0.24	0.11	0.10	0.23	0.26	0.13	0.10	0.23	0.21	0.16	0.18	0.41	0.17	0.10	0.06	0.00	0.01	0.12	0.16	0.29	0.29	0.44	0.64	0.42
Lj1g3v3555800	0.07	0.04	0.27	0.22	0.11	0.10	0.26	0.28	0.07	0.05	0.08	0.06	0.09	0.10	0.13	0.12	0.03	0.02	0.00	0.01	0.08	0.05	0.13	0.16	0.66	1.00	0.30
Lj3g3v2027430	0.15	0.16	0.43	0.47	0.06	0.05	0.55	0.71	0.16	0.17	0.23	0.25	0.06	0.07	0.18	0.07	0.03	0.03	0.00	0.01	0.01	0.24	0.35	0.46	0.43	1.00	0.15

*ID of Affymetrix *Lotus* GeneChip probes to which the listed genes mapped with the best scores by reciprocal blast search.

**Values normalised across condition.

Table S9. *GmCYP72A61* homologues in *Vigna anguralis* (cultivar Longxiaodou No.4) and *Lotus japonicus* (ecotype MG-20)

ID	Species	Amino acid sequence indentity (%)[1]	E value[2]	Chromosome	Gene start	Gene end	Strand
XP_017440758	*V. anguralis*	91.3	0	9	8,751,092	8,753,999	-
XP_017425717	*V. anguralis*	58.2	0	11	24,385,897	24,394,415	+
XP_017424978	*V. anguralis*	57.4	0	11	24,340,256	24,344,276	+
XP_017425757	*V. anguralis*	57.2	0	11	24,397,099	24,400,127	-
XP_017424979	*V. anguralis*	57.0	0	11	24,353,035	24,356,494	+
XP_017416965	*V. anguralis*	56.7	0	2	48,454,369	48,457,357	-
XP_017425716	*V. anguralis*	56.6	0	11	24,385,897	24,394,415	+
XP_017425534	*V. anguralis*	55.3	0	11	24,411,723	24,415,443	+
XP_017431403	*V. anguralis*	50.5	0	5	29,767,377	29,770,172	+
XP_017432596	*V. anguralis*	50.4	0	5	5,738,682	5,741,889	-
XP_017431308	*V. anguralis*	49.6	0	5	29,592,848	29,595,649	+
XP_017433623	*V. anguralis*	48.5	0	5	5,769,922	5,778,250	-
LjCYP72A61	*L. japonicus*	86.1	0	3	45,599,818	45,601,938	-

[1]Identity with GmCYP72A61.

[2]E value of BLASTP search with GmCYP72A61 as a query.

Table S10. ***Lotus japonicus*** **genes co-expressed with** ***LjUGT73K6*** **(*Lj3g3v3776600*)**[1]

Gene ID	Annotation	R^2
Lj3g3v2027430	LjOSC1 (bAS)	0.961
Lj3g3v1981230	LjCSyGT	0.939
Lj2g3v1903270	PREDICTED: anthocyanin 3'-O-beta-glucosyltransferase-like [Glycine max] gi|356541764|ref|XP_003539343.1|	0.919
Lj1g3v3555800	LjCYP93E1	0.916
Lj0g3v0024229	PREDICTED: probable rhamnose biosynthetic enzyme 1-like [Glycine max] gi|356525600|ref|XP_003531412.1|	0.909
Lj3g3v3776580	LjCYP72A61	0.907
Lj6g3v2218850	soyasaponin III rhamnosyltransferase [Glycine max] gi|359807261|ref|NP_001240857.1|	0.906
Lj6g3v2218860	PREDICTED: soyasaponin III rhamnosyltransferase-like [Cicer arietinum] gi|502077094|ref|XP_004485550.1|	0.906
Lj1g3v0116670	PREDICTED: histone deacetylase HDT3-like [Fragaria vesca subsp]. vesca] gi|470134384|ref|XP_004303033.1|	0.903
Lj0g3v0284109	PREDICTED: probable C-terminal domain small phosphatase-like isoform X4 [Cicer arietinum] gi|502170470|ref|XP_004514839.1|	0.903
Lj2g3v1091850	malonyl-CoA:isoflavone 7-O-glucoside-6''-O-malonyltransferase [Glycine max] gi|351722557|ref|NP_001237760.1|	0.899
Lj1g3v0984180	PREDICTED: probable pectinesterase/pectinesterase inhibitor 34-like [Cicer arietinum] gi|502136316|ref|XP_004502642.1|	0.891
Lj1g3v4515510	PREDICTED: serine/threonine-protein kinase CBK1-like [Glycine max] gi|356503620|ref|XP_003520605.1|	0.889
Lj2g3v1090830	PREDICTED: phenolic glucoside malonyltransferase 1-like [Cicer arietinum] gi|502091178|ref|XP_004489467.1|	0.888
Lj1g3v1409270	PREDICTED: random slug protein 5-like [Cicer arietinum] gi|502133430|ref|XP_004501761.1|	0.886
Lj6g3v0440060	PREDICTED: phenolic glucoside malonyltransferase 1-like [Cicer arietinum] gi|502091174|ref|XP_004489466.1|	0.879
Lj5g3v2240960	PREDICTED: diphosphomevalonate decarboxylase-like [Cicer arietinum] gi|502121015|ref|XP_004497159.1|	0.875
Lj2g3v1550280	PREDICTED: rop guanine nucleotide exchange factor 1-like [Glycine max] gi|356496475|ref|XP_003517093.1|	0.873
Lj0g3v0331219	PREDICTED: spindle and kinetochore-associated protein 3 [Callithrix jacchus] gi|390463958|ref|XP_003733140.1|	0.870
Lj4g3v0620420	PREDICTED: UDP-glucose flavonoid 3-O-glucosyltransferase 7-like [Cicer arietinum] gi|502143184|ref|XP_004505250.1|	0.867

[1]Co-expressed genes were retrieved by Correlated Genes Identifier for *L. japonicus* reference genome v3 (https://lotus.au.dk/tools/corgi) using *Lj3g3v3776600* (*LjUGT73K6*) as a query. Data set used was *L. japonicus* early root response to inoculation with microbes along the symbiont-pathogen spectrum-MG20 v3.0 genome.

Table S11. Locations of *CYP72A61* and *UGT73K5* homologues in the chromosomes of species belonging to Fabaceae and other families

Species	Family	*CYP72A61*					*UGT73K5*					Distance (kbp)[4]	Source database
		ID	e value[1]	% Identity[2]	Chromosome	gene_coordinate[3]	ID	e value	% identity	Chromosome	gene_coordinate		
Lotus japonicus	*Fabaceae*	*Lj3g0022341*	0	88	3	151,164..153,480	*Lj3g0015818*	0	71	3	145,493..147,260	8	https://phytozome-next.jgi.doe.gov/
Trifolium alexandrinum	*Fabaceae*	*TalF1.0_TalF1.0ch4_000477.1*	0	82	4	5,931,219..5,935,066	*TalF1.0_TalF1.0ch4_000478.1*	0	63	4	5,937,384..5,939,022	8	https://genome.kazusa.or.jp/home/
Medicago truncatula	*Fabaceae*	*Medtr4g031820*	0	85	4	11,016,191..11,019,940	*Medtr4g031800*	0	65	4	11,022,930..11,024,756	9	https://phytozome-next.jgi.doe.gov/
Cicer arietinum	*Fabaceae*	*Ca_19813*	0	84	6	36,764,656..36,770,391	*Ca_19814*	0	64	6	36,741,805..36,743,263	29	https://phytozome-next.jgi.doe.gov/
Glycyrrhiza uralensis	*Fabaceae*	*Glur_chr3.g060870*	0	85	3	32,668,119..32,672,930	*Glur_chr3.g060750*	0	70	3	32,564,572..32,566,014	108	https://genome.kazusa.or.jp/
Lens culinaris	*Fabaceae*	*Lcu.2RBY.4g014940*	0	84	4	79,950,493..79,953,730	*Lcu.2RBY.4g014970*	0	64	4	80,058,102..80,059,870	109	https://phytozome-next.jgi.doe.gov/
Vicia faba	*Fabaceae*	*Vfaba.Hedin2.R1.2g229720*	0	84	2	1,453,451,181..1,453,454,011	*Vfaba.Hedin2.R1.2g229680*	0	63	2	1,453,109,572..1,453,111,030	344	https://phytozome-next.jgi.doe.gov/
Vigna anguralis	*Fabaceae*	*XP_017440758*	0	91	9	8,751,092..8,753,999	*XP_017408866*	0	76	9	7,815,254..7,816,923	939	https://www.ncbi.nlm.nih.gov/datasets/genome/GCF_016808095.1/
Pisum sativum	*Fabaceae*	*PSA1.0ch7VII.g522871*	0	83	7	467,971,452..467,974,568	*PSA1.0ch7VII.g523125*	0	64	7	469,547,486..469,548,892	1,577	https://genome.kazusa.or.jp/
Glycine max (queries)	*Fabaceae*	*Glyma.08G238100*	N/A[5]	N/A	8	20,521,261..20,523,593	*Glyma.16G033700*	N/A	N/A	16	3,117,746-3,120,047	N/A	https://phytozome-next.jgi.doe.gov/
Phaseolus vulgaris	*Fabaceae*	*Phvul.011G000200*	0	93	11	16,878..19,828	*Phvul.010G101800*	0	76	10	37,085,643..37,087,392	N/A	https://phytozome-next.jgi.doe.gov/
Arachis hypogaea	*Fabaceae*	*AhLine8.07G088000*	0	85	7	14,490,934..14,494,894	*AhLine8.03G281700*	0	67	3	125,914,584..125,916,604	N/A	https://phytozome-next.jgi.doe.gov/
Lupinus albus	*Fabaceae*	*Lalb_Chr01g0016181*	0	58	1	16,517,998..16,521,022	*Lalb_Chr08g0238021*	0	68	8	11,381,982..11,383,893	N/A	https://phytozome-next.jgi.doe.gov/
Cajanus cajan	*Fabaceae*	*C.cajan_20884*	0	92	4	210,947..213,740	*C.cajan_20762*	1E-126	74	1	16,715,893..1,671,733	N/A	https://plants.ensembl.org/
Chamaecrista fasciculata	*Fabaceae*	*ChfasH1.2G118200.1.p*	0	58	2	45,660,566..45,665,946	*ChfasH1.4G165200.1.p*	1.67E-120	41	4	55,189,631..55,191,873	N/A	https://phytozome-next.jgi.doe.gov/
Panax ginseng	*Araliaceae*	*Pgi_18G016310.1*	0	56	18	114,442,959..114,446,566	*Pgi_15G018240.1*	3.92E-153	47	15	111,248,500..111,250,307	N/A	https://biobigdata.nju.edu.cn/plant2t/home
Prunus persica	*Rosaceae*	*Prupe.4G153000.1.p*	0	59	4	8,774,715..8,778,208	*Prupe.8G130800.1.p*	5.93E-127	44	8	15,353,205..15,354,845	N/A	https://phytozome-next.jgi.doe.gov/
Solanum tuberosum	*Solanaceae*	*Soltu.DM.07G021190.1*	0	56	7	51,488,312..51,491,344	*Soltu.DM.12G005820.1*	1.97E-131	42	12	4,764,753..4,766,211	N/A	https://phytozome-next.jgi.doe.gov/
Saponaria officinalis	*Caryophyllaceae*	*Sapof.12G220600.1.p*	0	53	12	134,006,743..134,012,133	*Sapof.08G118300.1.p*	5.25E-136	43	8	116,117,858..116,119,958	N/A	https://phytozome-next.jgi.doe.gov/
Cucumis melo	*Cucurbitaceae*	*Cme_821_H2_07G003520.1*	0	56	7	3,113,920..3,117,112	*Cme_821_H2_11G002730.1*	2.34E-115	39	11	2,241,673..2,244,204	N/A	https://biobigdata.nju.edu.cn/plant2t/home

[1]e value by BLASTP search using the predicted amino acid sequences of soybean *CYP72A61* or *UGT73K5* as queries.

[2]Amino acid identity with the predicted amino acid sequences of soybean *CYP72A61* or *UGT73K5*.

[3]Position of both ends of each gene in chromosome (distance from the end of the chromosome, base pairs).

[4]Distance between two genes when located on the same chromosome (distance between the most distant nucleotide, base pairs).

[5]Not applicable.

Table S12. PCR primers used in this study

ID	Primer name	Sequence (5´ to 3´)	Target	Usage
1	Glyma.16G033700-F1	TTCCCCATGCTGTTACGTATA	*Glyma.16G033700*	HRM[1] and genotyping of soybean mutants by sequencing
2	Glyma.16G033700-R1	TGTCGATGTTCTGGTCGAAG	*Glyma.16G033700*	HRM and genotyping of soybean mutants by sequencing
3	Glyma.16G033700-F2	TCTTCTCACCGGGGCACCTAATCCC	*Glyma.16G033700*	HRM and genotyping of soybean mutants by sequencing
4	Glyma.16G033700-R2	GCATGATGAGATGCGCCGCCATGT	*Glyma.16G033700*	HRM and genotyping of soybean mutants by sequencing
5	Glyma.16G033700-F4	ATCCACGCCATTAAAACCCA	*Glyma.16G033700*	HRM and genotyping of soybean mutants by sequencing
6	Glyma.16G033700-R4	GGTTTCCAGTTTGGAATAAGA	*Glyma.16G033700*	HRM and genotyping of soybean mutants by sequencing
7	Glyma.16G033700-F5	TGTTACAGGGCGCAAGGTGT	*Glyma.16G033700*	HRM and genotyping of soybean mutants by sequencing
8	Glyma.16G033700-R5	CTTCGAGTCCGGTAGCAATCTGG	*Glyma.16G033700*	HRM and genotyping of soybean mutants by sequencing
9	Glyma.16G033700-F6	ATTGTCTCACGTGGCTTGACTC	*Glyma.16G033700*	HRM and genotyping of soybean mutants by sequencing
10	Glyma.16G033700-R6	AGTGCGTTAAAAATCCTCCC	*Glyma.16G033700*	HRM and genotyping of soybean mutants by sequencing
11	Glyma.16G033700-F7	ACAGAGGCATGCTGATTAAGGG	*Glyma.16G033700*	HRM and genotyping of soybean mutants by sequencing
12	Glyma.16G033700-R7	CCTCTTCACGGCACTCTCTATCC	*Glyma.16G033700*	HRM and genotyping of soybean mutants by sequencing
13	Glyma.16G033700-F10	GAGTAAGAGAAGAGATGGA	*Glyma.16G033700*	genotyping of soybean mutants by sequencing
14	Glyma.16G033700-R10	GGAACTTGATGATGTGGAC	*Glyma.16G033700*	genotyping of soybean mutants by sequencing
15	Glyma.16G033700-F11	GTCTCACGTGGCTTGACTCA	*Glyma.16G033700*	genotyping of soybean mutants by sequencing
16	Glyma.16G033700-R11	AGAGCCGTGAGACTGTCGTA	*Glyma.16G033700*	genotyping of soybean mutants by sequencing
17	Glyma.15G221300_ex1_1F	CAGTGTGCAAGCATTCCAAT	*Glyma.15G221300*	HRM and genotyping of soybean mutants by sequencing
18	Glyma.15G221300_ex1_1R	TGGTGACATCAACACCATGA	*Glyma.15G221300*	HRM and genotyping of soybean mutants by sequencing
19	Glyma.15G221300_ex1_2F	TCATGGTGTTGATGTCACCATA	*Glyma.15G221300*	HRM and genotyping of soybean mutants by sequencing
20	Glyma.15G221300_ex1_2R	GAACATGTCGGTGAACAAGAAA	*Glyma.15G221300*	HRM and genotyping of soybean mutants by sequencing
21	Glyma.15G221300_ex1_3F	TGCAGCCTGATTTCTTGTTC	*Glyma.15G221300*	HRM and genotyping of soybean mutants by sequencing
22	Glyma.15G221300_ex1_3R	TGCTTGTCATGTAATCCAGCA	*Glyma.15G221300*	HRM and genotyping of soybean mutants by sequencing
23	Glyma.15G221300_ex2_1F	GGCTAAAAGTTGGGACTTATTCT	*Glyma.15G221300*	HRM and genotyping of soybean mutants by sequencing
24	Glyma.15G221300_ex2_1R	ATTCAACAGCGATCCATAGC	*Glyma.15G221300*	HRM and genotyping of soybean mutants by sequencing
25	Glyma.15G221300_ex2_2F	ACAAGAAGGCCATGGGAACT	*Glyma.15G221300*	HRM and genotyping of soybean mutants by sequencing
26	Glyma.15G221300_ex2_2R	CATGGTCTGAATCCTCAAG	*Glyma.15G221300*	HRM and genotyping of soybean mutants by sequencing
27	Glyma.15G221300_ex2_3F	TGCCCTTGAGGATTCAGACC	*Glyma.15G221300*	HRM and genotyping of soybean mutants by sequencing
28	Glyma.15G221300_ex2_3R	TCCAATGGCATGATGCTCTA	*Glyma.15G221300*	HRM and genotyping of soybean mutants by sequencing
29	Glyma.15G221300_ex2_4F	AGAGCATCATGCCATTGGA	*Glyma.15G221300*	HRM and genotyping of soybean mutants by sequencing
30	Glyma.15G221300_ex2_4R	GACTCTTCACCACCCATCAAA	*Glyma.15G221300*	HRM and genotyping of soybean mutants by sequencing
31	Glyma.15G221300_ex2_5Fkai	TGGGTGGTGAAGAGTCTATAGAAA	*Glyma.15G221300*	HRM and genotyping of soybean mutants by sequencing
32	Glyma.15G221300_ex2_5Rkai	TGTCCTGAATTGATTGCAATTATC	*Glyma.15G221300*	HRM and genotyping of soybean mutants by sequencing
33	I08-18G208300-5k-0907_F	AATAGTTGCATGTGAGGACATTTCCCT	*Glyma.18G208300*	amplicon sequencing
34	I08-18G208300-5k-0907_R	CGATAATCCAGTTTGTGCACGTTGTCAG	*Glyma.18G208300*	amplicon sequencing
36	Glyma.18G208300_F1	CAAGACCTTCAACCAGATT	*Glyma.18G208300*	genotyping of soybean mutants by sequencing
37	Glyma.18G208300_R1	ACTATTATACAGTGCCCCA	*Glyma.18G208300*	genotyping of soybean mutants by sequencing
38	LORE_1_P2	CCATGGCGGTTCCGTGAATCTTAGG	*LORE 1*	genotyping of *L. japonicus* mutants
39	30058798_F	TCAACACATCGGGAGGGCTCTGCT	*Lj3g3v3776600*	genotyping of *L. japonicus* mutants
40	30058798_R	GTCGGGATCCGTCACTGGGTTGAA	*Lj3g3v3776600*	genotyping of *L. japonicus* mutants
41	A01254_F	GGCAGCCACGGCGTTATTGTGAAC	*Lj3g3v3776600*	genotyping of *L. japonicus* mutants
42	A01254_R	CCACCCCAAAACCATGCACCTCTG	*Lj3g3v3776600*	genotyping of *L. japonicus* mutants
43	attB1_16g033700Fw	GGGGACAAGTTTGTACAAAAAAGCAGGCTATGGAAAGAGTAGCATCAGTATCTCGGC	*Glyma.16G033700*	cloning of *Glyma.16G033700* CDS
44	16g033700cDNA_attB2_Rv	GGGGACCACTTTGTACAAGAAAGCTGGGTTCAGTGATCGATATTCCCATTCAC	*Glyma.16G033700*	cloning of *Glyma.16G033700* CDS
45	attB1_3776600cDNA_Fw	GGGGACAAGTTTGTACAAAAAAGCAGGCTATGGAGACGTTGAAAGTATACTTCGTTCC	*Lj3g3v3776600*	cloning of *Lj3g3v3776600* CDS
46	3776600cDNA_attB2_Rv	GGGGACCACTTTGTACAAGAAAGCTGGGTTTAGTTTGCAGGTTTGGTGACAAGGGTTC	*Lj3g3v3776600*	cloning of *Lj3g3v3776600* CDS
47	08g348500_HRM_F2	AATCATAACCCTCCTCACGC	*Glyma.08G348500*	sequencing of *Glyma.08G348500* CDS in mutants
48	08g348500HRM_R1	GGGATGCTATTGTTAAGTGTGG	*Glyma.08G348500*	sequencing of *Glyma.08G348500* CDS in mutants
49	08g348500E1Rv	ACCTGATCCTCTTGTGGCTTG	*Glyma.08G348500*	sequencing of *Glyma.08G348500* CDS in mutants
50	08g348500E2Fw	GTCGACGACGAGTGCCTC	*Glyma.08G348500*	sequencing of Glyma.08G348500 CDS in mutants
51	08g348500_HRM_F4	CCATTGGAGGGTTCTTAACGC	*Glyma.08G348500*	sequencing of Glyma.08G348500 CDS in mutants
52	08g348500_HRM_R3	GTCACCAAACCTAGGCATGG	*Glyma.08G348500*	sequencing of Glyma.08G348500 CDS in mutants
53	attL1_08g348500E1Fw	*acaaaaaagcaggct* ATGGAAGACTTAGAAGCAAAG	*Glyma.08G348500*	cloning of *Glyma.08G348500* CDS
54	08g348500E2_AttL2Rv	acaagaaagctgggtTCAACCATGAACATTCATCAT	*Glyma.08G348500*	cloning of *Glyma.08G348500* CDS
55	30054528_F	ATTCGCACACAGCTCTTGGGACCG	*Lj3g3v3776580*	genotyping of *L. japonicus* mutants
56	30054528_R	CTCGCCTTTGCTTGCCAATCAGGA	*Lj3g3v3776580*	genotyping of *L. japonicus* mutants
57	30094065_F	CGATGGTGTGGACAACGAAAGGCA□	*Lj3g3v3776580*	genotyping of *L. japonicus* mutants
58	30094065_R	TCGTGGGAAGCTCTCGTGGTGGTT	*Lj3g3v3776580*	genotyping of *L. japonicus* mutants
59	attB1_3776580cDNA_Fw	GGGGACAAGTTTGTACAAAAAAGCAGGCTATGGGAGAAATCATGGGAATATCATC	*Lj3g3v3776580*	cloning of *Lj3g3v3776580* CDS
60	3776580cDNA_attB2_Rv	GGGGACCACTTTGTACAAGAAAGCTGGGTCTAGAGTTTATGTAAAATGAGATGAG	*Lj3g3v3776580*	cloning of *Lj3g3v3776580* CDS
61	attB1_08g238100Fw2	AAAAAGCAGGCTATGGGAGGATTAGGACTCAATTTG	*Glyma.08g238100*	cloning of *Glyma.08g238100* CDS
62	08g238100_attB2_Rv	AGAAAGCTGGGTCTAGAGTTTGCGTAAAATGAGATG	*Glyma.08g238100*	cloning of *Glyma.08g238100* CDS
63	attB1_LjDDMP_cds_Fw	AAAAAGCAGGCTTTATGGAGACGTTGAAAGTATACTTCG	*Lj3g3v3776600*	cloning of *Lj3g3v3776600 stop-less* CDS
64	LjDDMP_stopless_attB2_Rv	AGAAAGCTGGGTCGTTTGCAGGTTTGGTGACAAG	*Lj3g3v3776600*	cloning of *Lj3g3v3776600 stop-less* CDS
65	attB1_Lj72A61_cds_Fw	AAAAAGCAGGCTTTATGGGAGAAATCATGGGAATATC	*Lj3g3v3776580*	cloning of Lj3g3v3776580 *stop-less* CDS
68	Lj72A61_stopless_attB2_Rv	AGAAAGCTGGGTCGAGTTTATGTAAAATGAGATGAGC	*Lj3g3v3776580*	cloning of *Lj3g3v3776580 stop-less* CDS

[1]High-resolution melt analysis for reverese genetic secreening of mutants.
