# Supplementary material for: A modular UDP‐glycosyltransferase and cytochrome P450 system for soyasaponin C‐22 modification
Source: Plant J. 2026 Oct 16;128(1):e71149. doi: 10.1111/tpj.71149 (PMC13673397; doi:10.1111/tpj.71149)
Supplement: Supplementary file 2 [file TPJ-128-0-s002.pdf]

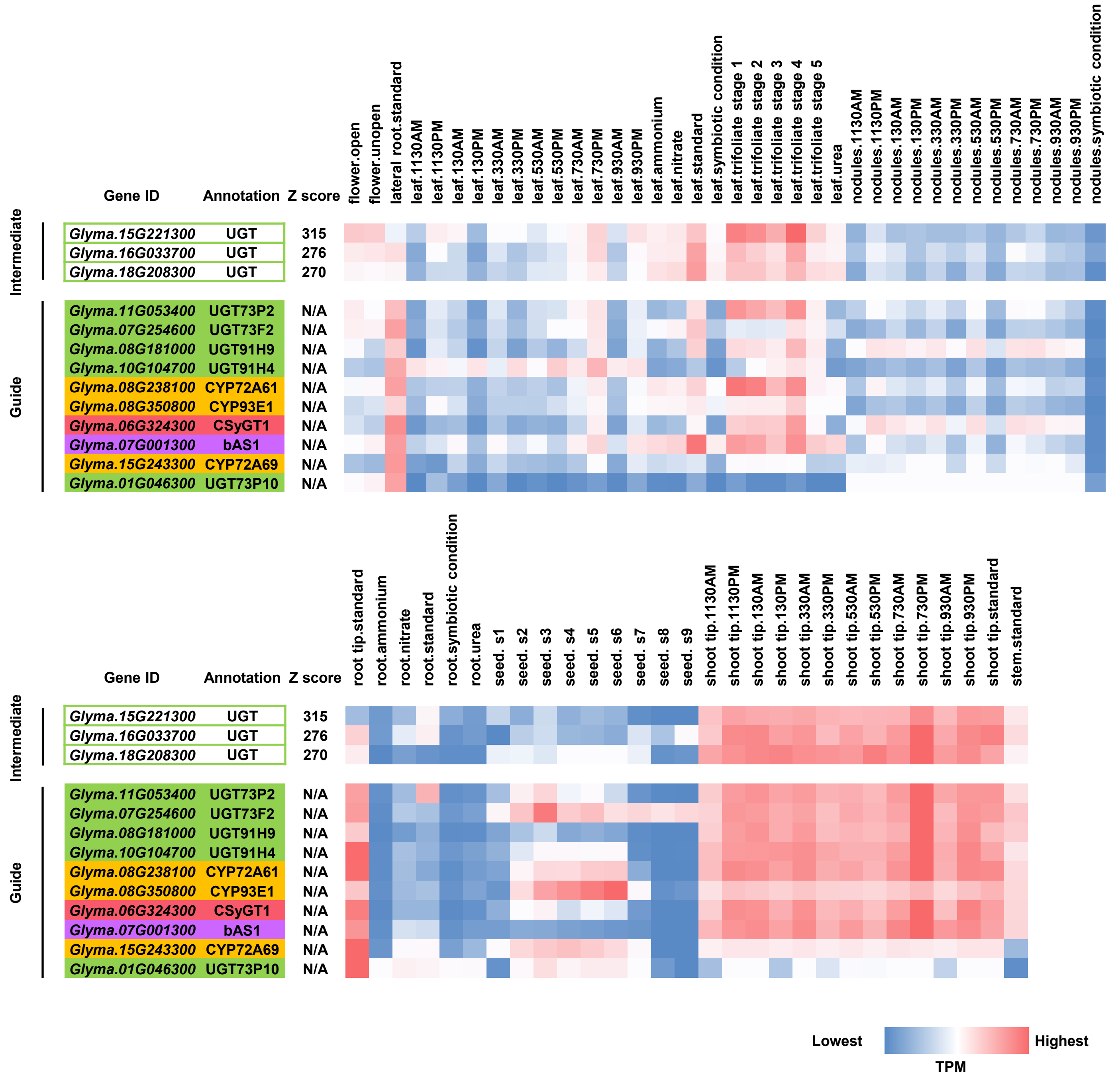

Figure S1. Gene expression profiles of characterised soyasaponin biosynthesis enzyme genes and uncharacterised genes constituting the co-expression network in soybean.

The expression profiles of the characterised soyasaponin biosynthesis enzyme (guide) and uncharacterised (intermediate) genes (see Figure 1B) are shown. The heatmap illustrates the relative abundance of transcripts across various tissues, calculated from the RNA-seq data obtained as transcripts per million (TPM) from the Phytozome database (v14). Phytozome gene IDs, functional annotations, and Z-scores in the co-expression analysis are provided alongside the heatmap. The colour scale represents the expression range of each gene across different organs, from the lowest to the highest expression levels.

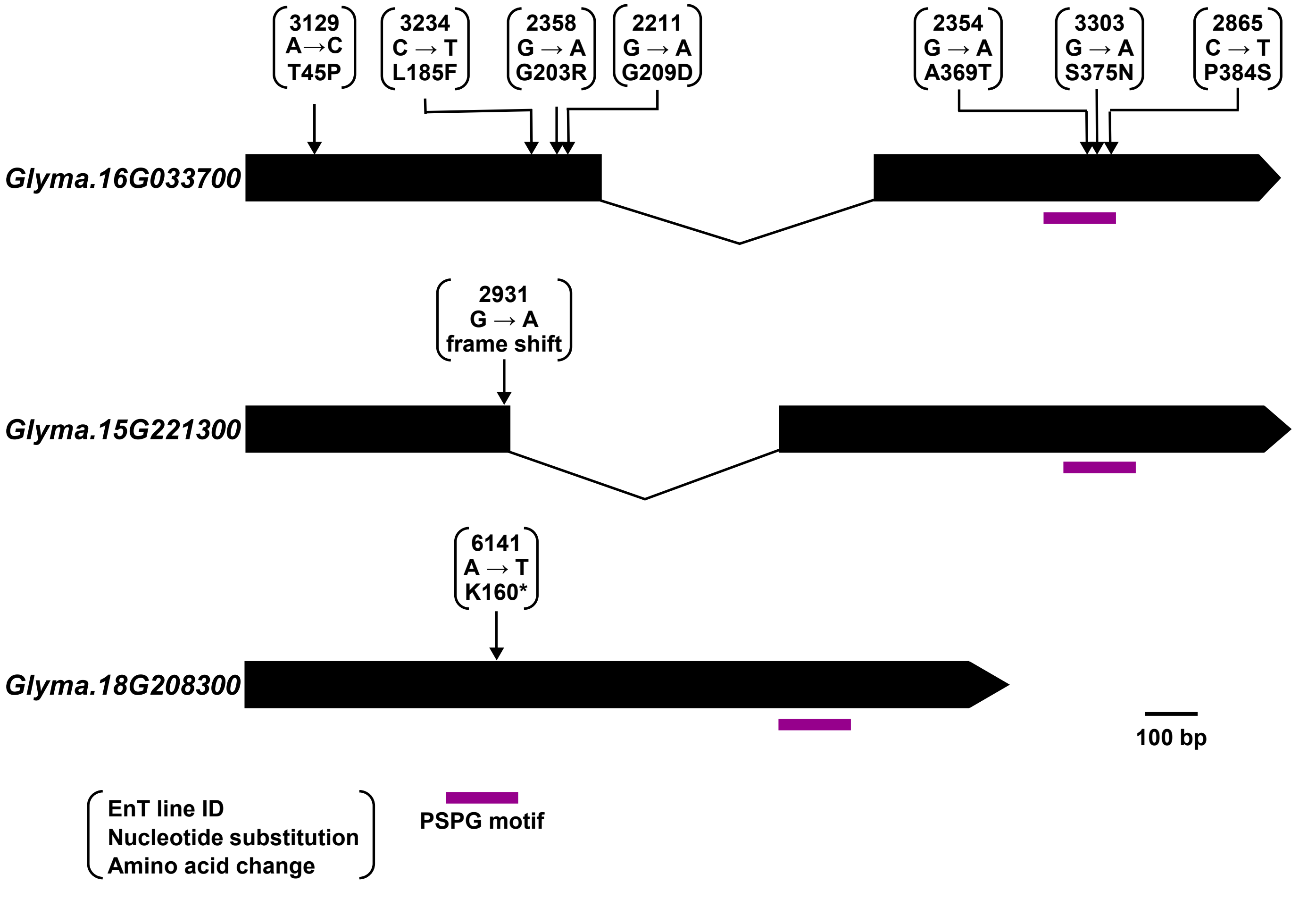

Figure S2. Mutation positions in *UDP-glycosyltransferase* genes identified by co-expression analysis.

The schematics of gene structures indicate the positions of the identified mutations. The mutant line IDs, nucleotide substitutions, and amino acid changes (using a single-letter code, where an asterisk denotes a stop codon) are shown in brackets above each mutation site. Of the 51 *Glyma.16G033700* mutants identified, seven, that were recovered through both forward and reverse genetic screening, are shown. Solid boxes represent exons and angled lines represent introns. The purple line indicates the region encoding the PSPG motif.

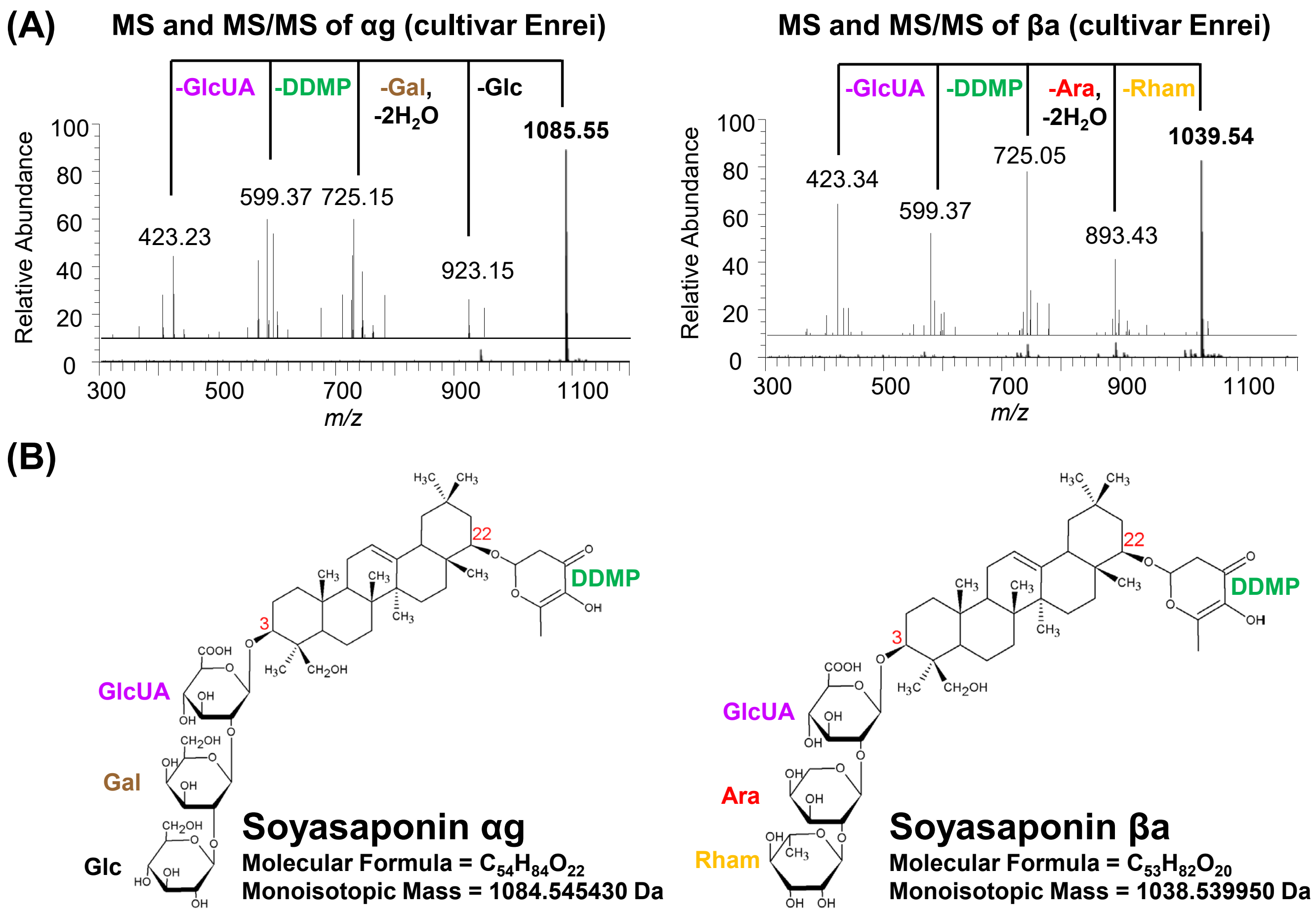

Figure S3. MS and MS/MS analyses of soyasaponins αg and βa.

(A) MS and MS/MS spectra of soyasaponin αg (eluted at 36.3 min) in the hypocotyl extracts and βa (38.6 min) in cotyledon extracts from Enrei seeds (Figure 2A).
(B) Chemical structures of αg and βa. The sugar moieties and DDMP group are colour-coded to correspond to the respective MS/MS fragment patterns.

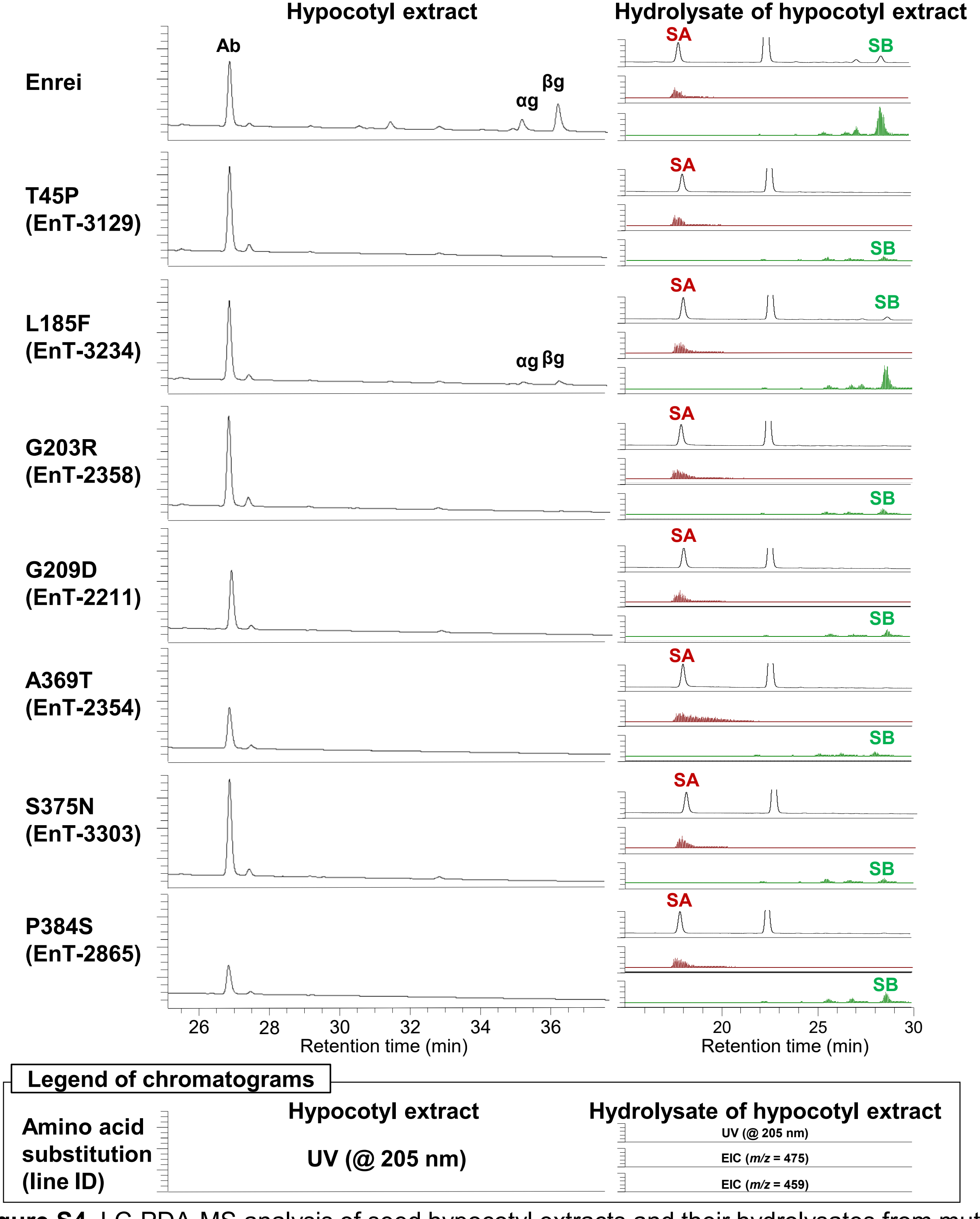

Figure S4. LC-PDA-MS analysis of seed hypocotyl extracts and their hydrolysates from mutant lines deficient in *Glyma.16G033700*, a candidate gene encoding the enzyme responsible for DDMP conjugation at the C-22 position of the triterpene skeleton.

The left panel shows the UV chromatograms (205 nm) of seed hypocotyl extracts from *Glyma.16G033700* mutant lines obtained through forward and reverse genetic screening (see Figure S2 and Table S2). The peaks corresponding to the major soyasaponins, Ab (eluted at 26.8 min), αg (eluted at 35.1 min), and βg (eluted at 36.2 min), are indicated on the chromatograms of the wild-type (WT) and EnT-3234. The right-hand panel shows UV chromatograms (205 nm; upper trace) and extracted ion chromatograms (EICs) for *m/z* = 475 (middle trace, corresponding to soyasapogenol A) and 459 (lower trace, corresponding to soyasapogenol B) obtained from the acid hydrolysate of the respective extracts. The y-axis represents the relative absorbance (UV chromatogram) or ion abundance (EIC). The legends for the chromatograms are presented at the bottom of the figure.

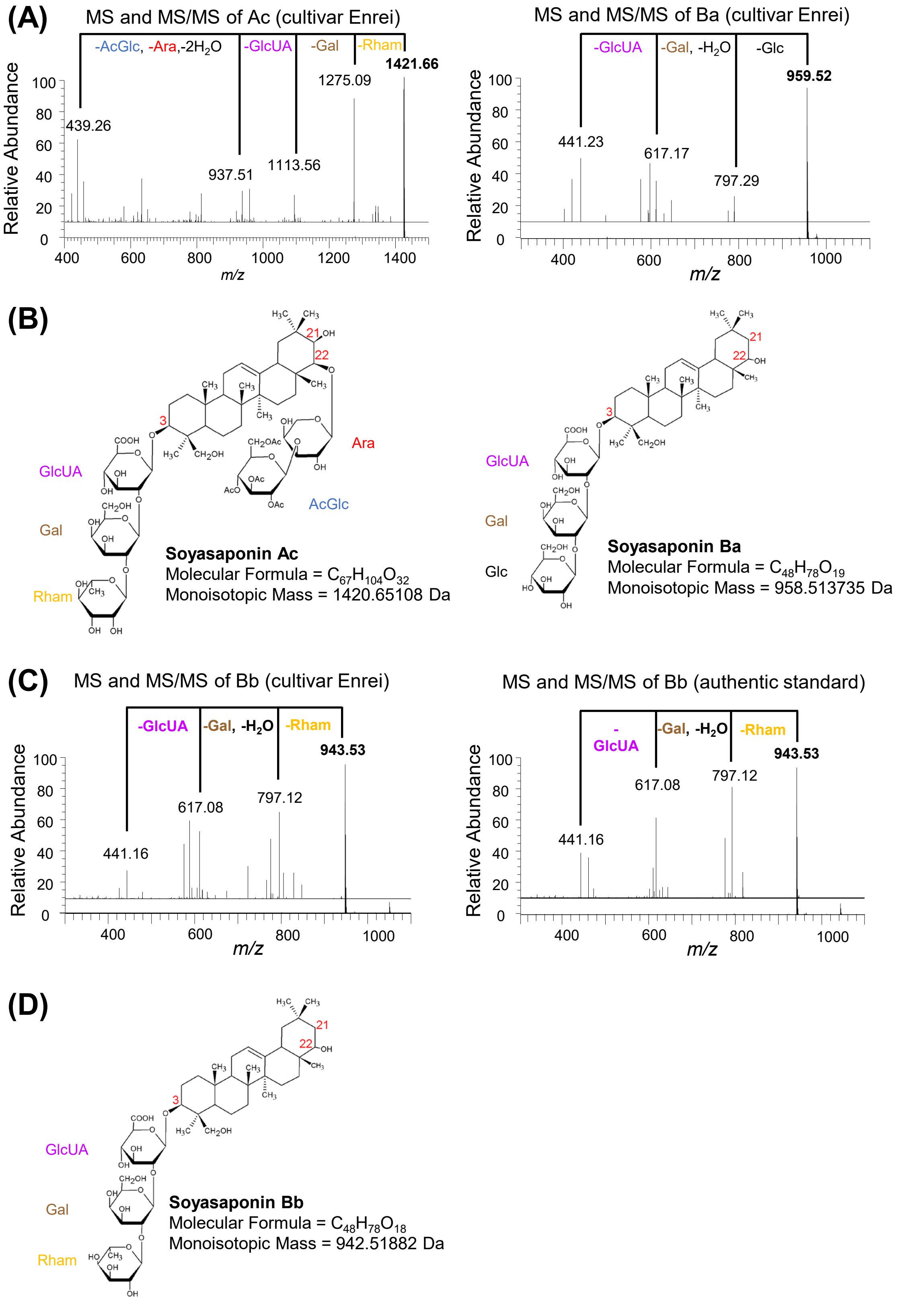

Figure S5. MS and MS/MS analyses of soyasaponins Ac, Ba, and Bb.

(A) MS and MS/MS spectra of Ac (eluted at 28.3 min) and Ba (eluted at 31.0 min) from hypocotyl extracts of Enrei seeds (see Figure 5B).
(B) Chemical structures of Ac and Ba. The sugar and DDMP moieties are coloured to correspond to the MS/MS fragment patterns.
(C) MS and MS/MS spectra of Bb (eluted at 32.0 min) from hypocotyl extracts of Enrei seeds (Figure 5B), alongside authentic standards.
(D) The chemical structure of soyasaponin Bb. The sugar moieties are coloured to correspond to the MS/MS fragment patterns.

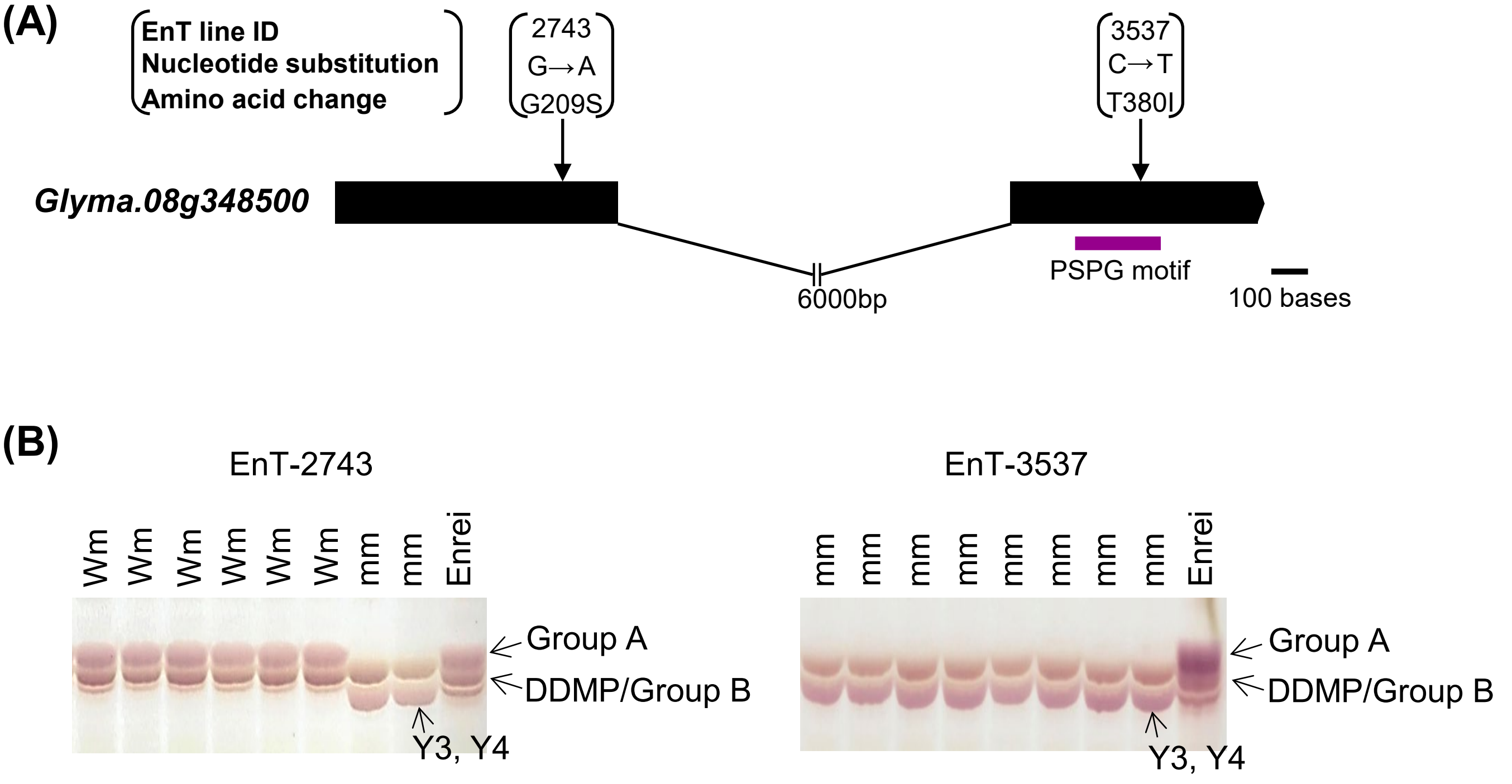

Figure S6. Co-segregation of mutations in *Glyma.08g348500*, a candidate gene for the UDP-glycosyltransferase that catalyses the conjugation of arabinose to C-22 position in group A saponins, with the absence of the first sugar moiety at the C-22 position of group A saponins.

(A) Nucleotide substitutions are indicated above the *Glyma.08G348500* gene schematic alongside the corresponding mutant line IDs, nucleotide substitutions and amino acid changes. The solid box and angled line represent exons and introns, respectively, and the purple line denotes the region encoding the PSPG motif.

(B) TLC of seed hypocotyl extracts from the EnT-2743 and EnT-3537 lines. Seeds harvested from a EnT-2743 heterozygote and a EnT-3537 homozygote were genotyped and subjected to TLC analysis. The positions of group A saponins, DDMP/group B saponins, and compounds Y3 and Y4 are indicated alongside the TLC image. Wm: heterozygote; mm: homozygous mutant.

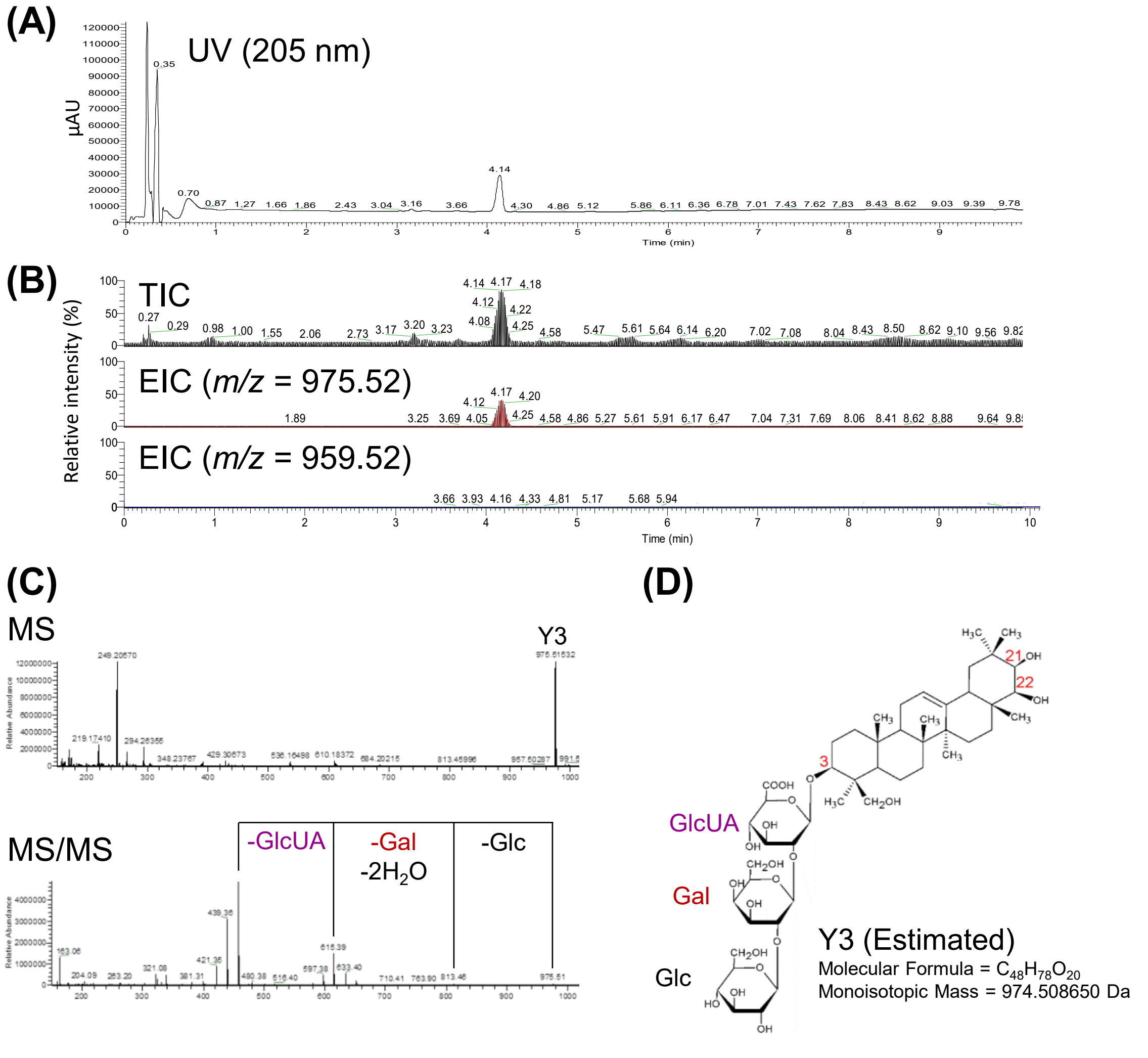

Figure S7. LC-PDA-MS/MS analysis of the compound Y3 that was purified from the EnT-3537 mutant seeds.

Purified compound Y3 was dissolved in methanol and analysed using LC/PDA/MS/MS with a Vanquish Flex Binary System (Thermo Fisher Scientific), which was equipped with a Vanquish Diode Array Detector FG (Thermo Fisher Scientific) and coupled to a hybrid quadrupole-Orbitrap mass spectrometer (Q Exactive Focus, Thermo Fisher Scientific). LC separation was performed on a reverse-phase Develosil FlexFire C30 column (2.0 mm inner diameter x 35 mm, Nomura Chemical) at 40ºC with a flow rate of 0.4 ml/min. The solvent gradient consisted of 20–65%(v.v) acetonitrile (0–12 min), 100% acetonitrile (12–12.5 min) and 10% (v.v) acetonitrile (12.5–14 min), all of which contained 0.1% (v.v) formic acid in water. The Q Exactive Focus MS was operated in positive electrospray ionisation mode with the following parameters: capillary temperature, 320ºC; sheath gas flow rate, 7.0 arbitrary units; aux gas flow rate, 12.5 arbitrary units; spare gas flow rate, 0 arbitrary units; probe heater temperature, 0ºC; collision-energy-injection voltage, 3.76 kV; de-injection voltage, 40 V; and lens 6 voltage, 630 V. MS full scan and subsequent data-dependent MS/MS scans were acquired at a resolution of 70,000 over an *m/z* range of 150–2,000. Parallel reaction monitoring (PRM) was then performed at a resolution of 35,000. Dynamic exclusion was applied in automatic mode during MS/MS and PRM acquisitions. The lock masses used for calibration were acetonitrile/formic acid (*m/z* = 88.03931), methanol/water (*m/z* = 101.08084); and water/formic acid (*m/z* = 65.02332). An inclusion list of 204 compounds, comprising isoflavonoids and saponins, was applied. Polysiloxane (*m/z* = 445.12002) was excluded from the analysis.
(A) UV chromatogram (205 nm).
(B) Total ion chromatogram (TIC) and extracted ion chromatograms (EICs) for m/z = 975.52 (corresponding to compound Y3) and *m/z* = 959.52 (corresponding to compound Y4).
(C) MS and MSMS spectra of Y3.
(D) The estimated structure of Y3.

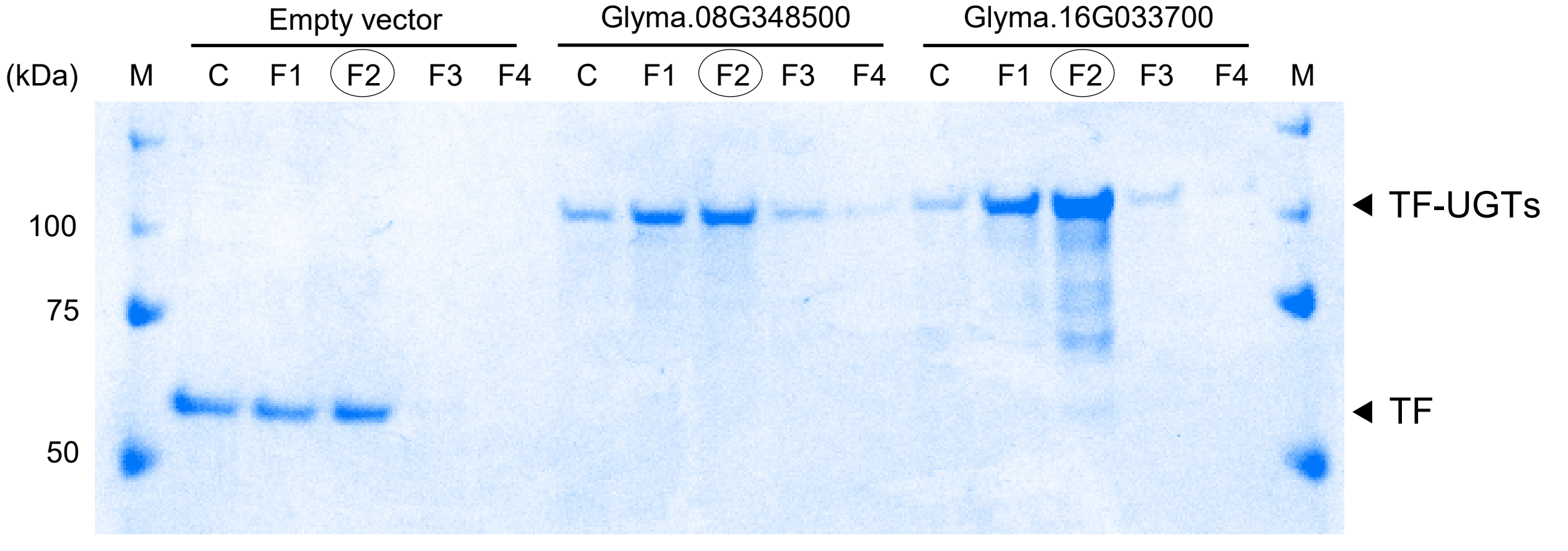

Figure S8. Preparation of recombinant soybean UDP-glycosyltransferases (UGTs).

The cDNAs of *Glyma.08G348500* and *Glyma.16G033700* were amplified by PCR, verified by sequencing, and cloned into a GATEWAY-adapted version of the pCold TF vector (Takara Bio Inc.) using either the pDONR221 or the pENTR/D-TOPO vactors and the Gateway LR Clonase II Enzyme Mix (Thermo Fisher Scientific). The recombinant proteins, which were expressed in *Escherichia coli* as fusion proteins with a trigger factor (TF), were then purified *via* affinity chromatography. Crude extracts (C) and eluted fractions (F1-F4) were analysed using sodium dodecyl sulphate-polyacrylamide gel electrophoresis. 'M' indicates the molecular weight marker. As a negative control, pColdTM TF (empty vector) was expressed in *E. coli*. Fraction F2 (circled) from each *E. coli* line was used for subsequent enzyme assays.

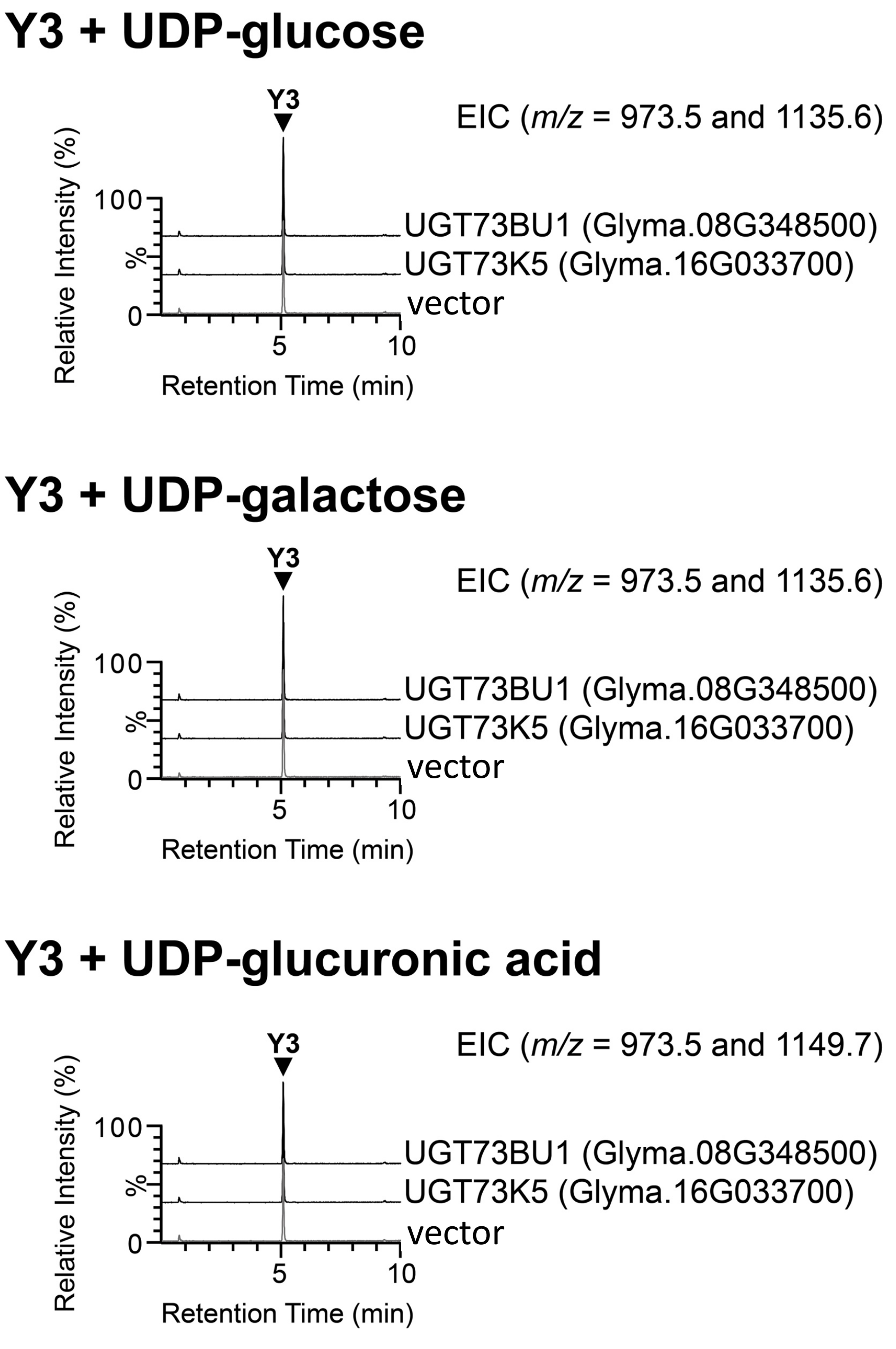

Figure S9. *In vitro* enzyme assay of UGT73BU1 (Glyma.08G348500), using UDP-glucose, UDP-galactose or, UDP-glucuronic acid as sugar donors.

The extracted ion chromatograms (EICs) shown correspond to the following *m/z* values: 973.5 (compound Y3); 1135.6 (Y3-glucoside or Y3-galactoside); and 1149.7 (Y3-glucuronide). These values correspond to the enzyme assay substrate (compound Y3) and the products generated when compound Y3 as the sugar acceptor with different UDP-sugars acting as the donors. Reactions were carried out using purified UGT73BU1 or UGT73K5 enzymes or relevant fractions from an *Escherichia coli* strain containing an empty vector (vector control). Peaks corresponding to Y3 (triangle, *m/z* = 973.5) are indicated in the chromatograms.

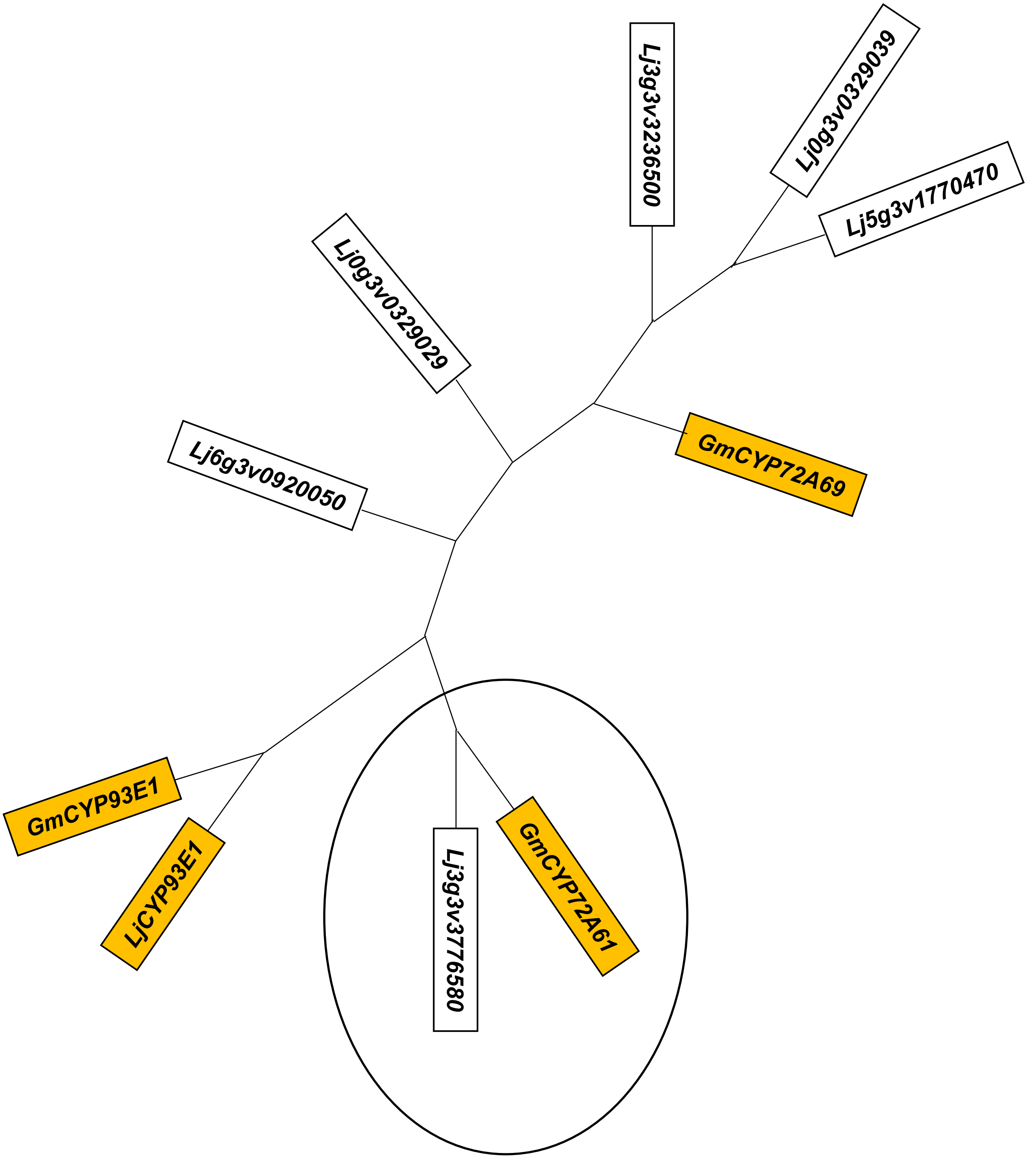

Figure S10. Phylogenetic tree of soybean *CYPs* involved in soyasaponin biosynthesis and their homologues in *Lotus japonicus*.

The amino acid sequences of *GmCYP72A61*, *GmCYP72A69* and *GmCYP93E1*, which are involved in the biosynthesis of soyasaponins (see Figure 1A), were used as queries in BLASTP searches of the Lotus Base (*L. japonicus* MG20 v3.0) protein database. Of the 451 non-redundant sequences showing significant similarity, seven were selected for phylogenetic analysis based on alignments at an e-value of 0. *CYPs* previously reported to be involved in saponin biosynthesis are highlighted in colour.

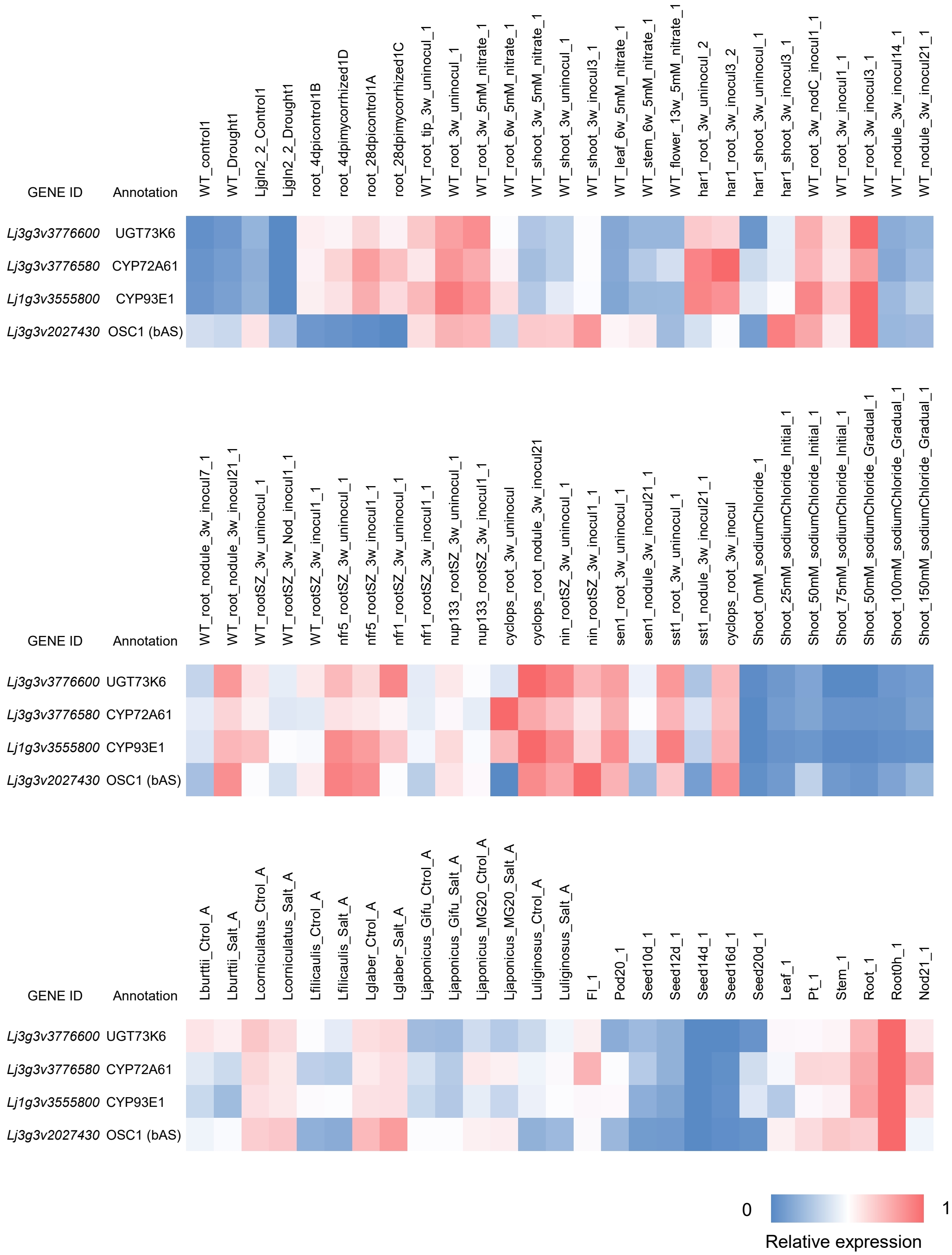

Figure S11. Expression profiles of *Lotus japonicus* genes involved in soyasaponin biosynthesis.

Heatmap showing the relative transcript abundance of soyasaponin biosynthetic genes across different tissues and conditions, based on Affymetrix GeneChip data from Lotus Base (*L. japonicus* Expression Atlas, LjEGA dataset). Gene IDs and annotations from Lotus Base are shown alongside the heatmap. The colour scale represents the expression range for each gene, from the lowest to the highest levels.

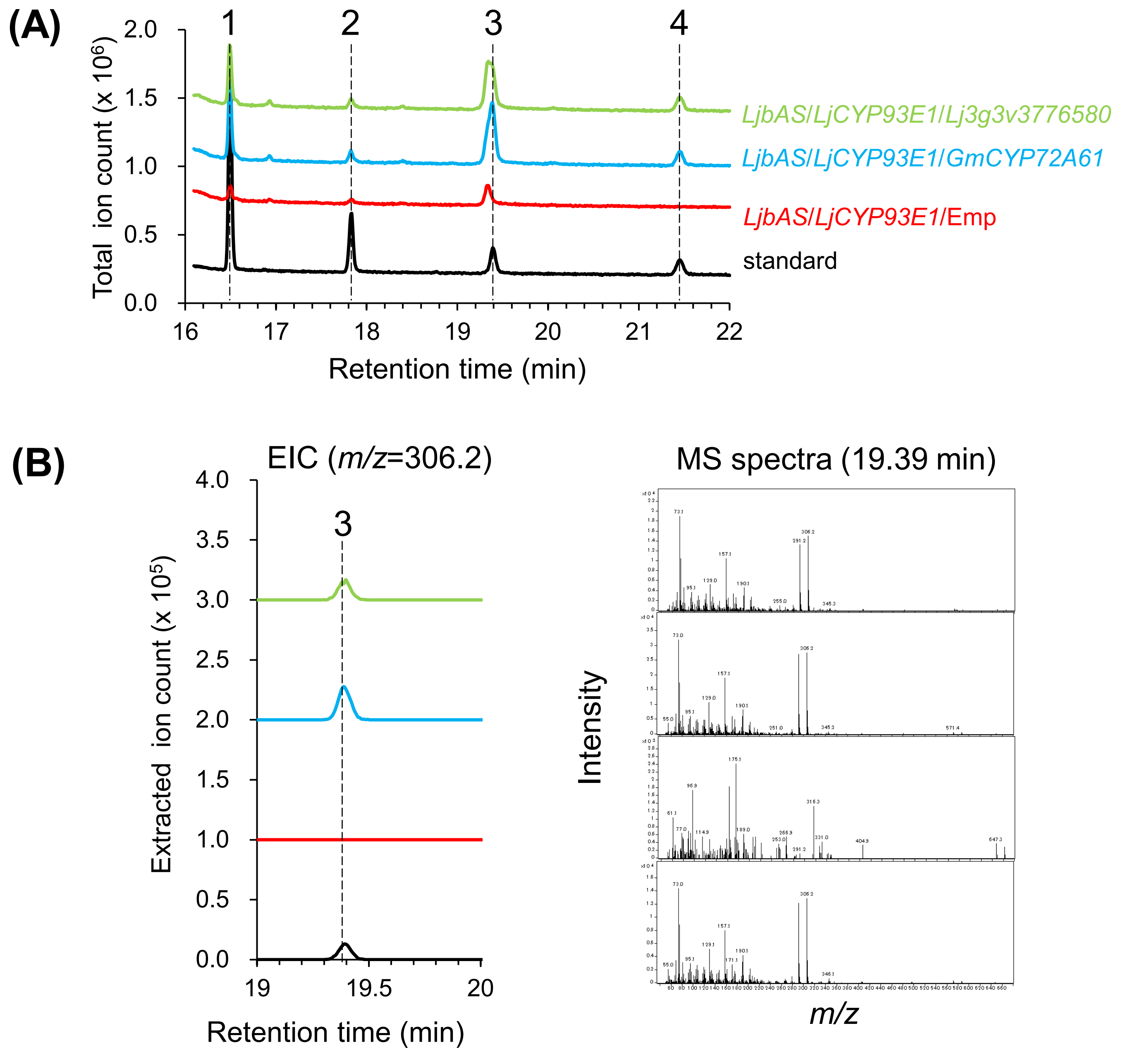

(C)

Peak #	1	2	3	4
Retention time (min)	16.50	17.84	19.39	21.45
Compound	β-amyrin	24-OH-β-amyrin	sophoradiol	soysapogenol B
LjbAS/LjCYP93E1/Lj3g3v3776580	+	+	+	+
LjbAS/LjCYP93E1/GmCYP72A61	+	+	+	+
LjbAS/LjCYP93E1/Emp	+	+	-	-

Figure S12. *In vivo* yeast assay to evaluate the activity of the LjCYP72A61 enzyme.

The *Saccharomyces cerevisiae* strain that has been pre-transformed with *LjbAS*, *LjCYP93E1* and *LjCPR1* was then transformed with one of the followings: *Lj3g3v3776580* (light green), *GmCYP72A61* (light blue), or an empty vector (red). Extracts from the yeast strains were analysed by GC-MS, as described in the Materials and Methods (A). An overlapping peak was observed in the extracts of yeast carrying the empty vector (eluted at 19.33 minutes) and the sophoradiol peak (peak 3) in the extracts of yeast expressing *Lj3g3v3776580* and *GmCYP72A61*. The absence of sophoradiol in the empty vector control sample was confirmed by the extracted ion chromatogram (EIC) at m/z = 306.2, as well as by the mass spectrum acquired at 19.39 minutes (B and C).

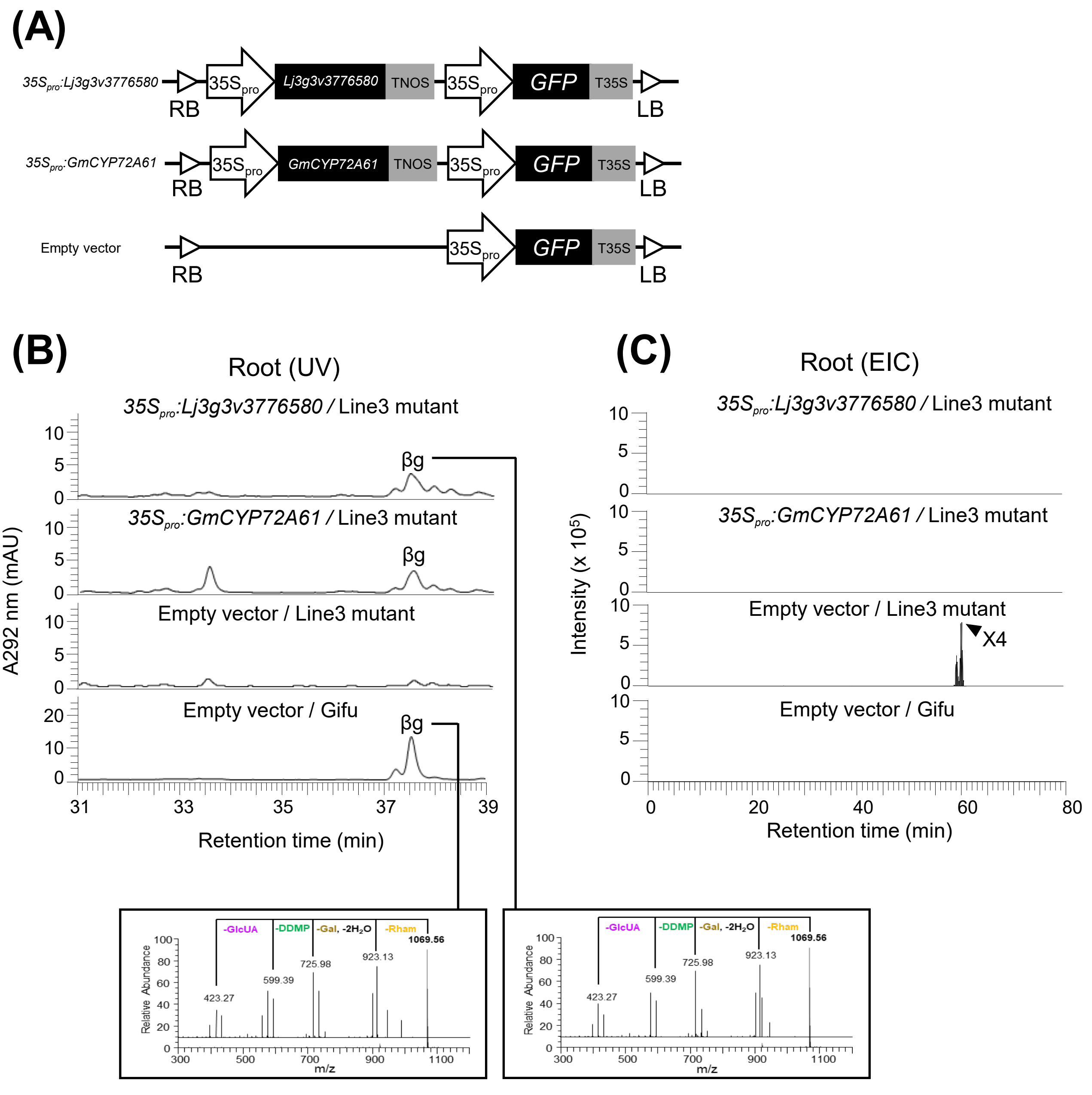

Figure S13. Genetic complementation of the *Lj3g3v3776580* mutation by *Lj3g3v3776580* and *GmCYP72A61* in *Lotus japonicus* hairy roots.

(A) Schematic of the T-DNA constructs introduced into hairy roots. The expression cassettes for *Lj3g3v3776580* and *GmCYP72A61* genes were placed in tandem with a *GFP* expression cassette. An empty vector containing only *GFP* was used as a negative control. RB: right border; 35Spro: CaMV35S promoter; Tnos: *nopaline synthase* terminator; T35S: CaMV 35S terminator; LB: left border.
(B) UV chromatograms (292 nm) of extracts from hairy roots. Extracts were prepared from hairy roots that were developed on a homozygous *Lj3g3v3775800* mutant (line 3, see Figure 7) that was transformed with either *35Spro:GmCYP72A61*, *35Spro:Lj3g3v3775800* or an empty vector. Hairy roots derived from wild-type (ecotype Gifu B-129) plants transformed with the empty vector were used as a control. MS and MS/MS spectra of soyasaponin βg in *35Spro:Lj3g3v3775800* and the control (empty vector/Gifu) are shown below the chromatograms.
(C) Extracted ion chromatograms (EIC) at the theoretical *m/z* value of 927.53, corresponding to compound X4, in the same samples as in panel (B).

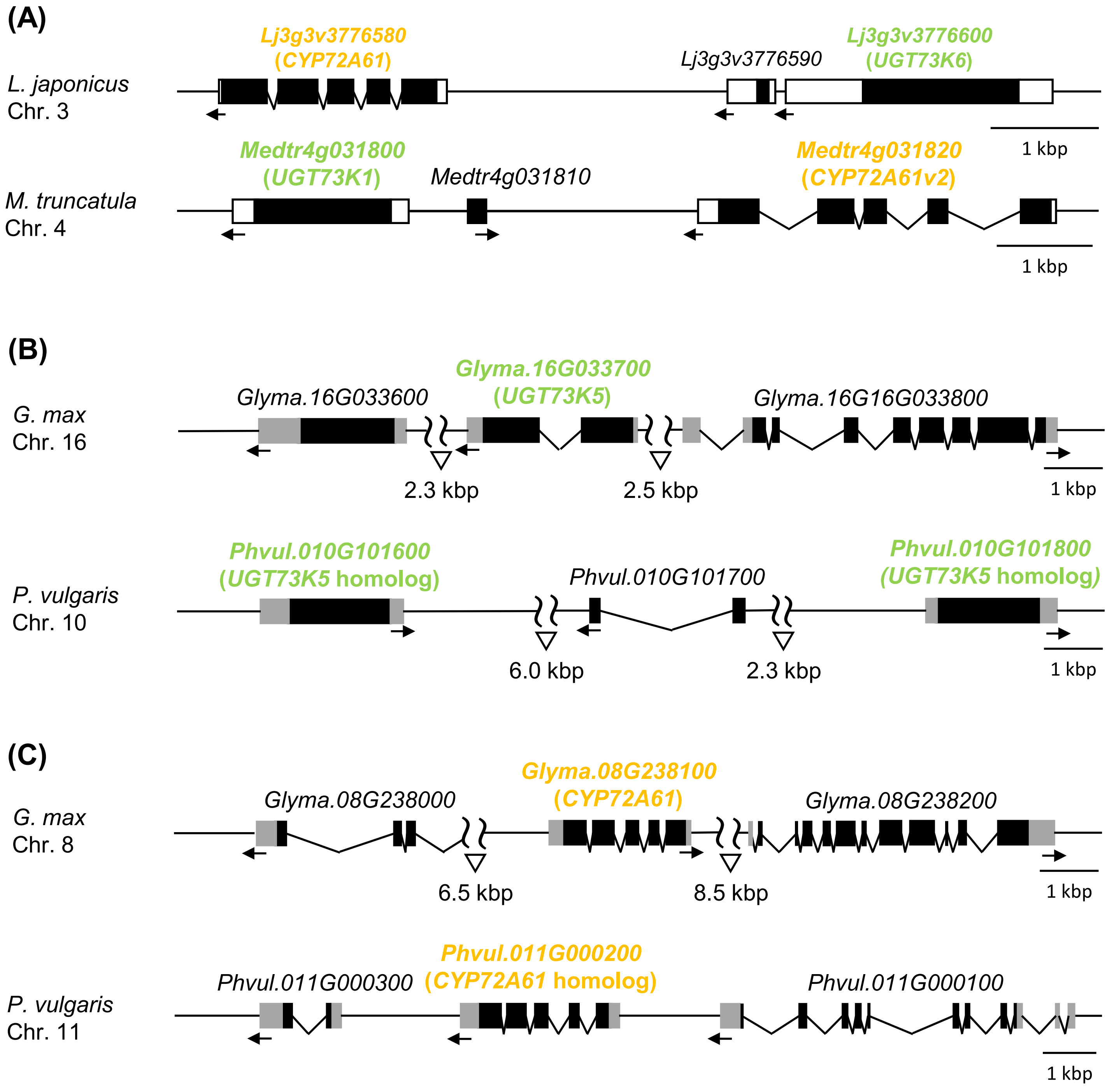

Figure S14. Genomic organisation of *UGT73K5*, *GmCYP72A61* and their homologues in legume species.

Gene positions and structures were visualised using genomic sequences and annotations retrieved from Lotus Base (*Lotus japonicus*) and Phytozome v12.1 (other species). *UGT73K1* and *MtCYP72A61v2* in *Medicago truncatula*, along with homologues in *Phaseolus vulgaris*, were identified as the top hits in a TBLASTN search using the predicted amino acid sequences of *UGT73K5* and *GmCYP72A61* as queries. Filled boxes, angled lines, and grey boxes represent exons, introns, and untranslated regions in the gene models, respectively. Arrows indicate the direction of transcription. Large intergenic regions are depicted as gaps with white triangles representing their sequence lengths. Gene IDs and annotations from the respective databases are shown in each schematic diagram.
(A) Genomic regions of *L. japonicus* and *M. truncatula* that display the tandem arrangement of *UGT73K5* and *GmCYP72A61* homologues.
(B) Genomic regions surrounding *UGT73K5* and its *P. vulgaris* homologues.
(C) Genomic regions surrounding *GmCYP72A61* and its *P. vulgaris* homologues.

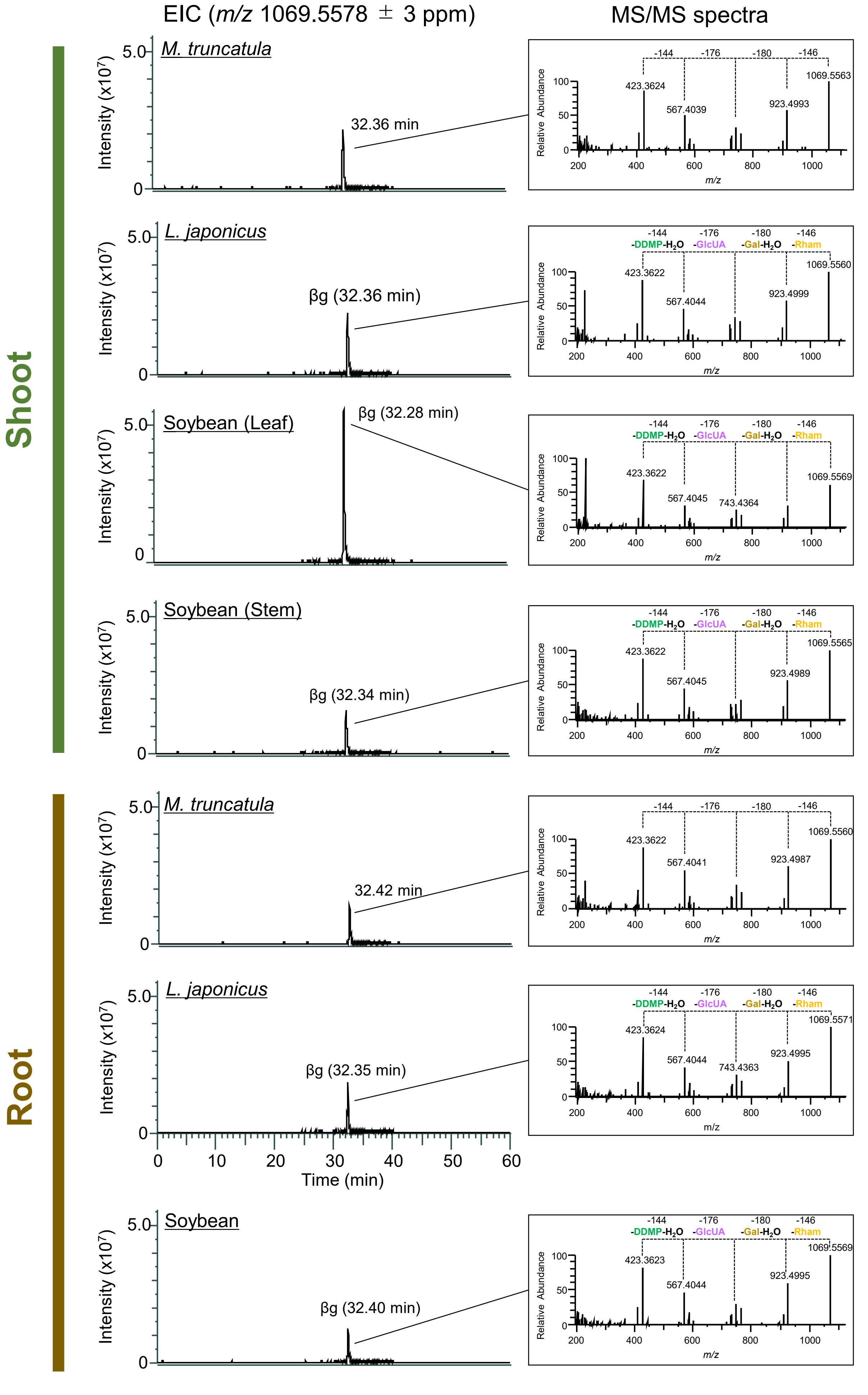

EIC (m/z 1069.5578 ± 3 ppm)
MS/MS spectra
Shoot
Root
M. truncatula
L. japonicus
Soybean (Leaf)
Soybean (Stem)
Soybean
Intensity (x10^7)
5.0
0
32.36 min
βg (32.36 min)
βg (32.28 min)
βg (32.34 min)
32.42 min
βg (32.35 min)
βg (32.40 min)
Time (min)
0
10
20
30
40
50
60
Relative Abundance
m/z
-144
-176
-180
-146
-DDMP-H2O
-GlcUA
-Gal-H2O
-Rham
423.3624
567.4039
923.4993
1069.5563
423.3622
567.4044
923.4999
1069.5560
567.4045
743.4364
1069.5569
923.4989
1069.5565
567.4041
923.4987
743.4363
923.4995
1069.5571
423.3623

Figure S15. LC-MS/MS analysis of crude extracts from the roots and shoots of *Medicago truncatula*, *Lotus japonicus,* and soybean.

The whole roots and shoots of *M. truncatula* and *L. japonicus* were sampled 18 and 30 days after imbibition, respectively. As plants at the R2 stage (Fehr et al., 1971) were used for soybean, it was not possible to sample the entire shoot. Instead, the leaves (fully expanded uppermost trifoliate leaves), stems (internodes immediately below the shoot apices) and roots (main roots, approximately 0–10 cm from the root tips, and the lateral roots that developed from them) were used separately. Samples were flash frozen in liquid nitrogen immediately after harvest and stored at -80ºC. Following ground into a fine powder using a Multibead Shocker MB1050 (Yasui Kikai, Osaka, Japan), the sample was suspended in 10 μl of ice-cold 70% ethanol per mg of sample, containing 100 ng/ml of α-hederin as an internal standard. After vigorous stirring at 4ºC for 1 h, the extract was centrifuged and the supernatant filtered through a 0.2 μm filter. The filtrate was diluted tenfold with the extraction solvent and analysed using LC-MS data-dependent analysis (DDA) with a Vanquish Flex system coupled to an Exploris 240 instrument (Thermo Fisher Scientific). One microlitre (10 mg tissue fresh weight equivalent) was injected and separated on an ODS column (Ascentis Express 90 Å AQ-C18 150 x 2.1 mm internal diameter, 2 μm particle size; Merck, Germany) at 50ºC with a flow rate of 0.3 ml/min. The solvent programme consisted of a linear gradient of 10% (v.v) (0–2 min), 10%–56% (v.v) (2–40 min) and 100% (40–50 min) acetonitrile containing 0.1% (v.v) formic acid in water. Heated electrospray ionisation was used as the ion source. The master scan was acquired in full scan mode at a resolution of 180,000 with a scan range of *m/z* 200–2000. Precursor ions were filtered in DDA acquisition, and a data-dependent MS/MS scan was acquired with higher energy collisional dissociation in normalised collision energy mode at 45,000 resolution. The MS chromatograms were displayed as extracted ion chromatograms (EICs) at 1069.5578 ± 3 ppm, which corresponds to the $[M+H]^+$ ion of soyasaponin βg ($C_{54}H_{84}O_{21}$, exact mass 1068.5505). The MS/MS spectra of soyasaponin βg and its corresponding compounds are shown on the right-hand side of each EIC. The names of the sugar moieties and DDMP are coloured as those in Figure 1.

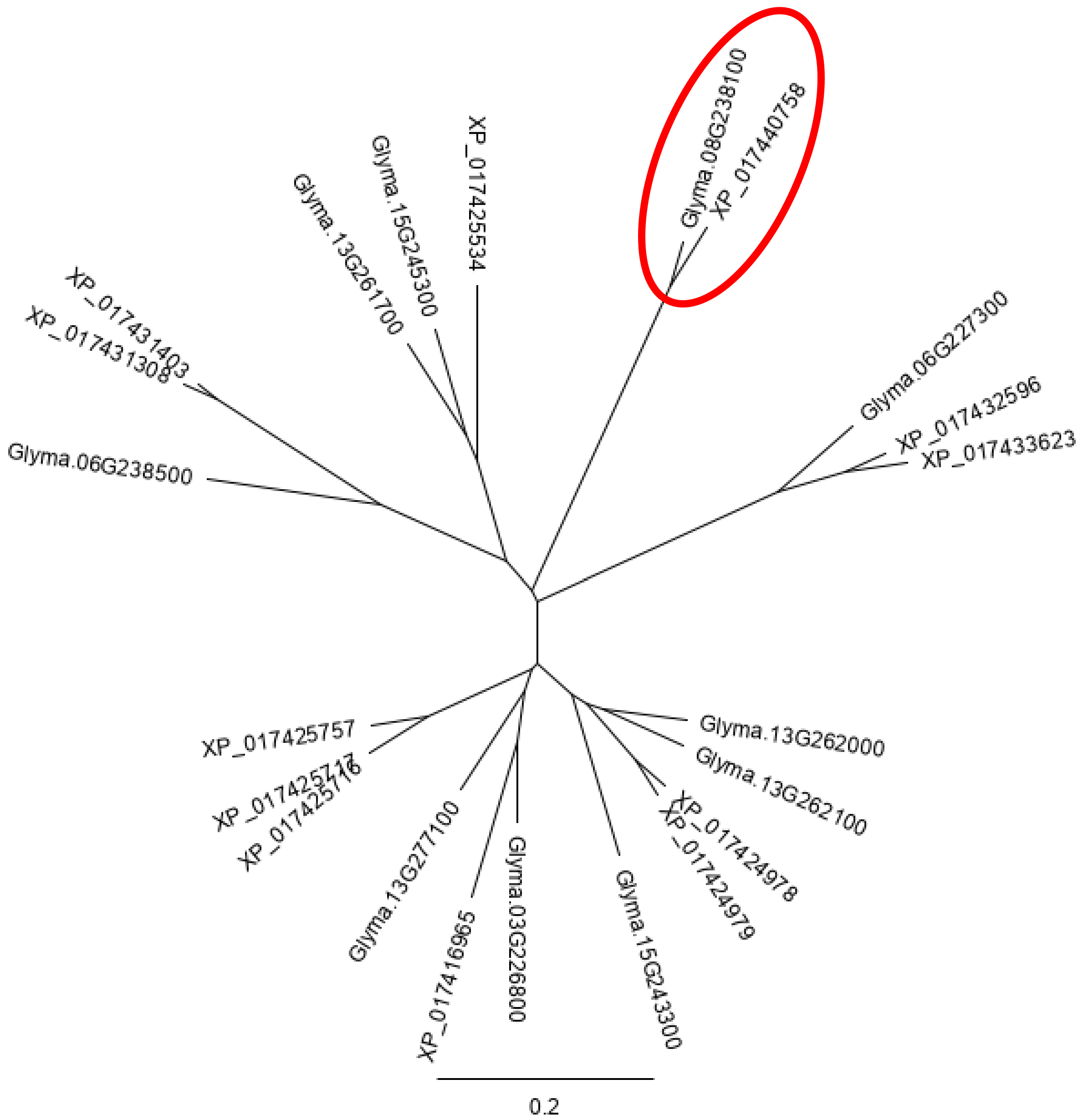

Figure S16. Phylogenetic tree of *CYP72A61* homologues from soybean and *Vigna angularis* (cultivar Longxiaodo No. 4).

The predicted amino acid sequence of *GmCYP72A61* was used as a query in BLASTP searches against Daizu-net (*Glycine max* cv. Enrei v3.31) and the NCBI (*V. angularis* cultivar Longxiaodo No. 4, GPIPE/3914/RS_2022_12 protein database) (Li et al., 2024). Nine *G. max* (Glyma) and twelve *V. angularis* (XP) sequences consisting of more than 450 amino acid residues and showing alignments with an e-value of 0 were selected for phylogenetic analysis. Soybean *CYP72A61* (*Glyma.08G238100*) and the most homologous *V. angularis* sequence (*XP_017440758*) are highlighted by a red circle. The scale bar shows amino acid substitution per site.

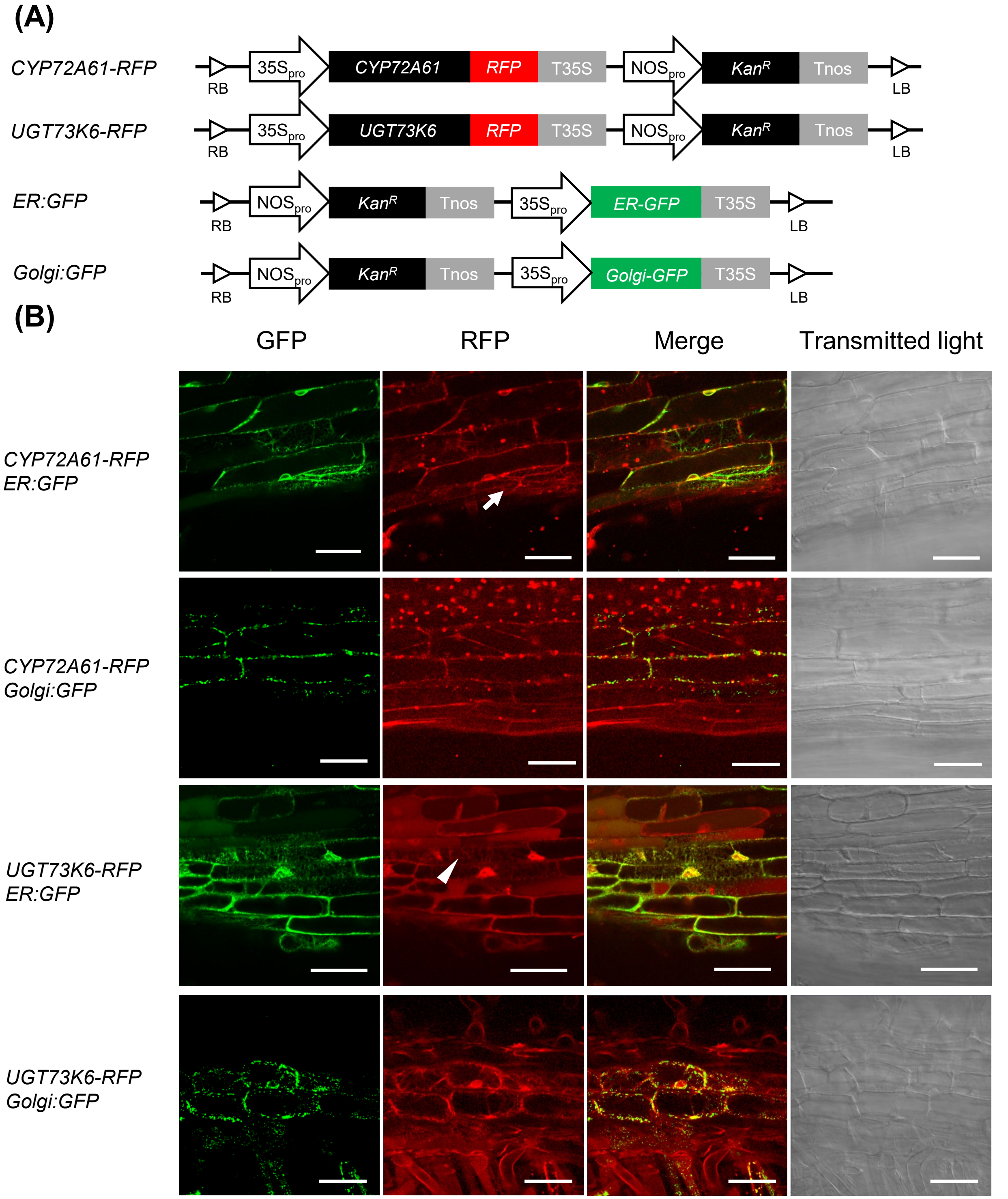

Figure S17. Subcellular localisation of UGT73K6 and CYP72A61 in *Lotus japonicus* hairy roots.

(A) Schematic of the T-DNA regions introduced into the hairy roots. Stop-codon-less *UGT73K6* and *CYP72A61* cDNAs were fused in frame with *RFP*. The following markers were used: an endoplasmic reticulum (ER) marker comprising the signal peptide from *Arabidopsis thaliana* wall-associated kinase 2 (AtWAK2) at the N-terminus of GFP and an ER retention signal (His-Asp-Glu-Leu) at its C-terminus; and a Golgi marker containing the cytoplasmic tail and transmembrane domain (first 49 aa) of soybean α-1,2-mannosidase I (GmMan1). These markers were used as described by Nelson et al. (2007). Abbreviations: RB: right border; 35Spro: CaMV 35S promoter; T35S: CaMV 35S terminator; NOSpro: *nopaline synthase* promoter; *KanR*: *neomycin phosphotransferase* coding sequence; Tnos: *nopaline synthase* terminator; LB: left border.
(B) Confocal images of UGT73K6-RFP and CYP72A61-RFP expressed in the hairy roots of the wild-type *L. japonicus* (ecotype Gifu B-129), co-expressed with ER-GFP and Golgi-GFP. Scale bars = 50 µm. The web-like reticulate pattern of the ER is indicated by arrows (for CYP72A61-RFP) and arrowheads (for UGT73K6-RFP).

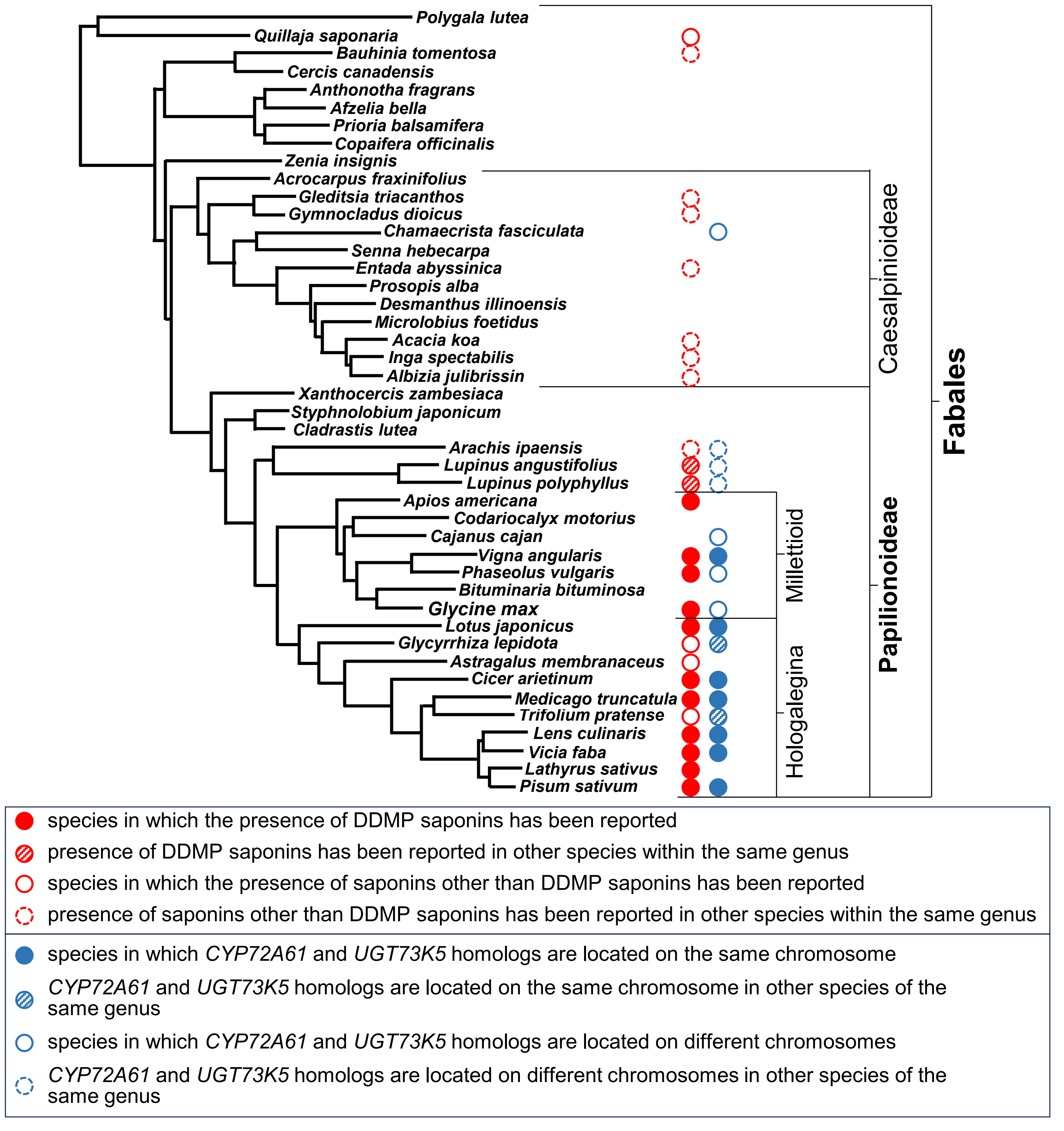

Figure S18. Distribution of saponins and genomic organisation of C-22 modification enzyme genes in leguminous plants.

The phylogenetic tree is adapted from the study by Koenen et al. (2021) and Choi et al (2022). The occurrence of DDMP and other saponins in the legumes included in the phylogenetic tree was assessed through literature surveys and experimental analysis (Fig. S15). Red circles indicate saponin distribution. Different symbols are used depending on whether DDMP or other saponins have been reported for the species shown or for other species within the same genus. Furthermore, BLASTP searches were performed on genome databases using predicted amino acid sequences of soybean *CYP72A61* and *UGT73K5* as queries (Table S11). Species in which homologues (≥50% amino acid identity) of both genes were identified are indicated by blue circles. The colouring of the blue circles differs depending on whether they are located on the same chromosome or on different chromosomes. See the legend below the phylogenetic tree for further details.

REFERENCES

Fehr, W.R., Caviness, C.E., Burmood, D.T. & **Pennington, J.S.** (1971) Stage of development descriptions for soybeans, *Glycine max* (L.) Merrill. *Crop Science*, **11**, 929-931.

Nelson, B.K., Cai, X. & **Nebenführ, A.** (2007) A multicolored set of *in vivo* organelle markers for co-localization studies in Arabidopsis and other plants. *The Plant Journal*, **51**, 1126-1136.
